# Intronic tRNAs of mitochondrial origin regulate constitutive and alternative splicing

**DOI:** 10.1186/s13059-020-02199-6

**Published:** 2020-12-08

**Authors:** Simon M. Hoser, Anne Hoffmann, Andreas Meindl, Maximilian Gamper, Jörg Fallmann, Stephan H. Bernhart, Lisa Müller, Melanie Ploner, Matthias Misslinger, Leopold Kremser, Herbert Lindner, Stephan Geley, Heiner Schaal, Peter F. Stadler, Alexander Huettenhofer

**Affiliations:** 1 Division of Genomics and RNomics, Biocenter, Medical University of Innsbruck, 6020 Innsbruck, Austria; 2 Helmholtz Institute for Metabolic, Obesity and Vascular Research (HI-MAG) of the Helmholtz Zentrum München at the University of Leipzig and University Hospital Leipzig, Philipp-Rosenthal-Str. 27, 04103 Leipzig, Germany; 3 Bioinformatics Group, Department of Computer Science and Interdisciplinary Center for Bioinformatics, Leipzig University, 04107 Leipzig, Germany; 4 Institute for Virology, Medical Faculty Heinrich Heine University Düsseldorf, 40225 Düsseldorf, Germany; 5 Division of Molecular Biology, Biocenter, Medical University of Innsbruck, Innsbruck, Austria; 6 Division of Clinical Biochemistry, Protein Micro-Analysis Facility, Biocenter, Medical University of Innsbruck, Innsbruck, Austria; 7 Institute of Pathophysiology, Biocenter, Medical University of Innsbruck, 6020 Innsbruck, Austria; 8 Max Planck Institute for Mathematics in the Sciences, Inselstraße 22, 04103 Leipzig, Germany

**Keywords:** nimtRNA, tRNA-lookalikes, Intronic splicing enhancer (ISE), Splicing regulatory element, numtDNA, tRNA, Mitochondrial tRNA, Splicing, Alternative splicing, Constitutive splicing

## Abstract

**Background:**

The presence of nuclear mitochondrial DNA (numtDNA) has been reported within several nuclear genomes. Next to mitochondrial protein-coding genes, numtDNA sequences also encode for mitochondrial tRNA genes. However, the biological roles of numtDNA remain elusive.

**Results:**

Employing in silico analysis, we identify 281 mitochondrial tRNA homologs in the human genome, which we term nimtRNAs (nuclear intronic mitochondrial-derived tRNAs), being contained within introns of 76 nuclear host genes. Despite base changes in nimtRNAs when compared to their mtRNA homologs, a canonical tRNA cloverleaf structure is maintained. To address potential functions of intronic nimtRNAs, we insert them into introns of constitutive and alternative splicing reporters and demonstrate that nimtRNAs promote pre-mRNA splicing, dependent on the number and positioning of nimtRNA genes and splice site recognition efficiency. A mutational analysis reveals that the nimtRNA cloverleaf structure is required for the observed splicing increase. Utilizing a CRISPR/Cas9 approach, we show that a partial deletion of a single endogenous nimtRNA^Lys^ within intron 28 of the *PPFIBP1* gene decreases inclusion of the downstream-located exon 29 of the *PPFIBP1* mRNA. By employing a pull-down approach followed by mass spectrometry, a 3′-splice site-associated protein network is identified, including KHDRBS1, which we show directly interacts with nimtRNA^Tyr^ by an electrophoretic mobility shift assay.

**Conclusions:**

We propose that nimtRNAs, along with associated protein factors, can act as a novel class of intronic splicing regulatory elements in the human genome by participating in the regulation of splicing.

**Supplementary Information:**

The online version contains supplementary material available at 10.1186/s13059-020-02199-6.

## Background

In the course of evolution, splicing has been demonstrated to be an increasingly important step in eukaryotic gene expression [1]. Orchestrated by spliceosomal complexes, intronic regions are excised from a pre-mRNA transcript, while exonic regions are joined [2]. Splice acceptor and donor sites define exon/intron borders, which is achieved by base complementarity to small nuclear RNAs (snRNAs), which are part of spliceosomal small nuclear ribonucleo-proteins (snRNPs) [2]. By means of splice site selection, a single pre-mRNA transcript can be employed to generate several distinct splice products, a process designated as alternative splicing. This is achieved by modulating splice site strength through *cis*-regulatory elements within the pre-mRNA transcript. These splicing regulatory elements (designated as SREs) are recognized by *trans*-acting proteins in a sequence- and structure-dependent manner which directly or indirectly interact with the spliceosome in a position-dependent manner [3, 4].

In addition to SREs, intronic ncRNAs such as microRNAs (miRNAs) and small nucleolar RNAs (snoRNAs) have been demonstrated to affect host gene splicing. Due to an interplay between spliceosomal components and miRNA processing enzymes, intronic miRNA processing has been shown to be able to counteract host pre-mRNA splicing *in cis* [5]. In contrast, processing of miRNA-211 was demonstrated to promote splicing of its hosting intron [6]. Additionally, intronic snoRNAs have been shown to be co-transcriptionally/pre-splicing bound by snoRNA processing enzymes, thus indicating a potential mechanism of interaction between intronic *cis*-acting snoRNAs and the spliceosome [7–10]. Accordingly, pre-mRNA splicing of NOP56, a component of canonical snoRNP complexes, is autoregulated *in cis* by the intron-hosted snoRNA SNORD86 [11].

While the majority of snoRNA genes and a large number of miRNAs are located within introns of nuclear protein-coding genes [12], nuclear tRNAs are generally transcribed by RNA polymerase III as independent transcription units, employing internal promoter sequences, i.e., boxes A and B, respectively. Nuclear-encoded tRNAs are transcribed as precursor sequences and are subsequently processed by two endonucleases, i.e., RNase P and RNase Z, at their 5′- and 3′-terminus [13], respectively, resulting in mature RNA species of approximately 70–90 nt in length [14].

In contrast to the nuclear genome, the human mitochondrial genome contains 22 mitochondrial tRNA genes (mtRNAs), interspersed between 13 protein-coding genes which predominantly encode for proteins of the respiratory chain. Three different polycistronic transcripts are generated by a single mitochondrial RNA polymerase [15]. Subsequent cleavage of these polycistronic transcripts mediated by the two tRNA processing enzymes (i.e., mitochondrial RNase P and RNase Z) generates mature mtRNAs in a process which concomitantly releases intervening mitochondrial mRNAs [13]. Both nuclear and mitochondrial tRNAs exhibit a characteristic, cloverleaf-shaped secondary structure, which among other functions is also important for their processing. However, while most mtRNAs still show a canonical cloverleaf structure, they lack some of the features that are highly conserved in nuclear tRNAs, in particular sequences characteristic of highly conserved D-loops and/or T-loops [16]. In some cases, they may also lack entire tRNA structural domains [17, 18]. Compared to nuclear tRNAs, mtRNAs show a different sequence bias and exhibit tertiary interactions distinct from nuclear-encoded tRNAs [19].

According to the endosymbiotic theory, eukaryotic mitochondria originated from the progressive transfer of ancient α-proteobacteria DNA into the eukaryotic genome [20]. Thus, the mitochondrial genomes of higher organisms are 100- to 300-fold smaller than bacterial genomes but still carry the hallmarks of a bacterial ancestor [21]. Interestingly, mammalian genomes harbor a large number of genomic regions designated as “nuclear mitochondrial DNA” (numtDNA) [22]. It can therefore be seen that the integration of numtDNA into the nuclear genome is a rapid and ongoing process [23] that is fast enough to render human haplotypes polymorphic for numtDNA. Insertions appear approximately uniformly across the genome [24] and are favored in locations exhibiting DNA curvature and adjacent to A/T oligomers [25]. They are enriched near retrotransposable elements [25], whose genomic distribution can be explained by random insertion and duplications [26]. In particular, numtDNAs do not appear in clusters and are not enriched on particular chromosomes [25]. Thus, insertions of numtDNA are independent, random events that serve no known purpose [27–29]. Nevertheless, a small number of numtDNA sequences have been implicated in human genetic diseases [30].

**Fig. 1:**
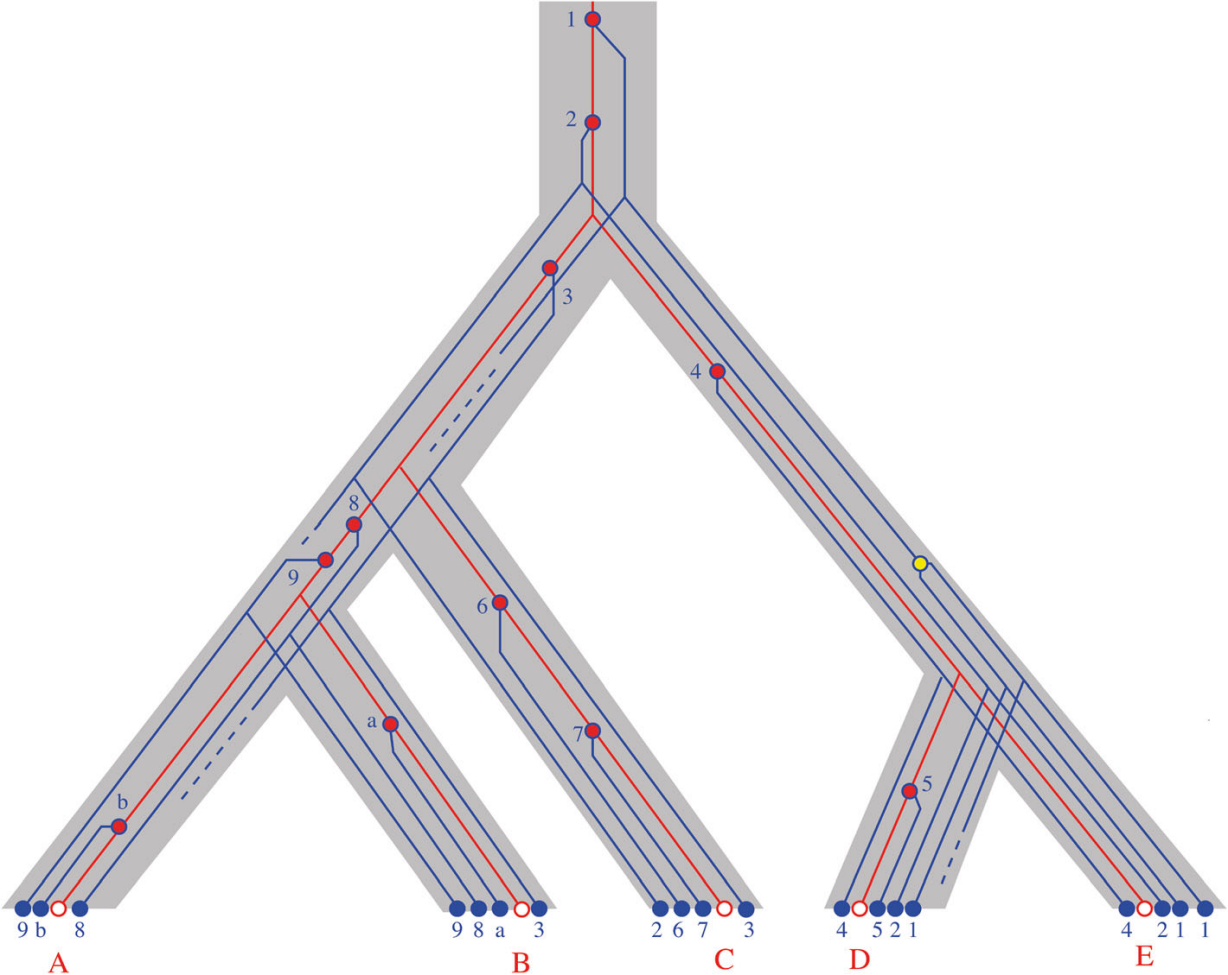
Evolution of mtRNAs and mtRNA insertions in the nuclear genome. The gray outline shows the phylogenetic relationship of five species A, B, C, D, and E as a dated tree, i.e., the “*y*-axis” corresponds to time before the present. Mitochondrial DNA, usually transmitted through the maternal lineages, faithfully follows the species tree (red tree); its leaves are the extant mtRNAs. Insertions of mitochondrial material occur at random time points independently in the different lineages (red circles). Following an insertion, the inserted material (numtDNA) evolves independently, as shown by blue trees within the species trees. Blue bullets denote the MTLs, and dashed lines indicate MTLs that have been removed by genomic events or that have mutated beyond the detection threshold. Each species contains MTLs that have been inserted at different times. Orthologous MTLs derive from the same insertion event (here denoted by the same blue numbers). Sequence comparison between MTLs or MTLs and mtRNAs shows the substitutions accumulated along the path between them. Orthologous MTLs are connected by blue path only, along which only selective pressures in the nuclear genome have left their traces. Comparisons between MTLs and mtRNAs in the same species almost always trace back to the insertion event. Their sequence differences thus record both the selective pressures acting on mtRNAs (red part of the path) and those acting on the inserted MTL (blue part of the path). Rare exceptions are duplications of MTLs after their insertion (yellow ball on the right). A comparison of arbitrary pairs of MTLs from different species in general corresponds to MTLs deriving from distinct insertion events. Their common history is a mixture of red paths (between the two insertion events) and blue paths (following the insertion events) and thus a mixture of selective pressures on mtRNA and MTL. These cannot be disentangled without exact dating of the insertion events

NumtDNAs display variations in size, the position of the fragment from which the numtDNA is derived in the mitogenome and in evolutionary age. At the time of insertion, the numtDNA sequence is identical to its counterpart in the mitogenome. Subsequent to its insertion, numtDNA and mitogenomic sequences evolve independently. The mitogenomic sequence (shown in red in Fig. 1) remains subject to the selection pressures in the mitochondrion. On the other hand, different fates are possible for numtDNA sequences: (i) The insertion disrupts cellular functions, the genome variant carrying the numtDNA is quickly removed by selection and no genomic record of the insertion event survives. (ii) In the most likely scenario, the newly inserted numtDNA does not affect the cell’s functions and is hence, from an evolutionary standpoint, neutral. In this case, the numtDNA accumulates substitutions at the same rate as other neutrally evolving DNA sequences. This process is slow enough for numtDNA sequences to remain recognizable by sequence similarity on timescales comparable to the radiation of the placental mammals. Eventually, however, all traces of an ancient numtDNA insertion are eradicated by the accumulation of random mutations. (iii) In some cases, numtDNA sequences and in particular the mtRNAs contained within them may acquire novel functions in the nuclear genome. In this case, the functional sequence is subject to the influence of stabilizing selection for its new function and persists in the nuclear genome. Occasional duplications of numtDNA in the nuclear genome can further complicate the picture [31].

MtRNA genes are inserted into the nuclear genome as components of numtDNAs. The nuclear genome had previously been scanned for full-length mitochondrial tRNAs, which were named mitochondrial tRNA-lookalikes (MTLs) [32, 33]. The analysis of selection pressures acting on MTLs must therefore take their peculiar evolutionary history into account. In this context, we face two important issues: (i) A comparison of different MTLs with the corresponding mtRNAs must take into account that the insertion events potentially occurred at different points in time (consider, e.g., the three numtDNAs 8, 9, and b versus the mtRNA of species A in Fig. 1). The sequence divergence of MTL and mtRNA confounds the selection pressure on the mtRNA within the mitochondrion since the insertion event with the selective effects the MTL may have experienced in the nuclear genome. This can be accounted for by using the surrounding sequence of the numtDNA as a “molecular clock” that implicitly has recorded the insertion time. (ii) When comparing two MTLs that derive from different insertion events of the same mtRNA, the degree of sequence divergence is a composite function of the selection pressures faced by the two numtDNAs after insertion and selection pressures operating in the mitochondrion on the mtRNA between the first insertion event and the second (consider, e.g., the path from numtDNA 6 to numtDNA 7 in species B, which involves the red (mitochondrial) segment between insertion events 6 and 7 as well as the blue (numtDNA) segments connecting the numtDNAs to the insertion events). As a consequence, MTL/MTL comparisons within the same genome cannot separate selection pressures acting in the nuclear genomes from selection pressures in the mitochondrion. To overcome this problem, one has to identify orthologous MTLs in different species, i.e., MTLs that derive from the same numtDNA insertion event. These can be identified reliably by considering homology of DNA outside of the inserted numtDNA to determine syntenic MTLs. It should be noted, however, that they cannot be identified by simple sequence comparison, since all MTLs with the same codon are homologs and sequences that were inserted more recently by distinct events will be more similar to present-day mtRNAs than old orthologous MTLs. This is because mtRNAs (red paths) evolve more slowly than inserted MTLs (blue paths), as long as an inserted MTL has not acquired a new function in the nuclear genome, which would subject it to strong negative selection and thus results in its conservation. Only sufficiently old numtDNA insertions, namely those that pre-date speciation events that separate species with sequenced genomes, therefore are informative of selective pressures on MTLs as revealed by direct comparison of MTLs.

As a consequence, we therefore have to focus on MTLs that are embedded in recognizable larger numtDNA sequences. This also allows us to distinguish bona fide MTLs from degraded copies of nuclear tRNAs or tRNA-associated short interspersed nuclear elements (SINEs) [34], which, at least for old insertions, cannot be separated cleanly on sequence similarity scores alone.

Notably, MTL sequences often differ substantially from their mitochondrial counterparts. Also, MTLs of the same tRNA isotype can vary extensively in their sequence. In the human genome, there are only eight MTLs that are still identical in sequence to their primordial mtRNA counterparts, while the remaining 489 MTLs show up to 25 mismatches [32]. At present, the biological function and relevance of MTLs is still unknown. About 20% of known human MTLs have been reported by the group of Telonis and co-workers to be located in introns of protein-coding or noncoding RNA transcripts [32].

Currently, a single MTL annotation strategy was published [32, 33] based on a BLAST search of the known nuclear and mitochondrial tRNA sequences against the nuclear genome with the intention of identifying full-length tRNA-like sequences in the nuclear genome. Since structural conservation is not included in this previous approach, MTLs that have diverged at their sequence level but may have retained tRNA-like structures are not annotated. Applying the computational annotation workflow presented here, we were able to identify numerous novel MTLs and nuclear-encoded intronic mitochondrial-derived tRNA genes (designated as nimtRNAs) in humans and mice. Notably, nimtRNAs were always flanked by sequences of mitochondrial origin.

Strikingly, the canonical tRNA secondary structure was conserved as observed mutations relative to their mitochondrial counterparts were found either in loop regions or as compensatory base changes in stem domains. In this study, we thus aimed to investigate the potential function(s) of nimtRNAs located within the introns of nuclear-encoded pre-mRNAs. We demonstrate that nimtRNAs interact with specific RNA-binding proteins (RBPs) and participate in regulation of splice site usage by a mechanism comparable to that of bona fide SREs.

## Results

### Numerous, so far unidentified nimtRNAs are present in nuclear genomes

To scan, in particular, the human and mouse genomes for MTL sequences, we applied different combinations of annotation tools (tRNAscan-SE and Infernal) and strategies (*NUMT*-based and *genome*-based), see Additional File 1: Fig. S1A. Within the *NUMT*-based approach (using published numtDNA sequences as reference only) for the human genome, we obtained 775 hits from Infernal [35] and 726 hits from tRNAscan-SE. In contrast, the *genome*-based approach (using the whole genome as reference), we received 367 hits, only, from Infernal, whereas tRNAscan-SE [36] scored about 2.65 times more hits (977 hits). The analysis of the mouse genome yielded very similar results. We got 105 hits from Infernal and 79 hits from tRNAscan-SE within the *NUMT*-based approach. The hits from the *genome*-based approach vary from 75 (Infernal) to 246 (tRNAscan-SE).

Since for each numtDNA the original mtDNA sequence is known, we used this synteny information to validate our results. For each method, we classified the detected hits as true positives (TPs) if they were found in the corresponding numtDNA as described by their synteny of the originating mitochondrial DNA. The remaining hits were designated as false positives (FPs). As shown in Additional file 1: Fig. S1A, Infernal found 2% more TPs in human than tRNAscan-SE (true positive rate (TPR) of 0.91) within the *NUMT*-based approach. Despite the lower sensitivity of tRNAscan-SE, the tool counts only 29 false positives (FPs) compared to the 68 FP hits of Infernal. The difference is even more pronounced in the *NUMT*-based approach for mouse, where Infernal identified 13% more TPs, but also 11% more FPs compared to tRNAscan-SE. tRNAscan-SE shows the highest sensitivity in the *genome*-based approach with a TPR of 0.88 and 0.72 in human and mouse, respectively. Infernal delivers much less TP in both species for the *genome*-based method. In both the *NUMT*- and the *genome*-based approach, tRNAscan-SE shows the best balance between TPs and FPs. For downstream analysis, the final MTL set is composed of all detected TPs (MTLs within recognizable numtDNA) regardless of the method and tool used.

**Fig. 2:**
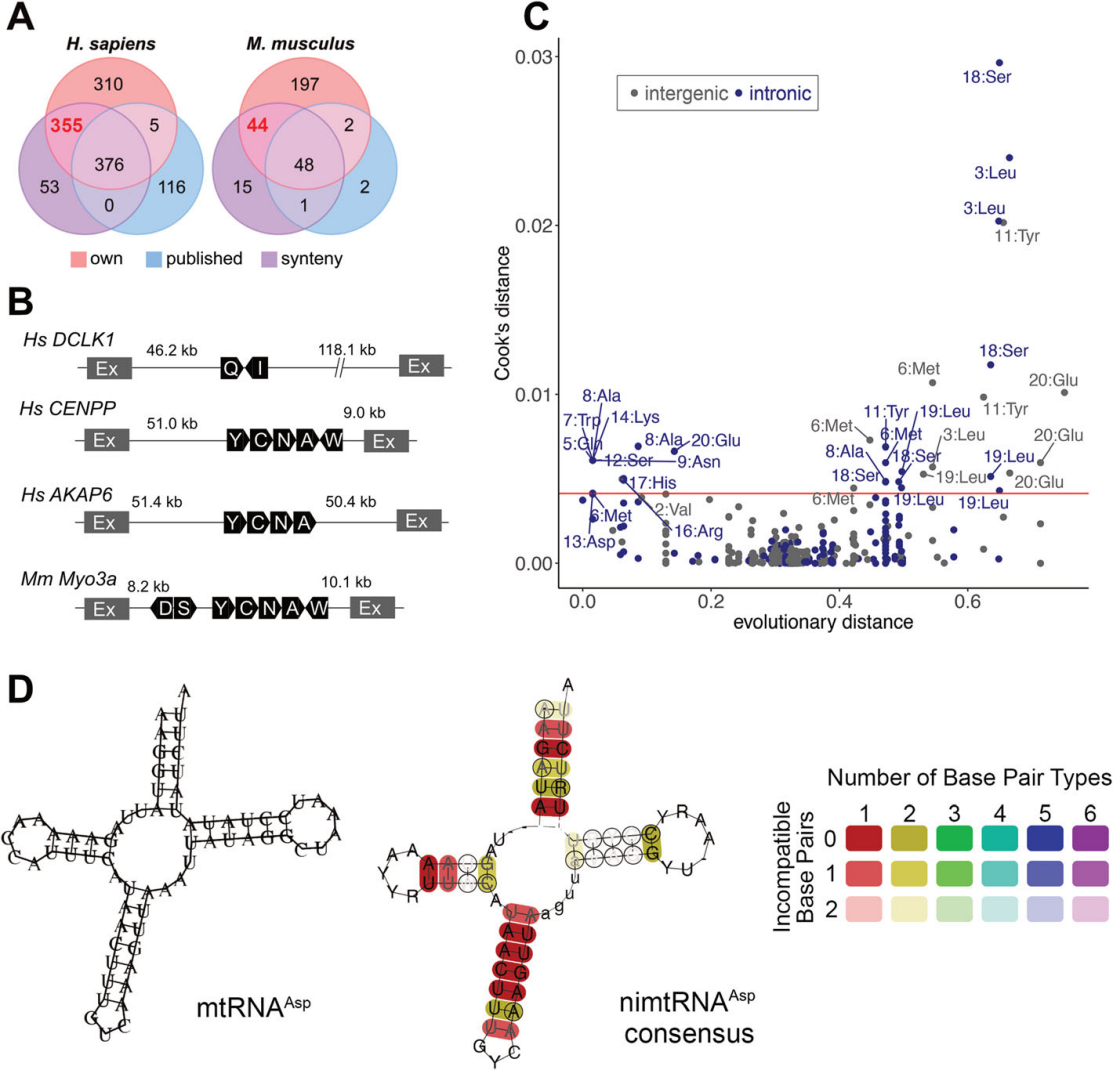
Computational analyses of MTLs within the human and mouse genome. **a** Overview of the annotated MTLs in humans and mice. Venn diagram illustrating the overlap between our annotated (own) MTLs and those that are already known (published), as well as which of them are located within previously annotated numtDNAs (synteny). Although the synteny information indicates which mtRNAs have been integrated into the nuclear genome, not all of them can be annotated due to their strong degradation (53 in human and 15 in mouse). In total, we identified 731 and 92 MTLs within recognizable numtDNA in humans and mice, respectively. Of these, 355 are newly annotated in humans and 44 in the mouse. **b** The genes *DCLK1*, *CENPP*, and *AKAP6* in *H. sapiens* and the gene *Myo3a* in *M. musculus* were analyzed by tRNAscan. Additionally, the 5′- and 3′-distances from nimtRNA clusters to the intron termini were determined. White letters in black boxes represent the single letter amino acid code of the respective nimtRNA (Q = nimtRNA^Gln^, I = nimtRNA^Ile^, Y = nimtRNA^Tyr^, C = nimtRNA^Cys^, N = nimtRNA^Asn^, A = nimtRN^Ala^, W = nimtRNA^Trp^, D = nimtRNA^Asp^, S = nimtRNA^Ser^). **c** Evolutionary conserved MTLs. Outliers of MTLs are subject to a stronger stabilizing selection after their insertion into the nuclear genome relative to numtDNAs and are shown above the red line. Outliers were measured by Cook’s distance. The majority (25 of 36) of the more extreme outliers are nimtRNAs. **d** Preservation of the secondary structure of nimtRNAs. As an example, multiple sequence alignments along with the consensus sequence-structure RNA motif are shown for all nimtRNAs of type nimtRNA^Asp^. The sequences of nimtRNA^Asp^ exhibit base changes compared to their primordial mtRNA^Asp^, but the secondary structure is maintained. The different colors provide information concerning the number of distinct base pairs occurring whereas the shading indicates how many sequences or structures in the alignment do not form a particular base pair

Finally, we identified 731 MTLs within recognizable numtDNA (42 MTLs (*NUMT*-based method) + 684 (*NUMT*- and *genome*-based method) + 5 (*genome*-based method)) and 92 MTLs within recognizable numtDNA (16 MTLs (*NUMT*-based method) + 73 (*NUMT*- and *genome*-based method) + 3 (*genome*-based method)) in human and mouse genomes, respectively (Fig. 2a). Thereof are 355 MTLs in human and 44 MTLs in mouse novel discoveries. Our MTL annotation strategy is more sensitive (TPR of 0.93 in human and 0.85 in mouse) compared to previous MTL annotations (TPR of 0.48 in human and 0.47 in mouse) [32, 33] (Additional file 1: Fig. S1A). Previous computational studies have demonstrated that about 20% of the MTLs are located within introns which we designate as nimtRNAs of nuclear protein-coding genes in humans [33, 37]. We observed comparable results with our analysis. In humans, we identified a total of 281 nimtRNAs of all types in the introns of 76 different host genes, of which 30 were protein-coding, 28 were specifying long intergenic noncoding RNAs (lincRNAs), 13 were coding for short ncRNAs, and 5 were pseudogenes. In total, 121 of the identified nimtRNAs in human are novel. Compared to previous surveys [33, 37], we identified 12 novel nimtRNAs (of total 34) in 11 different host genes (9 different protein-coding genes and 2 different lncRNAs) in the mouse. A complete list of all annotated MTLs and nimtRNAs found in mice and humans can be obtained from Additional file 2: Table S1 and Additional file 3: Table S2, respectively.

### Conservation of MTLs and nimtRNAs

In several cases, large clusters of nimtRNA genes with extensive sequence similarities to the mitochondrial genome were present within introns of nuclear genes but were completely absent from exonic regions. Four examples of nimtRNA host gene introns in humans and one in mouse are shown (Fig. 2b). The observed mitochondrial clusters are located in different genes in the mouse when compared to the human genomes. We found a similar degree of evolutionary conservation between nimtRNAs and the corresponding mitochondrial sequences among different mammals. Since their PhyloP scores are very low, the majority of MTLs within recognizable numtDNA show no evidence of negative selection in the host genomes. While we found that PhyloP scores are slightly enhanced in MTLs within recognizable numtDNA and nimtRNAs compared to the surrounding numtDNA sequences (Additional file 1: Fig. S1B and C), the selection pressures are insufficient to identify individual MTLs within recognizable numtDNA or nimtRNAs that are under strong negative selection. Instead, only a few shorter elements are conserved. We interpret these as possible binding sites that have emerged from the inserted mtRNA sequence. Using a different method, we identified about a dozen MTLs within recognizable numtDNA and nimtRNAs that appear to have evolved significantly more slowly than the adjacent numtDNA sequences. It is also interesting to note nimtRNAs represent the majority of the more extreme outliers (Fig. 2c) as measured by Cook’s distance. All these outliers are listed in Additional file 4: Table S3.

Based on the consensus structure of each type of nimtRNA, it is apparent that base changes in most nimtRNA types were located either in loop regions of tRNAs or, in several cases, were present in the form of compensatory base changes in stem structures (Fig. 2d, Additional file 5: Table S4). Accordingly, in most consensus structures, the mitochondrial secondary structure is largely retained and thus probably also their function (see below). Evolutionary conserved compensatory base changes are consistent with a functional role of nuclear-encoded nimtRNA genes. In a few cases, the consensus structures deviate strongly from their primordial mtRNAs. This is probably one reason why we cannot find all expected MTLs within a numtDNA, as is the case for mtRNA^Pro^ in particular.

Taken together, the insertion of nimtRNA genes in the respective introns of nuclear genes might be a very recent evolutionary event, which might have occurred independently in different species in addition to potential retainment of pre-existing nimtRNAs. Furthermore, this computational analysis points to MTLs within recognizable numtDNA and nimtRNAs as a source of functional binding sites. As expected in such a scenario, most MTLs within recognizable numtDNA and nimtRNAs have not attained functional significance because they are simply not present in a useful genomic context or there is no selective advantage to be gained from an MTL within recognizable numtDNA- or nimtRNA-derived binding site at the position of the insertion.

### NimtRNAs located in introns of nuclear-encoded pre-mRNAs are not processed as bona fide tRNAs in 293T cells

**Fig. 3:**
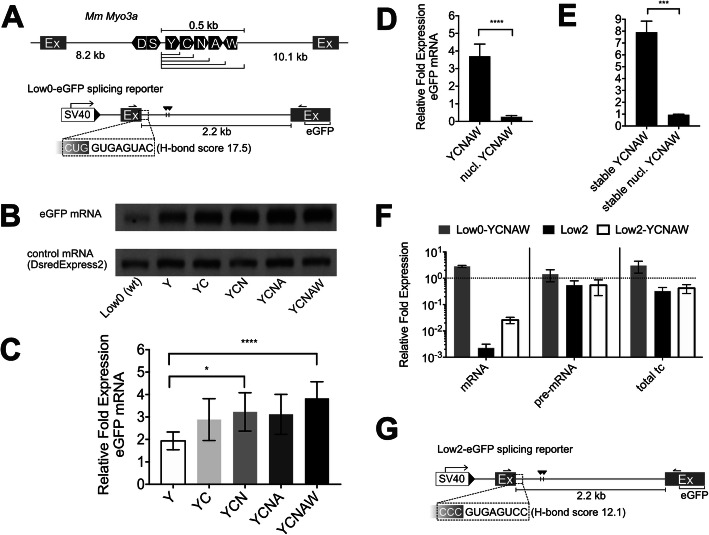
NimtRNAs increase host gene mRNA abundance by increasing splicing efficiency. **a** Black boxes represent the single letter amino acid code of the respective nimtRNA: one nimtRNA (Y), two (YC), three (YCN), four (YCNA), or five (YCNAW) nimtRNAs from the mouse *Myo3a* intron 30 were cloned into the intronic region of the Low0-eGFP splicing reporter, exhibiting an efficient 5′-splice site. NimtRNA genes were inserted as indicated by triangles. **b** Northern blot analysis of eGFP mRNA constructs as depicted in **a** performed with digoxigenin (DIG)-labeled probes. Normalization of RT-qPCR and northern blot analyses was performed to Dsred2Express transfection control mRNA. Experiments were performed in triplicates. **c** RT-qPCR analysis of constructs as depicted in **a** was performed with the forward primer binding to exon 1 and the reverse primer binding to exon 2, depicted by black arrows shown in **a**. **d** Five nuclear tRNA genes (designated as nucl. YCNAW) were inserted into the Low0-eGFP splicing reporter and their influence on splicing was compared to the reporter containing five nimtRNAs (YCNAW). The Low0-eGFP splicing reporter was taken as a reference. **e** Stable cell lines were generated containing a single copy of the Low0-eGFP splicing reporter and either five nimtRNAs or five nuclear tRNAs. Normalization was performed to β-Actin. The stably integrated Low0-eGFP splicing reporter was taken as reference. **f** mRNA, pre-mRNA, and total transcript (mRNA+pre-mRNA) levels from cells transfected with the respective constructs were assessed by RT-qPCR. Error bars represent the standard deviation from the mean of three independent experiments. **g** The Low2-eGFP splicing reporter differs from the Low0-eGFP splicing reporter by possessing a less efficient 5′-splice site as indicated. Averages and standard deviations were determined from three independent sets of experiments. Error bars represent the SD and *P < 0.05; ***P < 0.001; ****P < 0.0001 (ANOVA)

Mitochondrial- as well as nuclear-encoded tRNAs are post-transcriptionally processed by RNase P and RNase Z at their 5′- and 3′-terminus, respectively (see above). In order to more closely investigate a potential cleavage, processing and function of nimtRNAs, we employed an eGFP splicing reporter, designated as Low0-eGFP, consisting of a noncoding exon, a 2.2-kb-long intron and a second exon, containing the coding sequence for the enhanced green fluorescent protein (eGFP; Fig. 3a) [38].

A cluster of five nimtRNA genes (out of the seven nimtRNAs of the *Myo3a* gene, Fig. 3a), previously reported to be present within intron 30 of the mouse *Myo3a* gene [33], was inserted into the intronic region of this splicing reporter. The rationale for not including all seven nimtRNAs was based on the fact that two nimtRNAs, i.e., nimtRNA^Asp^ and nimtRNA^Ser^, respectively, are located 1.5 kb upstream from the cluster of five nimtRNAs, and the insertion of a region spanning additional 1.5 kb might have impaired canonical reporter splicing. Thereby, the mouse nimtRNA cluster exhibited distinct sequence differences compared to bona fide human mtRNAs or human nimtRNAs thus permitting their specific detection by northern blot analysis. HEK 293T cells were transiently transfected with the splicing reporters either lacking or containing nimtRNAs. The abundance and/or processing of nimtRNAs, i.e., nimtRNA^Tyr^, nimtRNA^Cys^, and nimtRNA^Asn^, from the *Myo3a* nimtRNA cluster was investigated by northern blotting. However, no hybridization signal was detected corresponding to fully processed nimtRNAs of about 70 nt in length (Additional file 1: Fig. S2A).

We thus next investigated whether processing of another reported intron-encoded ncRNA, i.e., a snoRNA within the eGFP splicing reporter construct was also hampered. To that end, we cloned the gene of a brain-specific ncRNA, i.e., the C/D box snoRNA SNORD115 including flanking regions into the identical intronic location. In contrast to nimtRNAs, a hybridization signal of the expected size for the processed SNORD115 RNA species could be readily observed (Additional file 1: Fig. S2B).

In order to address the discrepancy in canonical processing of a snoRNA, compared to nimtRNAs, we employed an intron-less Pol III reporter containing a single copy of nimtRNA^Asn^ from the *Myo3a* gene. Consistent with our expectations, we observed a fully processed and stable nimtRNA^Asn^ by northern blot analysis (Additional file 1: Fig. S2B), excluding the possibility that nimtRNAs are degraded within the nucleus. In addition to nimtRNA sequences, we also investigated processing of nimtRNAs from their endogenous host gene transcripts. As observed for the eGFP splicing reporter, however, we also failed to detect processed nimtRNAs from the endogenous *DYNC2H1* host gene.

### The role of nimtRNAs in pre-mRNA metabolism

To thus investigate alternative functions of nimtRNAs, we inserted either one, two, three, four, or five nimtRNA genes from the *M. musculus Myo3a* intron, i.e., nimtRNA^Tyr^, nimtRNA^Cys^, nimtRNA^Asn^, nimtRNA^Ala^, and nimtRNA^Trp^, into the intron of the Low0-eGFP splicing reporter construct employed above (Fig. 3a). Subsequently, by northern blot and RT-qPCR analysis, we investigated their influence on pre-mRNA splicing of the eGFP host gene. Interestingly, insertion of nimtRNAs into the eGFP reporter gene resulted in a significant increase in the abundance of spliced eGFP mRNA levels, compared to a control lacking the nimtRNA genes (wt; Fig. 3b, c).

Importantly, cells transfected with eGFP constructs containing one (Y), two (YC), three (YCN), four (YCNA), or five nimtRNAs (YCNAW) exhibited a copy number-dependent increase of spliced eGFP mRNA levels of 1.9-fold, 2.9-fold, 3.2-fold, 3.1-fold, and 3.9-fold respectively, as assessed by northern blot and RT-qPCR analysis (Fig. 3b, c). Surprisingly, nimtRNA^Trp^, which is present in reverse-complementary orientation in the Low0-eGFP splicing reporter (as also observed within the mt genome), also increased reporter mRNA abundance. Normalization of eGFP mRNA levels was performed by employing a co-transfected plasmid (control mRNA), coding for a red fluorescent protein (DsredExpress2). The increase in mRNA levels was accompanied by an increase in eGFP protein level, as assessed by measuring eGFP fluorescence levels, normalized to DsredExpress2 (Additional file 1: Fig. S3A).

In the above experiments, intronic sequences containing single or multiple nimtRNAs were introduced into the intron in addition to the wildtype sequence rather than by substitution. To exclude a potential influence of intron size and/or intron structure on mRNA abundance, an artificial insert (of the same length as YCNAW), containing five nuclear tRNAs, was cloned into the same splicing reporter. Thereby, five nuclear nimtRNA counterparts, i.e., tRNA^Tyr^, tRNA^Cys^, tRNA^Asn^, tRNA^Ala^, and tRNA^Trp^, were inserted into the Low0 splicing reporter and analyzed for their effect on host gene splicing. Of note, nuclear tRNAs, although encoding for the same amino acids as nimtRNAs, differ extensively in their sequences from their nimtRNA homologs. In contrast to nimtRNAs, however, the nuclear YCNAW cluster construct resulted in a significant decrease, rather than an increase in mRNA abundance, pointing towards specific sequence or structural features of nimtRNAs (see below) that govern the observed increase in mRNA levels (Fig. 3d).

The above results were also corroborated by introducing the Low0-eGFP splicing reporter into stable cell lines. To that end, the Low0-eGFP splicing reporter was cloned downstream from an EF1α promoter and inserted as a single copy by Flippase recombination into HEK 293 Flip-In cells [39]. The reporter intron thereby contained either no tRNAs, five nimtRNAs from the *Myo3a* gene, or their nuclear tRNA homologs (see above). By RT-qPCR analysis, we observed an even higher abundance (i.e., 7.9-fold, compared to 3.9-fold in transiently transfected cells) of spliced eGFP mRNA levels in the cell line containing intronic nimtRNAs compared to the cell lines containing either the original intronic sequence or the nuclear tRNAs (Fig. 3e).

### NimtRNAs increase mRNA abundance by enhancing splicing efficiency

Next, we wanted to determine whether increased transcription or pre-mRNA processing was responsible for the nimtRNA-mediated increase in spliced eGFP mRNA levels. Thus, we investigated whether unspliced and total transcript levels (i.e., spliced and unspliced levels combined) of the Low0-eGFP splicing reporter were also affected by nimtRNAs. Upon intronic insertion of nimtRNAs into the Low0-eGFP splicing reporter (designated as Low0-YCNAW), by RT-qPCR analysis we observed an approximately 3-fold increase in spliced mRNA abundance as well as in total transcript levels, while pre-mRNA levels remained unchanged (Fig. 3f), consistent with a nimtRNA-mediated increase in splicing.

The first step of spliceosome assembly comprises the recognition of the 5′-splice site by the U1 snRNA [2]. The recognition of weak splice sites, i.e., those displaying low U1 snRNA complementarity, is known to be more dependent on SREs. Thus, we reduced the strength of the 5′-splice site (i.e., U1 snRNA complementarity: H-bond score (HBS) 17.5 > 12.1; designated as Low2-eGFP splicing reporter) and compared unspliced and total transcript levels. Notably, the HBS of this splice donor is still within the range of 12.0 to 20.0, which is observed in 86% of human constitutively spliced exons [40] (Fig. 3g).

As expected, the Low2 wt reporter resulted in an extensive, i.e., 450-fold, decrease in reporter mRNA abundance, compared to the more efficient Low0 wt reporter, while only an about 2-fold reduction in pre-mRNA and total transcript levels was observed. Upon nimtRNA insertion into the Low2 reporter (designated as Low2-YCNAW), spliced mRNA abundance was about 38-fold lower compared to the Low0 wt construct. Thus, insertion of the YCNAW nimtRNA cluster resulted in an about 13-fold increase in spliced mRNA abundance in the inefficient Low2 splicing reporter, hence exhibiting a more pronounced effect on splicing than the efficient Low0 splicing reporter (showing an about 3-fold increase in mRNA abundance). Notably, pre-mRNA and total reporter transcript levels remained unchanged (Fig. 3f). This can be explained by reporter pre-mRNA being significantly more abundant than spliced mRNA.

### Single nimtRNAs differently increase host mRNA levels

**Fig. 4:**
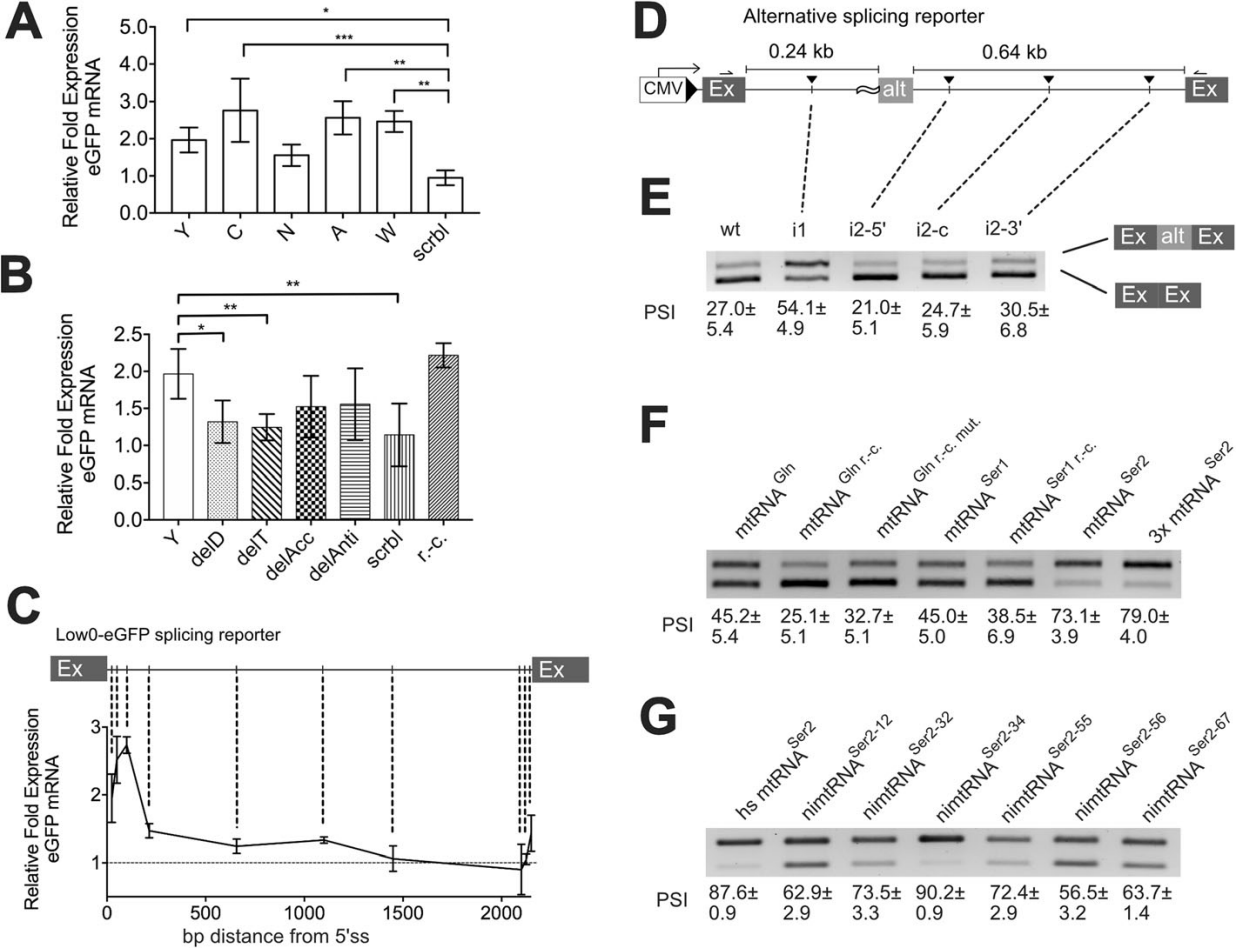
NimtRNAs increase downstream exon inclusion in a structure- and position-dependent manner. **a** Single nimtRNAs were analyzed for their effects on reporter host gene mRNA abundance compared to a scrambled control (scrbl). **b** Different domains of nimtRNA^Tyr^ were deleted, i.e., the D-arm (delD), the T-arm (delT), the acceptor stem (delAcc), or the anticodon arm (delAnti), respectively. Alternatively, nimtRNA^Tyr^ was exchanged for a scrambled sequence (scrbl), or inserted into the splicing reporter in reverse-complementary orientation (r.-c.). **c** NimtRNA^Tyr^ was integrated into different locations within the intron of the Low0-eGFP splicing reporter. Respective host gene mRNA abundances were assessed by RT-qPCR, error bars represent the standard deviation from three independent experiments. Normalization was performed relative to a co-transfected reporter control, i.e., Dsred2express. **d** The nimtRNA^Tyr^ was inserted into different locations within the introns of the alternative splicing reporter and used for transient transfection experiments. **e** Alternative splicing isoforms were analyzed by RT-PCR and subsequent gel electrophoresis. PSI (percent spliced in) including standard deviation was quantified from three independent sets of experiments. **f**, **g** RT-PCR analysis was performed to detect alternative splicing isoforms of constructs containing different mtRNAs or nimtRNAs within the first intron of the Designer Exon. PSI including standard deviation was quantified from three independent sets of experiments

Analysis of pre-mRNA splicing demonstrated a nimtRNA copy number-dependent increase in mRNA abundance. Hence, to investigate the effect of single nimtRNAs on mRNA abundance, nimtRNAs from the *Myo3a* gene were individually inserted into the Low0-eGFP splicing reporter and assessed for their influence on splicing. In this context, it was observed that nimtRNA^Tyr^ (Y), nimtRNA^Cys^ (C), nimtRNA^Ala^ (A), and nimtRNA^Trp^ (W) significantly increased eGFP mRNA levels by 1.9-fold, 2.8-fold, 2.6-fold, and 2.5-fold, respectively, compared to a scrambled control (Fig. 4a). Interestingly, nimtRNA^Trp^, which is present in a reverse-complementary orientation in its host intron (i.e., as present within the mt genome), also increased mRNA abundance (see above).

Mutational analysis by deletion of canonical tRNA domains within nimtRNA^Tyr^, i.e., the D-arm (delD), the T-arm (delT), the acceptor stem (delAcc), or the anticodon arm (delAnti), respectively, resulted in a decrease in mRNA abundance for all mutant versions of nimtRNA^Tyr^. NimtRNA^Tyr^ was chosen because we observed that the secondary structure of nimtRNA^Tyr^ homologs is well conserved in the human and the mouse genome. The most prominent decrease in splicing was observed upon deletion of the T-arm within nimtRNA^Tyr^ (Fig. 4b). As expected, scrambling the nimtRNA^Tyr^ sequence (designated as scrbl) failed to significantly increase eGFP mRNA levels. Interestingly, in contrast to the scrbl control, the reverse-complementary version of nimtRNA^Tyr^ designated as nimtRNA^Tyr r.-c.^, resulted in an increase in eGFP mRNA abundance comparable to that observed using its canonical counterpart.

### NimtRNAs affect pre-mRNA splicing dependent on their relative position within an intron

Splicing has been shown to be modulated in a position-dependent manner by splicing regulatory elements (SREs) [3]. Therefore, we investigated the effect of nimtRNA positioning on splice site recognition. Thus, we introduced either the YCNAW nimtRNA cluster or a single nimtRNA^Tyr^ (Y) at different locations within the intron of the Low0-eGFP splicing reporter. We observed the strongest increase in mRNA abundance/splicing when inserting the nimtRNA cluster 200 bp downstream of the 5′-splice site as compared to an insertion in the center of the 2.2-kb-long intron or 200 bp upstream of the 3′-splice site (Additional file 1: Fig. S3B and C). Upon insertion of the single nimtRNA^Tyr^ at different intronic locations, we observed the strongest increase in splicing efficiency for insertions 50 to 100 bp downstream of the 5′-splice site (Fig. 4c). Thus, we conclude that the increase in splicing efficiency upon insertion of a single or multiple nimtRNAs is position-dependent and is therefore comparable to effects observed for bona fide SREs.

### NimtRNAs increase alternative exon inclusion in a three-exon splicing reporter

Next, we wanted to investigate whether nimtRNAs are also able to increase exon inclusion in a three-exon splicing reporter exhibiting an alternatively spliced internal exon. The alternative splicing reporter (designated as Designer Exon) consisted of three exons of 126 bp, 82 bp, and 273 bp in length as well as intervening introns of 242 and 637 bp, placed downstream of a CMV promoter (Fig. 4d) [41]. The second exon was designed to contain an inefficient 3′-splice acceptor at the intron1/exon2-border, with its polypyrimidine tract composed of only 50% pyrimidines, thereby reducing inclusion of the alternative exon.

Upon introduction of nimtRNA^Tyr^ (Y) into the first intron, i.e., 88 bp downstream of the 5′-splice site, an increase in PSI (percent spliced in) levels from 27.0 ± 5.4 (wild type) to 54.1 ± 4.9 (with insertion of nimtRNA^Tyr^) could be observed (Fig. 4e). In contrast, introduction of nimtRNA^Tyr^ into the second intron 100 bp downstream of the alternative exon resulted in a decrease in PSI levels to 21.0 ± 5.1, while insertion into the middle of the intron (i.e., situated 280 bp up- and downstream from the exon borders) or close to the 3′-splice site (i.e., 100 bp upstream of the third exon) resulted in PSI levels of 24.7 ± 5.9 and 30.5 ± 6.8, respectively, comparable to the wt reporter construct lacking nimtRNAs (Fig. 4e).

As stated above, we postulate that nimtRNAs originated from mtRNAs, encoded within the mitochondrial genome. However, in the course of evolution, nuclear nimtRNAs have acquired specific mutations, compared to their mitochondrial ancestors. Thus, to determine whether the mitochondrial ancestors of nimtRNAs, i.e., bona fide mtRNAs, promoted splicing as observed for their nuclear-encoded counterparts, we also analyzed the influence of mtRNAs on alternative splicing. Hence, we introduced bona fide mouse mtRNA^Gln^, mtRNA^Ser1^, mtRNA^Ser2^ or the reverse-complementary variant of mtRNA^Ser1^, designated as mtRNA^Ser1r.-c.^, into intron 1 of the alternative splicing reporter. Indeed, mtRNA^Gln^, mtRNA^Ser1^, mtRNA^Ser2^, and mtRNA^Ser1r.-c.^ increased exon inclusion (Fig. 4f). Furthermore, the reverse-complementary mtRNA^Ser1r.-c.^ showed comparable effects on exon inclusion to a two-exon Low0-eGFP splicing reporter, harboring nimtRNA^Trp^ in reverse-complementary orientation.

In contrast, the reverse-complementary variant of mtRNA^Gln^, i.e., mtRNA^Gln r.-c.^, did not enhance exon inclusion (PSI = 25.1 ± 5.1) (Fig. 4f). Upon closer inspection, mtRNA^Gln^ contained one U-G base pair in each of its stem regions. As a consequence, the reverse-complementary variant mtRNA^Gln r.-c.^ exhibits an A-C pair at this position (Additional file 1: Fig. S4). Upon mutation of the respective nucleotides (i.e., A to G or C to U), we observed a partial rescue of alternative exon inclusion (PSI = 32.7 ± 5.1) (Fig. 4f, mtRNA^Gln r.-c. mut.^). Interestingly, three identical copies of the same nimtRNA, i.e., mtRNA^Ser2^, resulted in a further substantial increase in alternative exon inclusion (Fig. 4f), as was already observed in the two-exon Low0-eGFP splicing reporter employing multiple, but different, nimtRNAs (see above and Fig. 3c).

NimtRNAs of the same isotype might be derived from different mtRNA founder sequences since we hypothesized that nuclear integration of mtRNAs occurred at several different time points in evolution (see above). Importantly, sequences of nimtRNAs of the same isotype can be influenced to different extents by evolutionary pressure, and thus may differ extensively in their capacity to influence splicing.

Thus, to determine potential differences in the splicing capacity of a single nimtRNA isotype, we investigated different variants of nimtRNA^Ser2^ in the alternative splicing reporter. To this end, all nimtRNA^Ser2^ sequences were aligned by MUSCLE [42] and distances between sequences were estimated by employing MEGA to calculate the maximum likelihood [43]. This analysis resulted in the generation of distinct clusters, from which six candidate nimtRNA^Ser2^ sequences were chosen for splicing analysis in the alternative splicing reporter. In comparison, effects of bona fide human mtRNA^Ser2^ on splicing were also investigated. Interestingly, in the course of these analyses, we observed different stimulatory effects of nimtRNA^Ser2^ variants on alternative exon inclusion, ranging from 59.7 to 90.9 in PSI (Fig. 4g); thereby, bona fide human mtRNA^Ser2^ resulted in a PSI of 87.8. Thus, different nimtRNAs, derived from the same isotype, as well as bona fide mtRNAs, can exert a wide range on exon inclusion within the alternative splicing reporter.

### CRISPR/Cas9 mediated partial deletion of endogenous nimtRNA^Lys^ within intron 28 of the *PPFIBP1* gene results in decreased downstream exon 29 inclusion

**Fig. 5:**
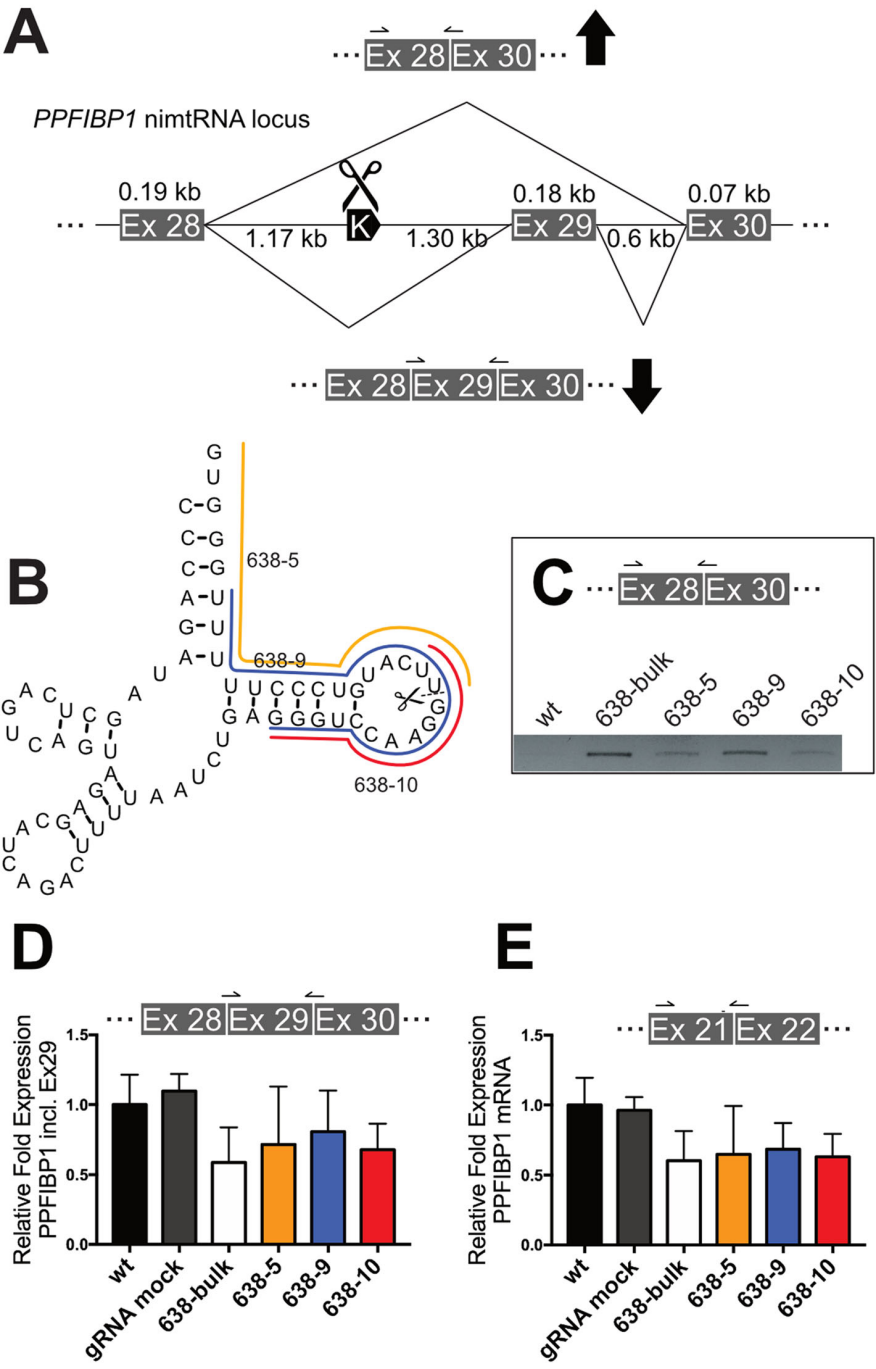
A CRISPR-mediated partial deletion of a nimtRNA downregulates downstream exon inclusion of an endogenous host gene. **a** The nimtRNA^Lys^ gene, located within intron 28 of the human *PPFIBP1* gene, was targeted by a CRISPR-mediated approach to analyze the influence of partial deletions within the nimtRNA^Lys^ gene on PPFIBP1 exon 29 inclusion. **b** Single clones of CRISPR-targeted cells were cultured and analyzed by Sanger sequencing for nimtRNA^Lys^ deletions; based on this analysis, three single clones, designated as 638-5, 638-9, and 638-10, indicated in orange, blue, and red, respectively, were selected. **c** Subsequently, by RT-PCR analysis comparing wt to bulk and single clone cells, respectively, the abundance of the PPFIBP1 mRNA transcript lacking exon 29 was assessed, employing primers as indicated. **d** The abundance of PPFIBP1 mRNA transcript harboring exon 29 was determined by RT-qPCR in wt cells and compared to bulk as well as single clone CRISPR-targeted cells employing primers as indicated. Cells targeted by a guideRNA not binding to the *PPFIBP1* gene (gRNA mock) were employed as an additional control. Normalization was performed to GAPDH. **e** In addition to the specific inclusion of exon 29, the general abundance of PPFIBP1 mRNA levels was determined by employing primers binding to exon 21 and 22 upstream of the nimtRNA locus, as indicated. Cells targeted by a guideRNA not binding to the *PPFIBP1* gene (gRNA mock) were employed as an additional control. Normalization was performed to GAPDH. Averages and standard deviations were determined from three independent sets of experiments

To analyze the influence of endogenous nimtRNAs on host gene splicing, we targeted nimtRNA^Lys^ located within intron 28 of the *PPFIBP1* gene by a CRISPR/Cas9-based approach (Fig. 5a). The *PPFIBP1* gene comprises 31 exons and contains a single intronic nimtRNA^Lys^, exhibiting a canonical tRNA-like secondary structure. *PPFIBP1* encodes for the PPFIA binding protein 1 (PPFIBP1), a member of the LAR protein-tyrosine phosphatase-interacting protein family, also designated as liprins and is abundantly expressed in HEK 293T cells. By employing the CRISPOR web tool [44], a sgRNA (single guide RNA) was designed to directly target the T-loop of nimtRNA^Lys^ and was cloned into lentiCRISPRv2 for lentiviral transduction of HEK 293T cells, as described previously [45]. Subsequently, the efficiency of nimtRNA indel formation was confirmed by TIDE analysis [46] (Additional file 1: Fig. S5). We thereby determined an editing efficiency of 94.5%, where approximately 31% of cells harbored deletions between 13 and 17 nts, respectively.

From the bulk of nimtRNA^Lys^-targeted cells, we screened for single-cell clones in order to obtain defined and extended CRISPR-induced nimtRNA^Lys^ deletions. Indeed, through these analyses, we identified three clones, which displayed different larger deletions within the nimtRNA^Lys^ gene, comprising either the T-arm (i.e., 638–10) or the T-arm and the acceptor stem (i.e., 638–5 and 638–9), and exhibiting deletions from 12 to 20 and 25 nt, respectively (Fig. 5b).

By comparing wt cells to bulk or single clones of nimtRNA^Lys^-targeted cells, similar to our constitutive splicing reporter assay, we analyzed the *PPFIBP1* mRNA for inclusion of exon 29, located downstream of nimtRNA^Lys^, by employing primers spanning exon/exon borders (Fig. 5c, d). In the course of these analyses, we detected a splicing variant in bulk or single clones of nimtRNA^Lys^-targeted cells which lacked exon 29, while this variant was absent in wt cells (Fig. 5c).

As for the constitutive splicing reporter (see above), in bulk nimtRNA^Lys^-targeted cells, a decrease of about 41% in exon 29 inclusion compared to untreated wt cells was observed. In addition, we found a decrease in exon 29 inclusion for all three single clones, compared to wt cells, ranging from 19 to 32% respectively (Fig. 5d).

As an additional control, we investigated *PPFIBP1* exon 29 inclusion in cells with a nimtRNA^Lys^ unrelated sgRNA, targeting an intronic region of the *SYTL4* gene (designated as gRNA mock, Fig. 5d), which resulted in the same levels of exon 29 inclusion as observed for wt cells. The range of standard deviations of exon 29 inclusion levels in nimtRNA^Lys^-targeted cells might thereby potentially be due to the influence of cellular stress and/or differences in cell confluency in these cells. Likely, cellular stress or varying cell confluences might result, for example, in a high variability in the expression of *trans*-acting protein factors, associated with nimtRNA-mediated splicing increase. Consistent with this hypothesis, we noted the influence of these parameters also in previous transient transfection experiments for our constitutive or alternative splicing reporter assays.

Within the *PPFIBP1* gene, exon 29 is annotated as a constitutive exon and might be essential for proper gene function. By employing a different set of primers, targeting exon 21 and 22 upstream of the nimtRNA locus, we observed a general downregulation of spliced *PPFIBP1* mRNA levels in nimtRNA^Lys^-targeted cells (Fig. 5e). Again, gRNA mock-treated cells exhibited identical levels of *PPFIBP1* mRNA levels as wt cells. These findings are consistent with CRISPR-induced deletions within the intronic nimtRNA^Lys^ gene decreasing *PPFIBP1* host gene levels by inhibiting exon 29 inclusion.

### Splicing-associated proteins bind to a nimtRNA transcript

Previous reports have demonstrated that splicing is modulated by *trans*-acting proteins which bind to SREs located within pre-mRNA transcripts [2]. Interestingly, computational analysis of the nimtRNA^Tyr^ sequence revealed a negative HEXplorer score [40] indicating the presence of potential hnRNP and/or hnRNP-like binding sites (Additional file 1: Fig. S6). Thus, to elucidate which nuclear proteins might bind to nimtRNAs, resulting in the splicing upregulation/exon inclusion, we performed an RNA immunoprecipitation (RIP) assay. This approach utilized a biotinylated T7 transcript containing five nimtRNAs identical to those employed previously (i.e., YCNAW, see above) which was incubated with a nuclear extract generated from HEK 293T cells. We propose that mouse and human nimtRNAs are likely to be recognized by identical *trans*-factors in HEK 293T cells, since we showed that nimtRNA structure, rather than sequence, is responsible for observed effects.

**Fig. 6:**
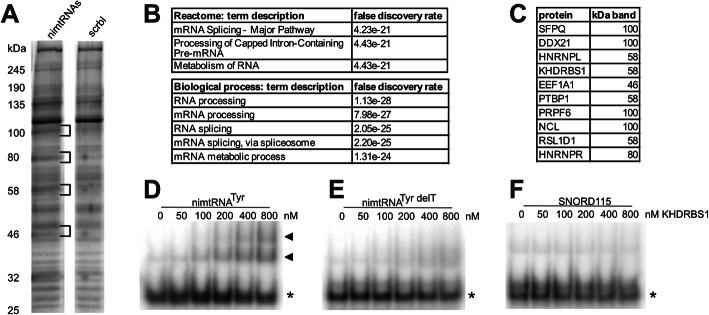
NimtRNA protein interaction analysis. **a** Biotinylated transcripts containing or lacking nimtRNAs were incubated with nuclear protein extracts. Subsequently, binding proteins were isolated and analyzed by PAGE; differential band patterns were excised and analyzed by MS. **b** GO analysis of nimtRNA transcript interacting proteins. **c** The top 10 nimtRNA transcript interacting proteins ranked by absolute abundance. **d**–**f** An electrophoretic mobility shift assay was performed with increasing concentrations of KHDRBS1 being incubated with a transcript of the nimtRNA^Tyr^ (**d**), nimtRNA^Tyr delT^ (**e**), or snoRNA SNORD115 (**f**). The unbound transcript is indicated by an asterisk; the transcript-protein complexes are indicated by triangles

Employing streptavidin beads, proteins associated with nimtRNAs were isolated and subsequently separated by SDS-PAGE (Fig. 6a). Bands which predominantly appeared in the nimtRNA pull-down approach, but not in the control lacking nimtRNAs (designated as scrbl), were excised from the gel and subsequently analyzed by LC-MS. By a STRING protein-protein interaction network analysis of proteins identified by MS, we determined a highly significant network of proteins being bound to the nimtRNA transcript (*p* < 1.0e−16). An inherent GO analysis determined “RNA processing” and “RNA splicing” as the biological processes, and “mRNA Splicing-Major Pathway” in the Reactome, enriched significantly (Fig. 6b). The top 10 most abundant nimtRNA transcript interacting proteins are listed in Fig. 6c.

Consistent with our experimental analyses, we observed that binding sites of 24 proteins which function in splicing or exhibit other regulatory roles (see Additional File 6: Table S5 for a complete list) overlap with nimtRNA sequences, either in HepG2 or K652 cell lines, or both, as determined by analysis of ENCODE eCLIP data. Of these, G3BP1 and NSUN2 have a more than 2-fold enrichment of their binding sites in nimtRNAs. Notably, KHDRBS1 was also found to be enriched for nimtRNA binding in this dataset as observed in MS analysis by our pull-down experiments (see above).

Previously, Marnef et al. have demonstrated that canonical mtRNA^Thr^, but not other mtRNAs, interact with PTBP1 in the cytosol [47]. In contrast to KHDRBS1, we could not retrieve overlaps of PTBP1 binding sites with nimtRNA loci in our analysis (see below). Hence, by an EMSA assay, we also investigated a potential direct interaction of PTBP1 and nimtRNA^Tyr^. To this end, a transcript of nimtRNA^Tyr^ including an extra 10 nucleotides at the 5′ and 3′ terminus, in order to not resemble processed tRNA ends, was radioactively labeled and incubated with increasing concentrations of PTBP1 protein. However, we did not observe specific binding of PTBP1 to nimtRNA^Tyr^, which is consistent with reported eCLIP data which provided no evidence of PTBP1 being directly associated with nimtRNAs.

KHDRBS1, also designated as Sam68, was found by MS analysis among the ten most abundant proteins binding to the nimtRNA-containing transcript. Thereby, KHDRBS1 belongs to the STAR (signal transduction and activation of RNA metabolism) protein family and has previously been demonstrated to be associated with several roles in mRNA metabolism, including splice site selection.

We thus also employed the nimtRNA^Tyr^-containing transcript in an EMSA analysis by adding increasing amounts of KHDRBS1 protein to the labeled nimtRNA transcript. Indeed, increasing concentrations of KHDRBS1 protein resulted in a mobility shift of the nimtRNA^Tyr^ transcript (Fig. 6d). Interestingly, in this analysis, two distinct bands were observed in the EMSA analysis employing KHDRBS1. As a negative control, the T-arm deletion mutant of nimtRNA^Tyr^ (designated as nimtRNA^Tyr delT^), due to its reduced potential to increase splicing efficiency (see above), was employed (Fig. 6e). We observed a significantly reduced binding of KHDRBS1 to the nimtRNA^Tyr delT^ mutant transcript compared to the nimtRNA^Tyr^ transcript. As a second negative control, the brain-specific snoRNA SNORD115 was employed in the EMSA assay, but was found to be deficient in binding to KHDRBS1, consistent with KHDRBS1 binding specifically to canonical sequences and/or structural features of nimtRNAs (Fig. 6f).

## Discussion

Despite the lack of canonical nimtRNA processing, in particular their intronic location, the partial conservation of their structure, and their association with splicing-related proteins prompted us to investigate the potential role(s) of nimtRNAs in pre-mRNA splicing. By employing well-characterized splicing reporter constructs [3, 41], harboring nimtRNAs within their introns, we could indeed demonstrate that nimtRNAs increase splice site recognition, as previously reported for bona fide intronic splicing regulatory elements, designated as SREs. SREs bind *trans*-acting protein factors, which interact with spliceosomal components during different steps of spliceosomal assembly [48]. Hence, SREs are able to increase splicing of constitutive introns and also promote the inclusion of alternative exons (see below), either by affecting 5′- or 3′-splice site choice [48].

A potential reason for nimtRNAs having remained unidentified for also harboring SREs might be that previous studies on SREs have mainly focused on mini-gene, in vivo pull-down or in vitro SELEX approaches which neglected intron-located RNA secondary structure elements as major determinants for recognition by protein *trans*-factors (splicing regulatory proteins; SRPs) [49–53]. In addition, in silico RNA secondary or higher-order structure prediction of SREs results in many false-positive structures, as has been shown by the lack of their experimental validation [54].

However, previously some pre-mRNA secondary structures have indeed been demonstrated to significantly affect splicing efficiencies [55] even though SRP binding sites are generally single-stranded [56]. As an example, intron 7 of the *SMN* gene, implicated in the development of spinal muscular atrophy, harbors an intronic SRE element with an intricate RNA secondary structure in addition to specific sequence discriminators [57]. NimtRNAs, functioning as intronic SREs, might thus exhibit a combination of specific sequence elements and structural motifs that are required for proper *trans*-factor recruitment [57]. As has been observed for SREs, we demonstrate that nimtRNAs, located within introns of host genes, are able to increase host gene pre-mRNA splicing *in cis.* In agreement with these findings, *cis*-acting intronic snoRNAs and miRNAs have also been demonstrated to be implicated in regulation of pre-mRNA splicing [5].

By placing nimtRNAs at different positions within the intron of the two-exon splicing reporter, we demonstrate that the nimtRNA-mediated increase in splicing is position-dependent, as was previously reported for a number of SR and hnRNP binding sites [3]. Notably, when placed close to the 5′-splice site but distant from the respective 3′-splice site, nimtRNA^Tyr^ exhibited the most pronounced effect on splicing efficiency in the two-exon splicing reporter in agreement with the predicted negative integral of the nimtRNA^Tyr^ sequence by the HEXplorer profile (Additional file 1: Fig. S4).

In addition to multiple nimtRNAs, also single nimtRNAs increased pre-mRNA splicing efficiency to different extents when located within the intron of the eGFP two-exon splicing reporter. When several nimtRNAs were inserted, an increase in host gene splicing efficiency was positively correlated with the number of intronic nimtRNAs. These findings are consistent with a function of nimtRNAs resembling SREs, which have been shown to cooperate in splicing of a single intron by binding simultaneously to several *trans*-acting splicing factors [49].

Splice site selection is generally regulated by spliceosomal snRNP proteins and auxiliary factors. Thereby, the intrinsic 5′-splice site strength is mainly defined by the complementarity of the 5′-splice site to the U1 snRNA. It has been reported that introns harboring weak splice sites, thereby resulting in a low basic level of splicing, are more subject to splicing regulation by splicing regulatory elements [58, 59], which is corroborated by this study. Upon employing a reporter construct with a weak 5′-splice site, we observed a more pronounced increase in nimtRNA-mediated splicing. The presence of nimtRNAs increased mRNA abundance by about 13-fold for the weak reporter, compared to an increase of approximately 3-fold for the more efficient splicing reporter construct. One explanation for the observed increase in host gene mRNA abundance might be a nimtRNA-mediated increase in host gene transcription. However, we were able to demonstrate that pre-mRNA levels remain largely unchanged when nimtRNAs are placed within the intron of the efficient two-exon splicing reporter, whereas spliced mRNA levels increased significantly (Fig. 3f).

In addition to the Low0-eGFP splicing reporter employed in the experiments described above, we also investigated the effects of nimtRNAs on a three-exon splicing reporter. The internal exon thereby exhibited an inefficient 3′-splice acceptor at the intron1/exon2-border, thus reducing efficient inclusion of the alternative exon in this reporter (Fig. 4d) [41]. NimtRNA^Tyr^ increased exon inclusion when located in the upstream intron, whereas it decreased exon inclusion when located in the downstream intron close to the 5′-splice site (Fig. 4e). The three-exon splicing reporter employed in our experiments contained a weak 3′-splice site. This resulted in impaired alternative exon inclusion due to inefficient 3′-splice site recognition by the U2 auxiliary factors 1 and 2 (U2AF1 and U2AF2). We show that in this splicing reporter, nimtRNAs are able to significantly increase downstream alternative exon inclusion likely as a result of enhancing 3′-splice site recognition.

To determine sequence or structural requirements for the observed splicing increase within nimtRNAs, we mutated structural domains and sequence motifs within nimtRNA^Tyr^ and introduced these mutated variants into our two-exon splicing reporter assay. In this context, deletions of either tRNA arm, i.e., D-arm, T-arm, anticodon arm, and the acceptor stem of nimtRNA^Tyr^ reduced splicing efficiency to various extents, compared to the non-mutated form of nimtRNA^Tyr^. These findings are consistent with the requirement of the conservation of canonical tRNA secondary structure for the function of nimtRNAs (see above). Future experiments will attempt to address the question whether in addition specific sequence motifs within nimtRNAs are also required and to what extent the structural requirements are different for nimtRNAs deriving from different mtRNAs. The differences between nimtRNAs and their consensus structures at least hint to some variability.

In addition to nimtRNAs, we also analyzed the effects of bona fide human mtRNAs in the two- and the three-exon splicing reporters (see above). In most cases, nimtRNAs only carry few mutations compared to their mitochondrial tRNA counterparts. As described above, most mutations are found in loop regions or as compensatory base changes in the stem regions of the nimtRNAs. In the three-exon splicing reporter, mtRNA^Ser2^, lacking the entire D-arm, increased alternative exon inclusion. Also, we observed that different isoforms of nimtRNA^Ser2^, displaying various base changes compared to human bona fide mtRNA^Ser2^, promoted alternative exon inclusion to different extents. It is thus tempting to speculate that by changing only a small number of bases within nimtRNAs, the ratio of mRNA splice-isoforms can be modulated.

Surprisingly, we also observed an increase in reported splicing when employing a nimtRNA^Trp^ variant, which is present in the reverse-complementary orientation relative to its host gene. Since bona fide mitochondrial tRNAs are encoded on both strands of the mitochondrial genome both of which are transcribed, several nimtRNAs are present in reverse-complementary orientation within their host genes. Thus, by introducing a single nimtRNA^Tyr^ in a reverse-complementary orientation into the eGFP splicing reporter construct, we observed a comparable stimulatory effect on splicing efficiency as we observed for their canonical counterparts present in the sense-orientation. A plausible explanation for these observations might be that reverse-complementary variants of nimtRNAs also display a canonical cloverleaf structure due to maintaining stem-loop structures (Additional file 1: Fig. S4).

The fact that nimtRNA cloverleaf structures might be a main determinant for the observed splicing effects is in line with results obtained employing mtRNA^Gln^ in sense or antisense orientation in the three-exon splicing reporter. We observed that sense mtRNA^Gln^ increased alternative exon inclusion while in contrast this was not found for antisense mtRNA^Gln r.-c^. We thereby noted that mtRNA^Gln^ exhibits single G-U wobble base pairs within each of its stem regions. Hence, the antisense variant of mtRNA^Gln^ displays A-C pairs in its corresponding stem regions which might potentially interfere with canonical tRNA cloverleaf formation. Indeed, following mutation of the respective nucleotides, i.e., by introducing compensatory base changes (i.e., changing an A to a G or a C to a U), we could partially rescue the effect on alternative exon inclusion. Partial rescue of exon inclusion might, in addition, depend on (short) sequence motifs within nimtRNAs not present in mtRNA^Gln r.-c^. It is noteworthy that in the mitochondrial genome both mtDNA strands are transcribed, resulting in the generation of polycistronic transcripts that also contain reverse-complementary variants of mtRNAs. These reverse-complementary mtRNA variants have indeed been suggested to fulfill non-canonical functions within mitochondria [60–62].

Utilizing a pull-down assay followed by MS analysis, we identified specific nuclear proteins which showed high binding affinity to a nimtRNA transcript. These nuclear proteins included Splicing factor, proline- and glutamine-rich (SFPQ), heterogeneous nuclear ribonucleoprotein L (HNRNPL), KH RNA-binding domain containing signal transduction associated 1 (KHDRBS1, also designated as Sam68), and polypyrimidine tract-binding protein 1 (PTBP1), respectively. Since all these proteins are involved in pre-mRNA splicing, we envision that these proteins may form a nimtRNA-associated pre-mRNA splicing network. Whether or not this network is identical in mouse and humans remains to be investigated in the future. In general, splicing machineries are similar and splicing-associated diseases have been successfully explored and treated in different mouse models, including the Spinraza® (Nusinersen) antisense oligonucleotide targeting the intronic splicing silencer ISS-N1 [63]. Furthermore, nimtRNAs can be found in mouse and several other species [33] suggesting a potential common pathway.

The results of the MS analysis are consistent with our analyses of eCLIP data from the ENCODE project which showed a significant enrichment in binding sites for 10 splicing-related genes (24 in total), most notably KHDRBS1, pointing towards a functional involvement of nimtRNAs in regulating splicing efficiency and specificity. Since eCLIP data were derived from two different cell lines (HepG2 and K562), we would not expect a perfect match with our experimental MS data since splicing patterns, and thus splicing regulation, differs substantially between different cell types (see below).

In agreement with our MS analysis, in a gel retardation assay (EMSA), we were able to detect specific binding of KHDRBS1, but not PTBP1 to nimtRNA^Tyr^. By applying increasing concentrations of KHDRBS1 protein, we observed two distinct band shifts for nimtRNA^Tyr^ consistent with formation of dimers of KHDRBS1, which is in line with previous reports [64]. In contrast, the T-arm deletion mutant exhibited a significant decrease in its affinity to KHDRBS1. The low, but specific affinity of KHDRBS1 for a nimtRNA might indicate that KHDRBS1 is required but not sufficient for nimtRNA-mediated effects on splicing and that additional proteins may also contribute to this process, as also corroborated by our MS analysis.

It is of note that KHDRBS1 has been associated with positive and negative 3′- and 5′-splice site selection as well as with polypyrimidine tract binding [65]. It has been reported to directly interact with U2AF2, which in turn has been shown to associate with both the branchpoint-binding protein SF1 and the 3′-splice site-binding protein U2AF1 and is thus involved in the regulation of splicing. Indeed, the three-exon splicing reporter, employed in our analyses, harbors a weak 3′-splice site at the 5′ terminus of the alternative exon. Accordingly, an increased inclusion of the alternative exon was observed in the presence of a nimtRNA within the upstream intron.

Similarly, nimtRNAs also increased splicing efficiency in a constitutively spliced intron, since the Low0- and Low2-eGFP splicing reporters also possess a weak 3′-splice site due to a short pyrimidine-rich region and a shorter than canonical spacer between branchpoint sequence and 3′-splice site. Hence, KHDRBS1 might play a role in the repression of canonical 3′-splice site recognition in these reporters, by impairing 3′-splice site recognition as previously suggested [66, 67]. This notion is corroborated by our results showing that a more efficient 3′-splice site reduces the potential impact of nimtRNAs on splicing in the constitutive splicing reporter (Additional file 1: Fig. S7).

Based on our experiments employing two- and three-exon splicing reporters, respectively, we propose that nimtRNAs located within introns of their cognate host genes are able to affect host gene splicing patterns. Using a CRISPR/Cas9-based approach, we were able to show for the first time that partial deletions of a single endogenous nimtRNA^Lys^ gene within intron 28 of the *PPFIBP1* gene are able to significantly decrease downstream exon 29 inclusion. The induced deletions from 12 to 25 nt in length were located within the T-arm or the T-arm and the acceptor stem of nimtRNA^Lys^, respectively, which we also show to be essential for nimtRNA^Tyr^-mediated splicing effects in our two-exon splicing reporter assay.

CRISPR-targeting of nimtRNA^Lys^ resulted in an increase in the abundance of *PPFIBP1* transcripts lacking exon 29 and a decrease in the abundance of transcripts including exon 29, located downstream from nimtRNA^Lys^, consistent with a role of nimtRNA^Lys^ in promoting *PPFIBP1* pre-mRNA splicing. Thereby, exon 29 is annotated as a constitutive exon, and thus likely to be essential for *PPFIBP1* protein function. This is corroborated by the observation that exclusion of exon 29 results in a general reduction of *PPFIBP1* mRNA levels (see above) consistent with nimtRNAs acting as ISEs which are known to regulate host gene mRNA levels. Our findings concerning an endogenous host gene, i.e., *PPFIBP1*, recapitulates the splicing effects observed in the two-exon constitutive splicing reporter assay described above, where we show that nimtRNAs increase eGFP reporter mRNA levels and hence eGFP protein synthesis.

## Conclusions

By employing splicing reporter constructs as well as investigating an endogenous host gene, our study demonstrates a potential novel function of nimtRNAs, present in introns of host genes in the human genome, in pre-mRNA splicing. Since processing of bona fide mitochondrial tRNAs within mitochondria has been shown to be directly linked to mitochondrial mRNA processing, it is thereby tempting to speculate that nimtRNAs might have acquired a related novel function in processing/splicing of nuclear-encoded pre-mRNAs. Future studies will have to focus on the involvement of nimtRNAs in splicing regulation within all 76 introns of their human host genes as well as on their interaction and regulation by *trans*-acting protein factors.

## Material and methods

### Cloning and reporters

Cloning of reporter constructs was either performed by classical cloning or PCR mutagenesis approaches. For nimtRNA integration, we digested the Low0/Low2-eGFP vector with KpnI and NdeI. NimtRNAs were amplified including 10–50 bp up- and downstream by PCR from mouse genomic DNA employing overhanging primers, cleaved with the respective enzyme(s) and ligated into the reporter vectors. The nucl. YCNAW construct was amplified by PCR with overhanging primers from a gene fragment and cloned into the Low0-eGFP reporter by KpnI and NdeI digestion. Mutations and several integrations of nimtRNAs were done by mutagenesis PCR employing the NEB Mutagenesis Kit (NEB). Oligonucleotides and gene fragments were ordered from IDT. Plasmids were transformed into TOP10 *E. coli* (One Shot® TOP10 Chemically Competent or Electrocomp™ *E. coli*), clones were selected, and DNA was extracted using NEB Miniprep Kit and sequenced by Eurofins. Positive clones were cultured; DNA for transfection experiments was extracted employing the Qiagen Midiprep Kit. Primer sequences are provided in the supplementary (Table 1 and 2).

**Table 1:** List of primers used for classical cloning in this study.

Plasmid	Primer fwd	Primer rev	Entry plasmid
Y	GGGGTACCCCGTTCCG ATATCTTTGTGATTG	GGAATTCCATATGGAA TTCCCACCTTAAGACCT CTGGTA	Low0-eGFP
YC	GGGGTACCCCGTTCCG ATATCTTTGTGATTG	GGAATTCCATATGGAA TTCCTCTACTTCTACCG CCGAAA	Low0-eGFP
YCN	GGGGTACCCCGTTCCG ATATCTTTGTGATTG	GGAATTCCATATGGAATTCCAGACCTCAACTAGATTGGC	Low0-eGFP
YCNA	GGGGTACCCCGTTCCG ATATCTTTGTGATTG	GGAATTCCATATGGAATTCCAACTTCTGATAAGGACTGTAG	Low0-eGFP
(nucl.) YCNAW	GGGGTACCCCGTTCCG ATATCTTTGTGATTG	GGAATTCCATATGGAATTCCGCTGTCATAAGTACAATAACC	Low0-eGFP/Low2-eGFP
C	GGGGTACCCCTTTTTACCAGAGGTCTTAAGG	GGAATTCCATATGGAA TTCCTCTACTTCTACCG CCGAAA	Low0-eGFP
N	GGGGTACCCCCTACCGCCATTTTTTTTTTCG	GGAATTCCATATGGAA TTCCAGACCTCAACTAG A TTGGC	Low0-eGFP
A	GGGGTACCCCGCCAATCTAGTTGAGGTCT	GGAATTCCATATGGAATTCCAACTTCTGATAAGGACTGTAG	Low0-eGFP
W	GGGGTACCCCCTACAGTCCTTATCAGAAGTT	GGAATTCCATATGGAATTCCGCTGTCATAAGTACAATAACC	Low0-eGFP

**Table 2:** List of primers used for mutagenesis PCR cloning in this study

Plasmid	Primer fwd	Primer rev	Template plasmid
Y delD	GTCGAATTGCAAATTCGAAG	CTTAAGACCTCTGGTAAAAAG	Y
Y delT	TAAGACTTCTACCGCCAT	ACCTTCGAATTTGCAATTC	Y
Y delAcc	ttcgaaggtgtagagaaatctctacCTACCGCCATTTTTTTTTTC	tttgcaattcgacatgaatatcacctTCTGGTAAAAAGGGGTAC	Y
Y delAnti	AGGTGTAGAGAAATCTCTAC	CATGAATATCACCTTAAGAC	Y
Y scrbl	agtatttgcggattaacaatgactggtaccactGAGGTCTTAAGGTGGGAATTC	agcattaaggctttaacactcttggttttattaaTATGTTCATTAATCGTTGATTATTCTC	Y
Y r.-c.	tttacagtctaatgcttactcagccattttaccGAGGTCTTAAGGTGGGAATTC	tctaaacacagaggtttaaatcctctttttaccaTATGTTCATTAATCGTTGATTATTCTC	Y
C r.-c.	tttgcaattcgacatgaatatcaccttaagaccCTACCGCCATTTTTTTTTTC	ttcgaaggtgtagagaaatctctactaagacttTCTGGTAAAAAGGGGTAC	C
mt T	TAAACCGGAGATGAAAACCTTTTTCCAAGGACAGAGGTCTTAAGGTGGGAATTC	CAAGACTGGTGTATTAGTTTATACTACAAGGACTATGTTCATTAATCGTTGATTATTCTC	Y
Y25	ctaaacacagaggtttaaatcctctttttaccaCAGTGGCAATGAGAGTGAAG	atttacagtctaatgcttactcagccattttaccTCTTCGGAGAGCTTAAGG	Low0-eGFP
Y50	ctaaacacagaggtttaaatcctctttttaccaGTATCAGCACTTGTGGAG	atttacagtctaatgcttactcagccattttaccTTCTCCTTCACTCTCATTG	Low0-eGFP
Y100	ctaaacacagaggtttaaatcctctttttaccaGGATATTGATGATCTGTAGTGCTACAG	atttacagtctaatgcttactcagccattttaccCAAGGAGCATGGTGCCCC	Low0-eGFP
Y214	ctaaacacagaggtttaaatcctctttttaccaCAAGAAGTAGTATTGGTAAATGTGACAGAAAATTTTAAC	atttacagtctaatgcttactcagccattttaccTGGGTTGGGGTCTGTGGG	Low0-eGFP
Y657	ctaaacacagaggtttaaatcctctttttaccaGAGCCAATTCCCATACATTATTG	atttacagtctaatgcttactcagccattttaccAAAGGATACCTTTGGACAG	Low0-eGFP
Y1100	ctaaacacagaggtttaaatcctctttttaccaAATAATCTTTAAGCAATCCTC	atttacagtctaatgcttactcagccattttaccGTTTTATTATTTCCAAATTGTTCTC	Low0-eGFP
Y1450	ctaaacacagaggtttaaatcctctttttaccaTGAGGGACAATTGGAGAAGTG	atttacagtctaatgcttactcagccattttaccTATCGCCTCCTCCAGGTC	Low0-eGFP
Y2125	ctaaacacagaggtttaaatcctctttttaccaTATTCACCATTATCGTTTCAG	atttacagtctaatgcttactcagccattttaccTCCCTGCCTAACTCTATTC	Low0-eGFP
Y2150	ctaaacacagaggtttaaatcctctttttaccaCGTTTCAGACCCACCTCC	atttacagtctaatgcttactcagccattttaccATAATGGTGAATATCCCTGCC	Low0-eGFP
i1-Y	ctaaacacagaggtttaaatcctctttttaccaAGTTTCCCAAAATTTTATTTTTGG	atttacagtctaatgcttactcagccattttaccAAAACAGGCTTCCAACAATG	Designer exon
i2–5′-Y	ctaaacacagaggtttaaatcctctttttaccaAACTATAAGGTAGACATTCTTATTC	atttacagtctaatgcttactcagccattttaccAGCTATTAAAAATATTGTTAATGATTC	Designer exon
i2-c-Y	ctaaacacagaggtttaaatcctctttttaccaTCATTTTCATTTCCAGGG	atttacagtctaatgcttactcagccattttaccTAACTGTGCTCAAATTCTAG	Designer exon
i2–3′-Y	ctaaacacagaggtttaaatcctctttttaccaCTGTGCACACGAGTGTAG	atttacagtctaatgcttactcagccattttaccCAAGAAAGAGAGATAACTGGG	Designer exon
mt Ser2 hs	cccccatgtctaacaacatggctttctcaAGTTTCCCAAAATTTTATTTTTGG	catgagttagcagttcttgtgagctttctcAAAACAGGCTTCCAACAATG	Designer exon
nimtRNA Ser2–12	catatattaatataacaatatggctttatcaAGTTTCCCAAAATTTTATTTTTGG	ggggcattattagcagttatcgcatactttctAAAACAGGCTTCCAACAATG	Designer exon
nimtRNA Ser2–32	ccttttgtgtatcatccataacttttctaAGTTTCCCAAAATTTTATTTTTGG	aaaagaatgagcagttttttgttttgtttAAAACAGGCTTCCAACAATG	Designer exon
nimtRNA Ser2–34	gggggtggcagtctctttcatcAGTTTCCCAAAATTTTATTTTTGG	atgttaaaaacatggcttcatcAAAACAGGCTTCCAACAATG	Designer exon
nimtRNA Ser2–55	cccccagaaaccaaactggctctcttgAGTTTCCCAAAATTTTATTTTTGG	tcgtggggttaggtcctcatgcttctctAAAACAGGCTTCCAACAATG	Designer exon
nimtRNA Ser2–56	tgtcaaatgtattagtttattctttcAGTTTCCCAAAATTTTATTTTTGG	tcaaaatttaaaacttttgctctttcAAAACAGGCTTCCAACAATG	Designer exon
nimtRNA Ser2–67	ttaacaagaaaggctttttcaAGTTTCCCAAAATTTTATTTTTGG	tcaaagtaggcttttctttttAAAACAGGCTTCCAACAATG	Designer exon

### Cells, cell culture, and manipulation

Cell culture experiments were performed with HEK 293T (ATCC® CRL-3216™) and Flip-In™-293 (Invitrogen, #R75007) cells. Cells were cultured in 4.5 g/l glucose and l-glutamine DMEM medium (Gibco) with 100 units/ml penicillin, 100 units/ml streptomycin (Gibco), and 10% heat-inactivated FBS (Gibco) at 37 °C, saturated humidity, and 5% CO_2_. Cells were transiently transfected by lipotransfection employing Metafectene (Biontex). A total of 500,000 cells were seeded 24 h prior to transfection in a 6-well dish. 1.5-μg plasmids, i.e., 1 μg of splicing reporter and 0.5 μg DsredExpress2 transfection control, were transfected employing 5 μl Metafectene. Stable transfections were performed by employing the Flip-In system in Flip-In™-293 cells. Low0-eGFP splicing reporters were cloned into an EF1α promoter containing pcDNA5/FRT-derived expression vector. In total, 375 ng of plasmid was co-transfected with 1.125 ng of pOG44 into Flip-In™-293 cells and selected by Hygromycin resistance, as previously described (Invitrogen).

### RNA isolation, reverse transcription, and (q)PCR analysis

Total RNA of cells was isolated 24 h post transfection with TRI reagent (Sigma) following the manufacturer’s protocol. In total, 500 ng of total RNA of cells was employed for DNA digestion and subsequent reverse transcription utilizing the SuperScript IV VILO with ezDNase Kit following the manufacturer’s protocol. Complete DNase digest was assessed by quantitative PCR. For quantitative PCR analysis, 2 μl of 1:100 diluted cDNA in a 6-μl sample volume with Luna Dye (NEB) was employed according to the manufacturer’s protocol. The primers used are listed in Table 3. Quantification was performed employing the ∆∆Ct method, normalizing to a co-transfected plasmid containing the DsredExpress2 reporter gene. The respective unaltered reporter construct was employed as reference. Means and standard deviations of the RQ values of at least three individual experiments were calculated. Significance was assessed by one-way ANOVA in GraphPad prism 5 (GraphPad Software, San Diego, CA). RT-PCR analysis of alternative splicing reporters was performed by using 2 μl of 1:100 diluted cDNA in a 20 μl PCR reaction with Pfu Polymerase and primers binding to the first and the last exon (listed in Table 3).

**Table 3:** List of primers used for RT-(q)PCR analysis

Target	Primer fwd	Primer rev
eGFP mRNA	TGAGGAGGCTTTTTTGGAGG	TTCACTAATCGAATGGATCTGTC
eGFP pre-mRNA	GTAATACGACTCACTATAGGGC	CATCAATATCCCAAGGAGCATG
Beta-Actin	CGTCACCAACTGGGACGACA	CTTCTCGCGGTTGGCCTTGG
DsredExpress2	GTCCTTCCCCGAGGGC	TTCAGCACGCCGTCGCG
GAPDH	CCATGGGGAAGGTGAAGGTC	AGTTAAAAGCAGCCCTGGTGA
Alt. spl. Rep.	AGTGATTCAGAACCGTCAAG	TCCACCACCGTCTTCTTTAG
PPFIBP1 incl ex 29	ccaaagtgaagCCAAAGAAACTT	aatcttccatctgctctaaccg
PPFIBP1 excl ex 29	gttctagagcctcgttttaacg	tgaatcttccatcttcactttgg
PPFIBP1 upstream	gaaacagaaaaagagacagcaga	CTTCTCCTAAGTtttccaaagagt

### Northern blot analysis

For detection of mRNAs, 10 μg of total RNA was loaded onto a 2% Agarose gel with 2.2 M formaldehyde and subsequently blotted onto Amersham™ Hybond™-N Membranes (Thermo Fisher). The RNA was UV-crosslinked to the membranes at 0.12 kJ using a UV crosslinker (Stratagene, La Jolla, USA). Respective mRNA transcripts were detected with DIG-labeled probes amplified by PCR. For detection of ncRNAs, 10 μg of total RNA was separated on denaturing polyacrylamide gels (8 or 12%, acrylamid:bisacrylamid ≙ 29:1, 7 M urea, 1× TBE) at 150 to 250 V for 3 to 4 h. Subsequently, the gel was stained with ethidium bromide for 10 min. RNA was transferred on Amersham™ Hybond™-N Membranes (GE Healthcare) employing the Trans-Blot SD Semi-Dry Transfer Cell (Bio-Rad, Vienna, Austria) at 400 mA for 45 min. The RNA was UV-crosslinked to the membranes at 0.12 kJ using a UV crosslinker (Stratagene, La Jolla, USA). Respective transcripts were detected with radioactively labeled oligonucleotides.

### Fluorescence measurement

HEK 293T cells were seeded at 40,000 cells per well in a 96-well flat-bottom plate (Greiner) and transfected the next day. Fluorescence was measured in live HEK 293T cells 48 h post transfection in a Clariostar Microplate Reader (BMG Labtech). eGFP was excited at 485 ± 10 nm, dichroic filter at 503 nm, and measured at 525 ± 15 nm with the gain set to 1170. DsredExpress2 was excited at 554 ± 10 nm, dichroic filter at 571.2 nm, and measured at 591 ± 15 with the gain set to 1460. Focal height was determined at 5.2 from top. Scan mode was set to spiral with a scan diameter of 6 mm and 50 flashes per well. Measured values of PBS-transfected cells were subtracted from transfected cells. Ratio of eGFP and Dsredexpress2 was calculated. Mean and standard deviation was calculated from five separate experiments. Statistical significance was determined by one-way ANOVA in GraphPad prism 5 (GraphPad Software, San Diego, CA).

### CRISPR/Cas9 targeting of nimtRNAs

The experimental setup for CRISPR/Cas9 targeting of the endogenous nimtRNA within the gene *PPFIBP1* was designed using CRISPOR [44]. The respective nimtRNA sequence including 50 bp up- and downstream was analyzed using the online tool in order to generate candidate guideRNAs. Only guideRNA sequences directly targeting the respective nimtRNA and without off-targets for 0, 1, or 2 mismatches were considered. The respective primers for gRNA cloning into the lentiCRISPR v2 by Zhang [45] were ordered, following the CRISPOR workflow. Cloning was performed following the protocol by Zhang [45]. Viruses were produced by transfecting HEK 293T cells in a 6-well dish with 400 ng of the respective gRNA construct, 200 ng pSPAX2, and 200 ng VSVg with 5 μl Metafectene following the manufacturer’s protocol. Supernatant of the transfected cells was taken after 48 and 72 h for transduction of HEK 293T target cells. Cells were grown and selected with puromycin for 2–3 weeks. Single clones were generated by seeding 0.2–0.5 cells in 96-well plates in DMEM medium supplemented with 10% FBS, 1% pen/strep, and 1% methylcellulose.

### Electrophoretic mobility shift assay

Electrophoretic mobility shift assays were performed as described in [68]. Briefly, T7 transcripts were generated from PCR amplified templates overnight. Transcripts were dephosphorylated by Calf Intestinal Alkaline Phosphatase and subsequently 5′ labeled with [γ-^32^P]-ATP. In total, 100 fmol of radioactively labeled transcript was incubated with heparin for 1 h at 4 °C and separated by PAGE at 4 °C at 100 V on a native 1× TBE 5% polyacrylamide gel (75:1 Acrylamid:Bisacrylamide).

### Biotin-streptavidin pull-down

Transcripts were generated employing the HiScribe™ T7 High Yield RNA Synthesis Kit (NEB, Frankfurt, Germany). Transcripts were biotinylated employing the Pierce™ RNA 3′ End Biotinylation Kit (NEB, Frankfurt, Germany) according to the manufacturer’s protocol. Briefly, 50 pmol of transcript was labeled by ligation with a single biotinylated nucleotide at the 3′-terminus and subsequently purified. Labeling efficiency of biotinylated RNA was determined by dot blotting whilst following the description of the Pierce™ Chemiluminescent Nucleic Acid Detection Module Kit (Thermo Scientific, Vienna, Austria).

Proteins binding to the respective transcripts were isolated employing streptavidin magnetic beads (Thermo Scientific, Vienna, Austria) according to the manufacturer’s protocol. Briefly, beads were washed and supplemented with RNA Capture buffer. Then, 50 pmol biotin-labeled RNA was added to the beads, followed by an incubation for 30 min at RT with agitation. Protein-RNA binding buffer (Tris pH 7.5 20 mM, NaCl 50 mM, MgCl_2_ 2 mM, Tween 0.1%(v/v)), 30% glycerol, and 20 μg of nuclear lysate were added to the beads and incubated for 60 min at 4 °C with agitation. RNA-binding protein complexes were collected and washed with wash buffer (50 mM NaH_2_PO_4_, 300 mM NaCl, 20 mM imidazole) and eluted in 50 mM ammonium acetate. Proteins were separated by SDS-PAGE and subsequently silver stained.

### Mass spectrometry

Silver-stained gel bands were excised from SDS-PAGE gels, reduced with dithiothreitol, alkylated with iodoacetamide, and digested with trypsin (Promega) as previously described [69]. Tryptic digests were analyzed using an UltiMate 3000 RSCLnano-HPLC system coupled to a Q Exactive HF mass spectrometer (both Thermo Scientific, Bremen, Germany) equipped with a Nanospray Flex ionization source. The peptides were separated on a homemade fritless fused-silica micro-capillary column (100 μm i.d. × 280 μm o.d. × 20 cm length) packed with 2.4 μm reversed-phase C18 material. Solvents for HPLC were 0.1% formic acid (solvent A) and 0.1% formic acid in 85% acetonitrile (solvent B). The gradient profile was as follows: 0–4 min, 4% B; 4–57 min, 4–35% B; 57–62 min, 35–100% B; and 62–67 min, 100% B. The flow rate was 300 nl/min.

The Q Exactive HF mass spectrometer was operating in the data-dependent mode selecting the top 20 most abundant isotope patterns with charge > 1 from the survey scan with an isolation window of 1.6 mass-to-charge ratio (m/z). Survey full-scan MS spectra were acquired from 300 to 1750 m/z at a resolution of 60,000 with a maximum injection time (IT) of 120 ms, and automatic gain control (AGC) target 1e6. The selected isotope patterns were fragmented by higher-energy collisional dissociation with normalized collision energy of 28 at a resolution of 30,000 with a maximum IT of 120 ms, and AGC target 5e5.

Data analysis was performed using Proteome Discoverer 2.2 (Thermo Scientific) with search engine Sequest. The raw files were searched against the uniprot *Homo sapiens* database. Precursor and fragment mass tolerance was set to 10 ppm and 0.02 Da, respectively, and up to two missed cleavages were allowed. Carbamidomethylation of cysteine was set as static modification and oxidation of methionine as variable modification. Acetylation, methionine loss, and methionine loss plus acetylation were set as N-terminal dynamic modification of proteins. Peptide identifications were filtered at 1% false discovery rate. Only proteins identified by at least 2 unique peptides were considered for subsequent analyses. The STRING online tool was used to analyze the proteins thus identified in terms of protein-protein interaction and Gene Ontology. Textmining, experiments, databases, co-expression, neighborhood, gene fusion, and co-occurrence were chosen as active interaction sources. Confidence was set to medium (0.400).

### Protein expression and purification

Expression and purification of human recombinant PTBP1 was performed as described in [47]. The expression construct was kindly provided by Douglas Black (University of California, Los Angeles, US). Human recombinant KHDRBS1 (Sam68; CAT#: TP300263) was ordered from Origene (Rockville, Maryland, USA).

### Search for genomic loci of nimtRNA genes

Since there is no tool to accurately annotate MTLs, we tested different annotation strategies on genomic sequences of human and mouse. Annotations were tested either on the nuclear numtDNA sequences reported in Tsuji et al. [25] or the entire nuclear genome as reference. We refer to the two strategies as *NUMT*-based and *genome*-based, respectively. To detect tRNAs, we used the tRNA annotation tool tRNAscan-SE v2.0 [36] in a modified manner, applying the integrated mtRNA search mode (-M option) not to mitochondrial genomes, but to nuclear sequences. Regardless of whether the default (20 bits) or a very low (0–20 bits) cutoff score was used for filtering hits, the same results were returned. In an alternative approach, we applied Infernal v1.1.2 [35] as search engine with specific covariance models (CMs) for each of the 22 mtRNA families taken from MiTFi [18]. These CMs contain information on aberrant mtRNAs in addition to the normal mtRNA sequence and structure consensus which can help to detect MTLs exposed to high selection pressure. All Infernal hits were retained to find also MTLs that are not well conserved. Since we ran Infernal separately with each of the 22 CMs, we obtained overlapping predictions. For each locus, the MTL hit with the highest score was retained. To determine the transcriptional context, e.g., intronic, exonic, and intergenic, we assigned transcript annotations to the MTLs. We defined MTLs as intergenic if they could not be assigned to an annotated transcript. All MTLs located in introns are annotated as nimtRNAs.

### Performance evaluation

For each numtDNA, the original mitochondrial sequence is traceable. We therefore can reconstruct the number, types, and order of the mtRNA copies expected within each numtDNA. This synteny information is used to validate the direct MTL annotations obtained with the different analysis strategies. We count each hit as TP if the hit is located inside a numtDNA following the occurrence and order of the given synteny information. Some numtDNAs were copied from mitochondrial sequences that lack mtRNAs. Thus, we counted hits within such numtDNAs as FPs. Hits obtained outside from numtDNAs were also counted as FPs, since we thus far only have evidence for the existence of MTLs within recognizable numtDNAs only they are considered to be true MTLs.

### Sequence and structure consensus predictions

Multiple sequence and structure alignments were performed for each type of nimtRNA separately applying LocARNA [70]. The secondary structure predictions of tRNAscan-SE were used as constraints for the alignments.

### Measurement of evolutionary conservation

PhyloP (phylogenetic *P* value) scores were assigned to each sequence which has been predicted from multiple genome alignments of mammals. PhyloP scores are available from UCSC [71] and can be used to detect nucleotide substitution rates that are faster or slower than expected under neutral drift in genomic sequences of different species. However, testing the conservation of (parts of) a numtDNA is not trivial. While using PhyloP scores, one has to take into account that numtDNA, due to their quasi-repetitive nature, may have incurred problems in the genome assemblies and/or may be misaligned. Therefore, a complementary approach that compared the numtDNA to the extant human mitochondrial genome sequence was applied. The observed sequence divergence is in this case a sum of two independent effects: (i) the evolution of the numtDNA since its insertion and (ii) the evolution of mitochondrial genome since the insertion event. It can be expected that the selection pressure on the mitochondrial genome has remained neutral over time t0 because its functionality has been preserved. Since tRNAs are among the most stringently conserved genetic elements, the mitochondrial substitution rate of mtRNAs is smaller than the substitution rate of the mitochondrial proteins. Therefore, the evolutionary distance dt between MTLs or nimtRNA and mtRNA is expected dt = (sn + st)t0, while for the numtDNA it is dp = (sn + sp)t0, where sn is the neutral substitution rate in the mitochondrial genome. The substitution rates for MTLs or nimtRNA and numtDNA are given by st and sp, respectively. Outliers of this linear regression with unexpectedly large values, dp – dt, are then identified as the MTLs or nimtRNAs that have evolved slower than expected, i.e., those that have become subject to stabilizing selection after their insertion into the nuclear genome. Thus, the difference, dp – dt, is expected to be a linear function of t0. Since we are not able to calculate substitution rates and t0, we linearly transformed the model with sn + sp. The linear transformation leads to a model enabling MTLs or nimtRNAs to be obtained as outliers that are subject to a stronger selection pressure relative to numtDNA. Therefore, the sequence divergences can be used as measurement for the evolutionary sequence conservation. The sequence divergences (Hamming distance) dt and dp were computed by dividing their edit distance to the primordial mitochondrial sequence by their length. The edit distances were obtained by mapping the sequences to the mitochondrial genome. For this purpose, segemehl v0.2.0-418 [72] was applied with a low accuracy of 50%, while seeds with two differences were searched for to enable the mapping of strongly degraded sequences. Cook’s distance [73] was applied for the outlier test and was performed in R v3.6.0 using the stats package [74]. An observation with Cook’s distance larger than three times the mean Cook’s distance was considered to be an outlier. Only numtDNA sequences which are longer than 50 nts were used within this analysis to avoid overestimating shorter sequences.

### Determining RBP binding sites of nimtRNAs

To investigate the potential regulatory role of nimtRNAs by interaction with RNA-binding proteins (RBPs), their genomic loci with a list of experimentally validated RBP binding sites were intersected. The latter is readily available from the GENCODE project [75], which hosts a repository for BED files containing binding sites of a large set of RBPs derived from eCLIP experiments. These binding sites have already been quality controlled and show enrichment after normalization against IgG background; for more information on data generation and processing, please refer to https://www.encodeproject.org/eclip/. The genomic coordinates of nimtRNAs were intersected with RBP binding sites on the same strand to derive a list of overlaps by applying the BEDtools suite v2.29.0 [76]. RBPs that bound to each type of nimtRNA were then annotated according to their biological function with information derived from the GeneCards database [77]. The expected coverage of RBP per nucleotide intron was calculated from intersection of the eCLIP dataset with intron annotation (ENSEMBL biomart, hg38, version 98, [78]) for each RBP in the collection. By comparing this to the RBP coverage of binding sites in nimtRNA, the relative enrichment of RBP binding events in nimtRNAs over background could be calculated.

### Data sources

Mitochondrial and nuclear genomes of *Homo sapiens* (assembly hg38) and *Mus musculus* (assembly mm10) were downloaded from NCBI, release 90 [79]. The annotation of numtDNAs was obtained from [25] for the older assemblies mm9 and hg19. The numtDNA coordinates were converted to the latest genome assemblies mm10 and hg38 for mouse and human, respectively, applying the UCSC Liftover utility [71]. PhyloP scores of the multiple alignments of 29 mammalian genomes to hg38 were downloaded from UCSC (http://hgdownload.soe.ucsc.edu/goldenPath/hg38/phyloP30way/). Transcript annotations were obtained from Ensemble release 96 [78]. RBP interaction sites were downloaded from the ENCODE [80, 81] eCLIP repository [82].

## Supplementary Information

**Additional file 1: Figure S1.** Bioinformatic approach analysis. Performance evaluation of different MTL annotation strategies. Analysis of conservation densities of MTLs and numtDNAs. **Figure S2.** Intronic nimtRNA and snoRNA processing. The processing of intronic, Pol II- or Pol III-transcribed, plasmid encoded ncRNAs as assessed by northern blot analysis. **Figure S3.** NimtRNA effects on splicing. NimtRNA-mediated splicing increase as assessed by fluorescence. Position-dependent effects of a cluster of nimtRNAs as assessed by RT-qPCR. **Figure S4.** Proposed secondary structures of reverse-complementary nimtRNAs. Proposed secondary structures of nimtRNAs and their reverse-complementary counterparts as determined by tRNAscan or manually. **Figure S5.** TIDE analysis of the CRISPR/Cas9-targeted nimtRNA locus within intron 28 of *PPFIBP1*. CRISPR-induced nimtRNAs deletions within the *PPFIBP1* gene assessed by TIDE analysis. **Figure S6.** HEXplorer profile of nimtRNA^Tyr^ (67 nt). The HEXplorer score of nimtRNA^Tyr^ as determined in silico. **Figure S7.** Intron characteristics affect nimtRNA-mediated splicing regulation. The efficiency of 3′ splice site recognition was increased by mutation to determine its impact on the nimtRNA-mediated splicing increase.

**Additional file 2: Table S1.** Hg38 scores. MTL and nimtRNA hits within the human genome.

**Additional file 3: Table S2.** Mm10 scores. MTL and nimtRNA hits within the mouse genome.

**Additional file 4: Table S3.** MTL and nimtRNA outliers. MTLs and nimtRNAs exhibiting a higher degree of conservation as measured by the Cook’s distance.

**Additional file 5: Table S4.** NimtRNA consensus. Consensus sequence and structure of the different nimtRNA types.

**Additional file 6: Table S5.** Enrichment RBP binding. Binding sites of proteins enriched in nimtRNA sequences.

**Additional file 7.** Northern Blots. Full, uncut northern blots.

**Additional file 8.** ENCODE sources. List of ENCODE sources used for nimtRNA eCLIP meta-analysis.

**Additional file 9.** Review history.

## Data Availability

The mass spectrometry proteomics data have been deposited to the ProteomeXchange Consortium (http://proteomecentral.proteomexchange.org) via the PRIDE partner repository [83] with the dataset identifier PXD022204 [84]. Experimental eCLIP data and metadata from ENCODE was downloaded via the command “xargs -L 1 curl -O -L < eCLIP_datasets.txt.” The input text file for this command is available as supplement to this manuscript (Additional file 8).
