# Supplementary material for: Intronic tRNAs of mitochondrial origin regulate constitutive and alternative splicing
Source: Genome Biol. 2020 Dec 8;21:299. doi: 10.1186/s13059-020-02199-6 (PMC7722341; doi:10.1186/s13059-020-02199-6)
Supplement: Supplementary file 1 — Additional file 1: Figure S1. Bioinformatic approach analysis. Performance evaluation of different MTL annotation strategies. Analysis of conservation densities of MTLs and numtDNAs. Figure S2. Intronic nimtRNA and snoRNA processing. The processing of intronic, Pol II- or Pol III-transcribed, plasmid encoded ncRNAs as assessed by northern blot analysis. Figure S3. NimtRNA effects on splicing. NimtRNA-mediated splicing increase as assessed by fluorescence. Position-dependent effects of a cluster of nimtRNAs as assessed by RT-qPCR. Figure S4. Proposed secondary structures of reverse-complementary nimtRNAs. Proposed secondary structures of nimtRNAs and their reverse-complementary counterparts as determined by tRNAscan or manually. Figure S5. TIDE analysis of the CRISPR/Cas9-targeted nimtRNA locus within intron 28 of PPFIBP1. CRISPR-induced nimtRNAs deletions within the PPFIBP1 gene assessed by TIDE analysis. Figure S6. HEXplorer profile of nimtRNATyr (67 nt). The HEXplorer score of nimtRNATyr as determined in silico. Figure S7. Intron characteristics affect nimtRNA-mediated splicing regulation. The efficiency of 3′ splice site recognition was increased by mutation to determine its impact on the nimtRNA-mediated splicing increase. [file 13059_2020_2199_MOESM1_ESM.docx]

**Additional File 1. Supplementary data**


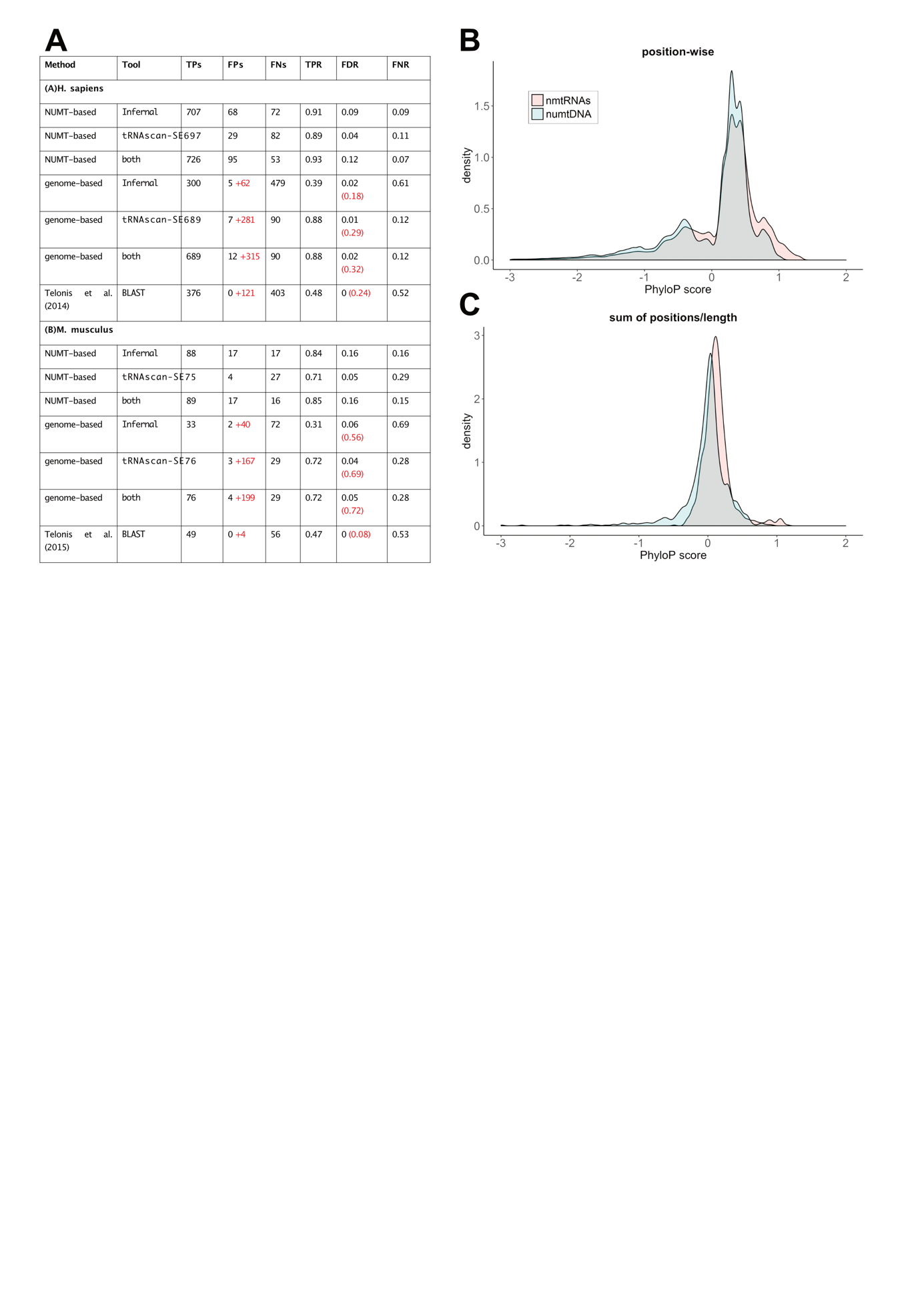


**Figure S1. Bioinformatic approach analysis.** (A) Performance evaluation of different MTL annotation strategies. Performance evaluation of annotated MTLs of (A) *H. sapiens* and (B) *M. musculus* are given for the *NUMT*-based and genome-based approach for the applied tools Infernal and tRNAscan-SE or the combination of both tools. Counts were calculated based on the synteny information given by the mitochondrial origin of the numtDNAs. The same validation was carried out with the data already published {Telonis, 2014 #1028;Telonis, 2015 #738}. MTLs are marked in red and are classified as potential FPs. In each approach, tRNAscan-SE shows the best balance between TPR and FDR. Although Infernal produced the highest count for TPs in the *NUMT*-based approach, the tool shows a reduced sensitivity in the *genome*-based approach. A combination of both increases the TPR, at the expense of FPs. Compared to currently published data, our implemented methods found > 1.8 times more TPs. (B) Conservation densities of MTLs and numtDNAs. Densities of conservation are shown for MTLs and the surrounding numtDNA sequences measured by PhyloP scores. The density of PhyloP scores for each single nucleotide of the sequences (A) and the normalized scores (sum of PhyloP scores per sequence divided by sequence length; B) are shown. Although most MTLs are not subject to negative selection, the PhyloP scores in MTLs are slightly enhanced compared to the surrounding numtDNA sequences.


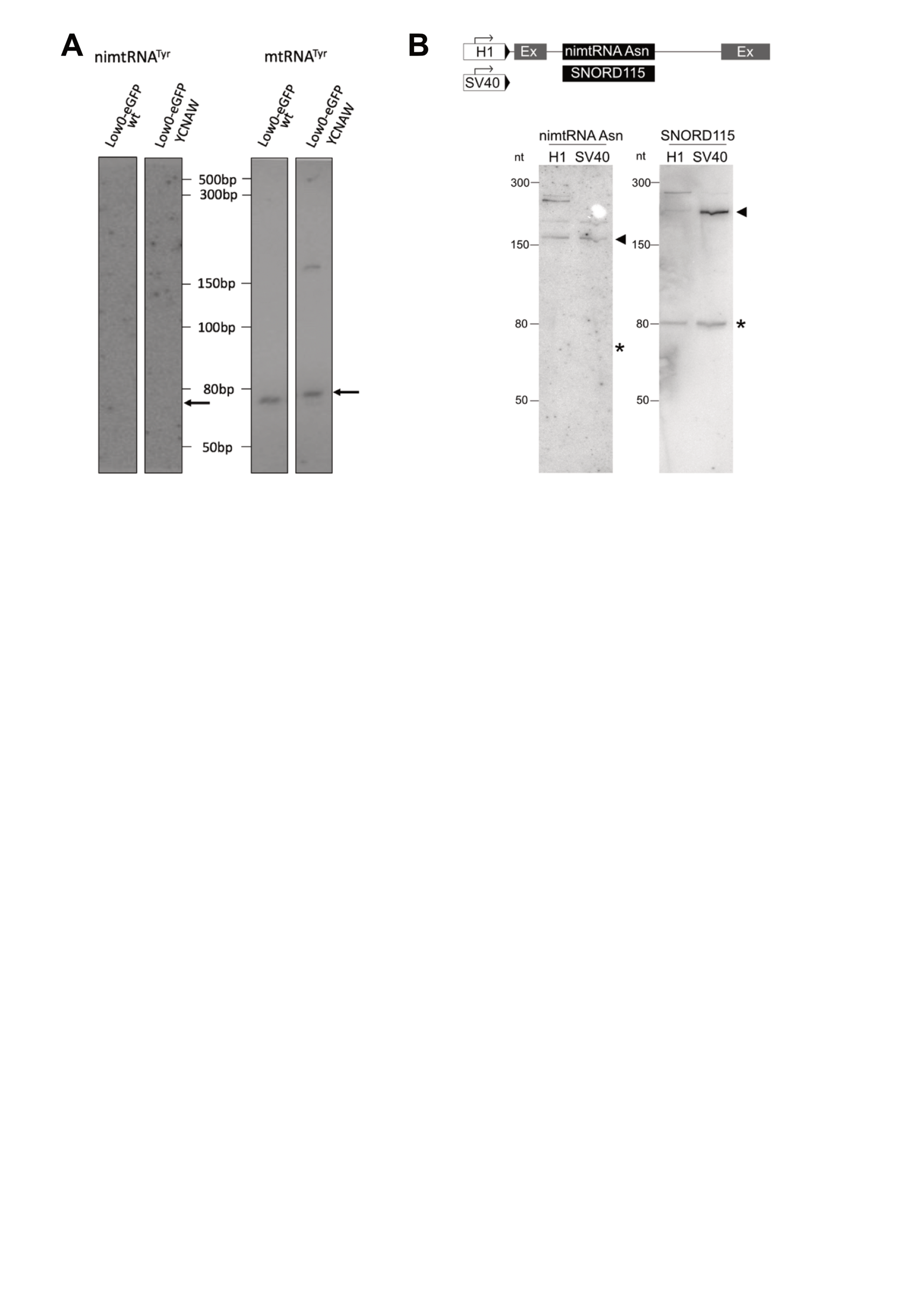


**Figure S2. Intronic nimtRNA and snoRNA processing.** (A) Northern blot analysis of nimtRNA processing from the intron of a constitutive eGFP splicing reporter. HEK 293T cells were transfected with either the reporter lacking (Low0-eGFP wt) or the reporter containing the stretch of five nimtRNAs from the mouse Myo3a gene (Low0-eGFP YCNAW). 24 hours later, RNA was extracted and employed for northern blot analysis by probing for nimtRNA^Tyr^, nimtRNA^Cys^ and nimtRNA^Asn^. Exemplarily, the probing for nimtRNA^Tyr^ is shown here (left). As a control for successful blotting procedure the canonical mitochondrial tRNA mtRNA^Tyr^ was also targeted by probing (right). Arrows indicate the height of the band expected for the respective processed target transcript. (B) NimtRNA processing from the intron of a Pol II and a Pol III reporter construct possessing an exon/intron/exon structure. Different ncRNAs, i.e. nimtRNA^Asn^ from the mouse Myo3a intronic locus and mouse snoRNA SNORD115, were integrated into the Low0 eGFP splicing reporter under the control of either a Pol III H1 promoter or the Pol II SV40 promoter. HEK 293T cells were transfected with these constructs and the respective ncRNA processing was determined by northern blot analysis. Asterisks mark the position at which the respective processed ncRNA is expected. Triangles indicate processing precursors.


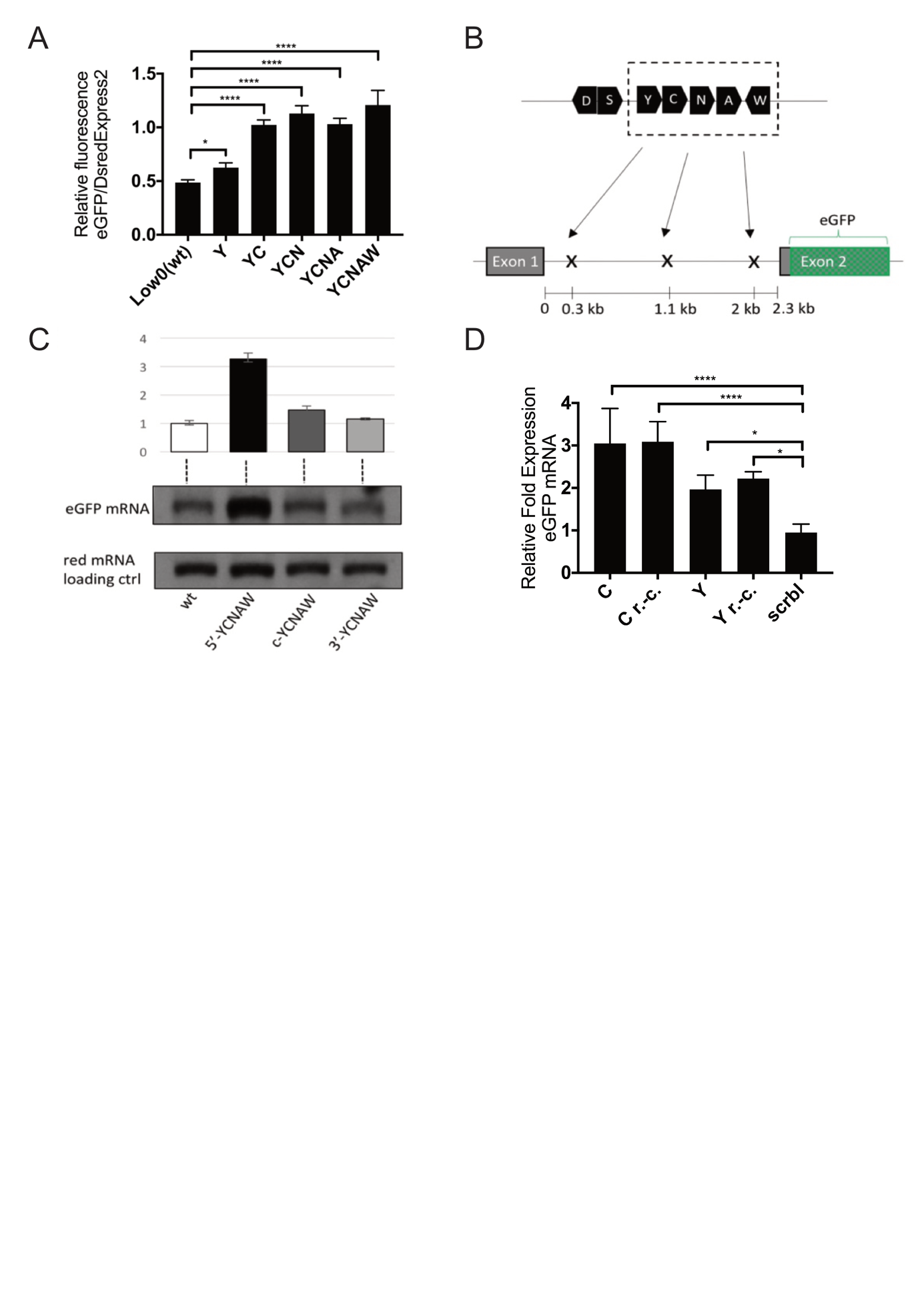


**Figure S3. NimtRNA effects on splicing.** (A) Protein measurement of cells transfected with constructs containing increasing numbers of intronic nimtRNAs. Fluorescence was measured in a plate reader 48 hours post transient transfection of Low0-eGFP splicing reporter, harboring either no intronic nimtRNA (Low0(wt)), a single nimtRNA^Tyr^ (Y), nimtRNA^Tyr^ and nimtRNA^Cys^ (YC), nimtRNA^Tyr^, nimtRNA^Cys^ and nimtRNA^Asn^ (YCN), nimtRNA^Tyr^, nimtRNA^Cys^, nimtRNA^Asn^ and nimtRNA^Ala^ (YCNA), or nimtRNA^Tyr^, nimtRNA^Cys^, nimtRNA^Asn^, nimtRNA^Ala^ and nimtRNA^Trp^ (YCNAW). Averages and standard deviations were determined from five independent sets of experiments. Error bars represent the SD and *, P < 0.05; ****, P < 0.0001 (ANOVA). (B)(C) Position-dependent effect of nimtRNAs on splicing efficiency/mRNA abundance. Two additional eGFP splicing reporter constructs were cloned by inserting the five nimtRNAs cluster into a central position (c-YCNAW) and into a 3’-splice site-close position (3’-YCNAW) within the intron of the Low0-eGFP splicing reporter. The previously employed YCNAW construct, with the nimtRNA cluster inserted close to the 5’-splice site (5’YCNAW), was transfected for comparison. Total RNA from cells transfected with the respective constructs was isolated and employed for northern blot and qPCR analysis (C). Normalization was performed on the co-transfected RFP (DsredExpress2).


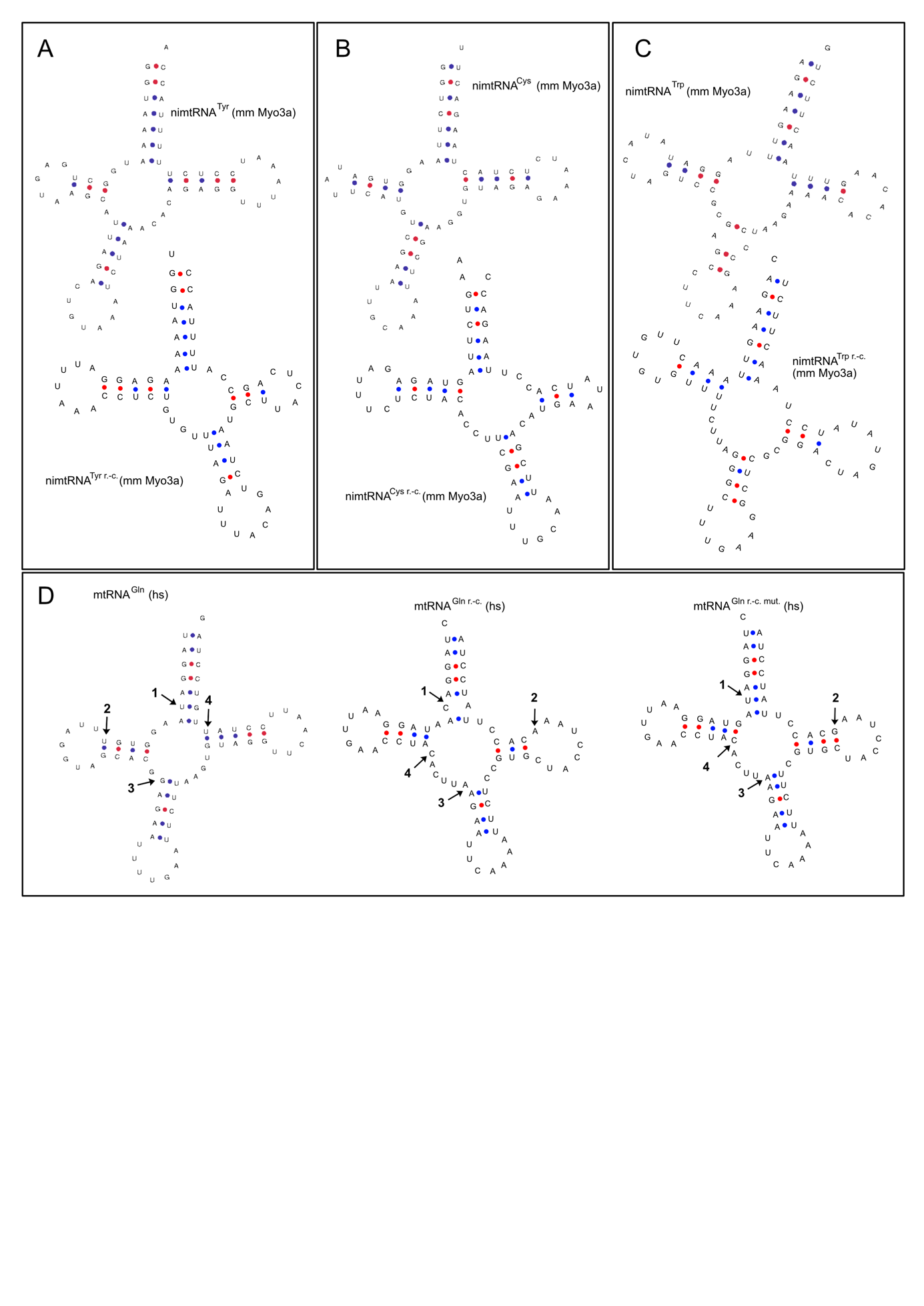


**Figure S4. Proposed secondary structures of reverse-complementary nimtRNAs.** Structure prediction of nimtRNAs in sense orientation i.e. nimtRNA^Tyr^, nimtRNA^Cys^ and nimtRNA^Trp^ as assessed by tRNAScan (top) and proposed secondary structure of reverse-complementary nimtRNAs (bottom). Structure prediction of mtRNA^Gln^ in sense, antisense and mutated antisense from left to right is indicated below. Arrows indicate base pairs perturbed in the antisense mtRNA.


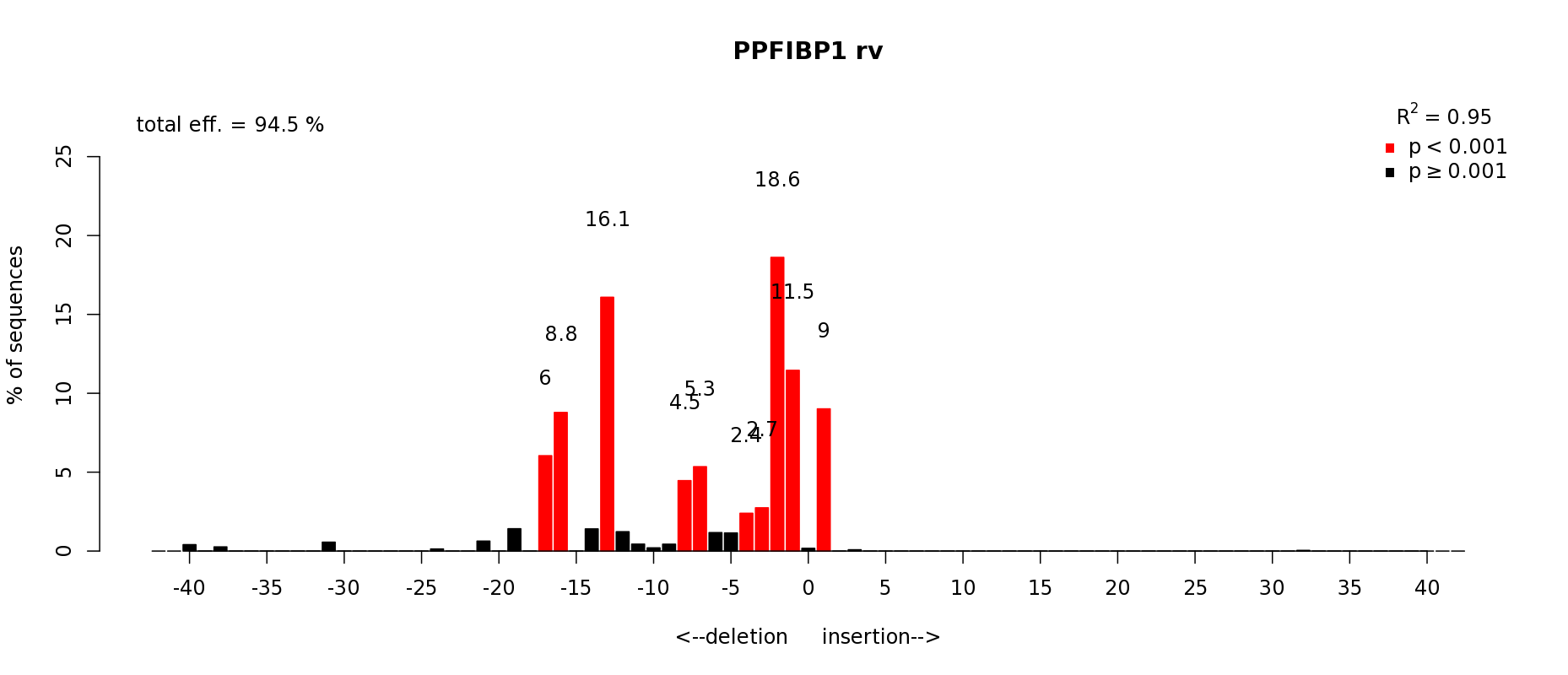


**Figure S5. TIDE analysis of the CRISPR/Cas9-targeted nimtRNA locus within intron 28 of PPFIBP1.** The bulk of PPFIBP1 nimtRNA^Lys^ CRISPR-target cells was sequenced for indel formations within the vicinity of the gRNA target site. Indel formation was assessed using the TIDE online tool.


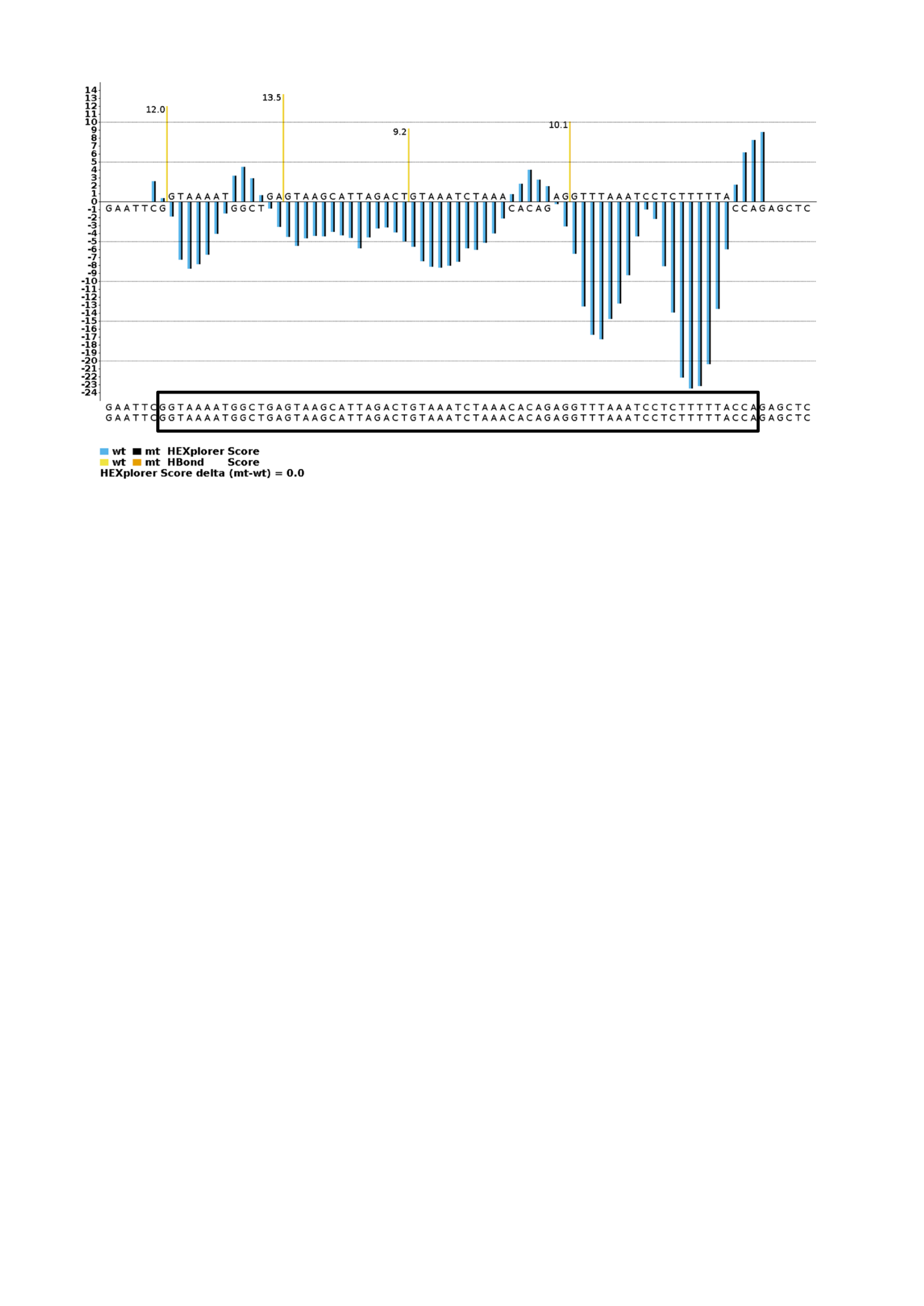


**Figure S6. HEXplorer profile of nimtRNA^Tyr^ (67nt).** The HEXplorer score per nt of this sequence is -5.0401, total integral is -337.69. Negative areas indicate putative hnRNP binding, yellow bars indicate HBond scores of GT dinucleotides.


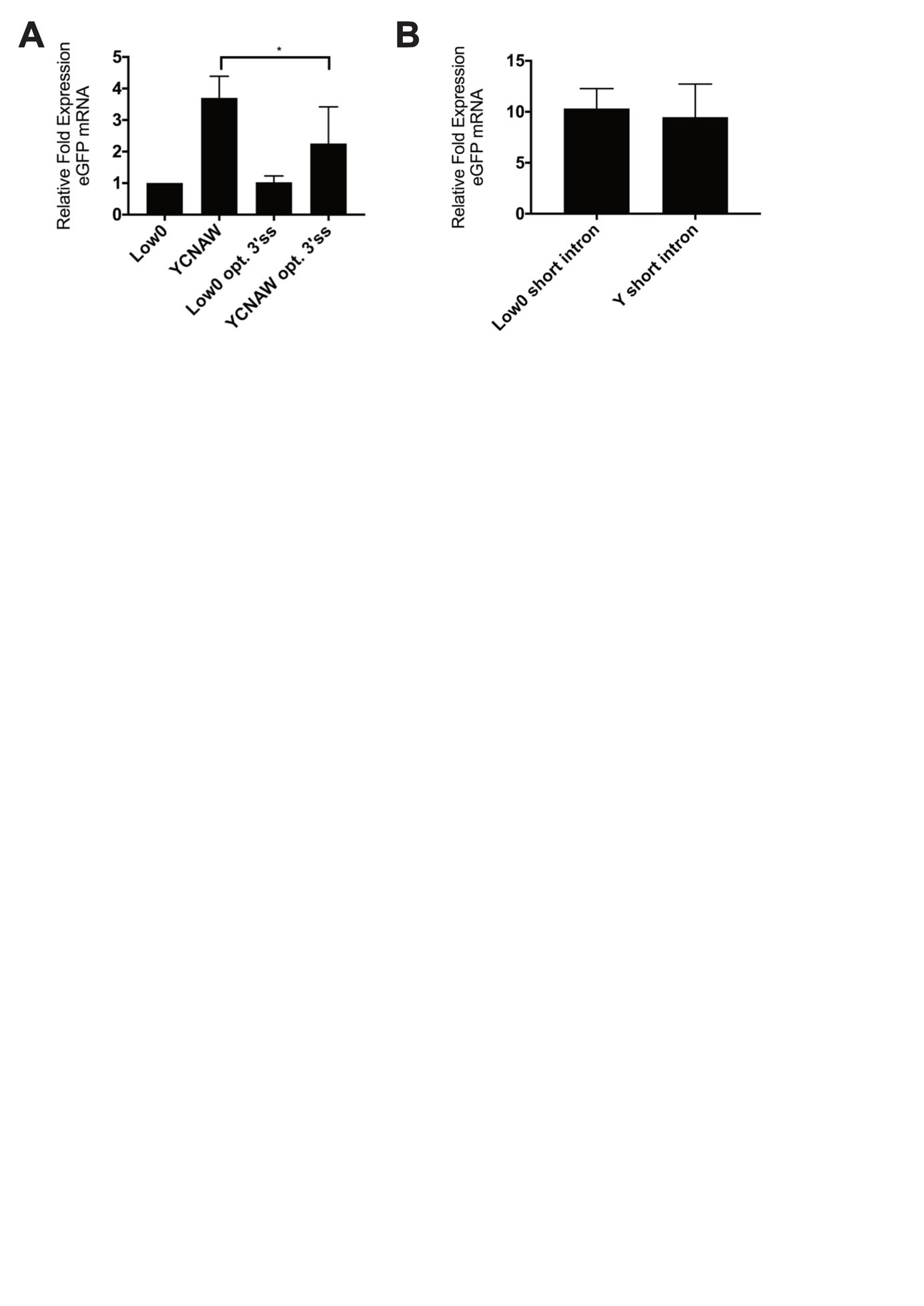


**Figure S7. Intron characteristics affect nimtRNA-mediated splicing regulation.** The Low0 constitutive splicing reporter construct lacking or containing the stretch of five nimtRNAs from the mouse Myo3a intronic locus was compared to the identical reporters harboring the more efficient 3’ splice sites from the ß-Globin gene intron4/exon5 border. Relative fold expression of reporter mRNA was determined by RT-qPCR. The Low wt splicing reporter was taken as reference. Error bars represent the SD and *, P < .05 (ANOVA).
