# Supplementary figures and images for: Intronic tRNAs of mitochondrial origin regulate constitutive and alternative splicing
Source: Genome Biol. 2020 Dec 8;21:299. doi: 10.1186/s13059-020-02199-6 (PMC7722341; doi:10.1186/s13059-020-02199-6)

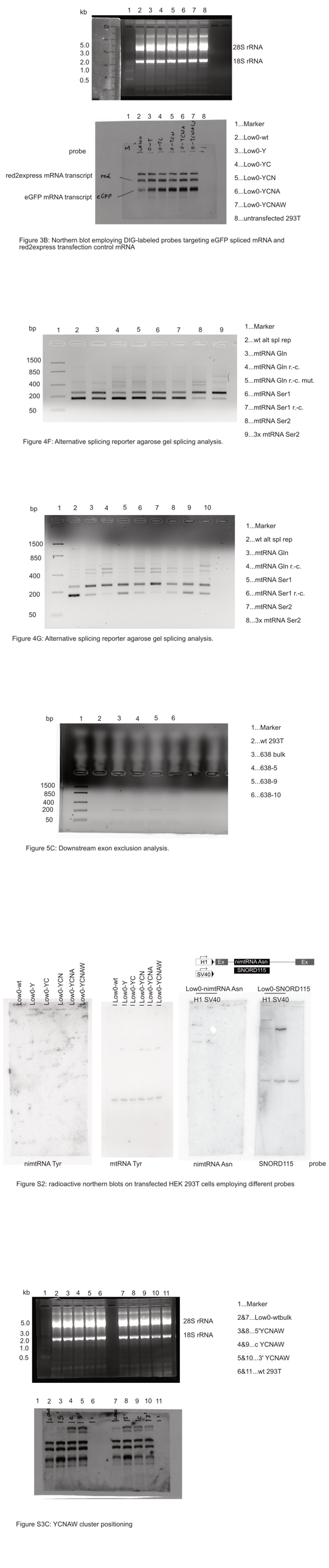

Supplement: Supplementary file 7 — Additional file 7. Northern Blots. Full, uncut northern blots. [file 13059_2020_2199_MOESM7_ESM.png]
