# Supplementary material for: Intronic tRNAs of mitochondrial origin regulate constitutive and alternative splicing
Source: Genome Biol. 2020 Dec 8;21:299. doi: 10.1186/s13059-020-02199-6 (PMC7722341; doi:10.1186/s13059-020-02199-6)
Supplement: Supplementary file 9 — Additional file 9. Review history. [file 13059_2020_2199_MOESM9_ESM.docx]

Review History

**First round of review**

**Reviewer 1**

**Are you able to assess all statistics in the manuscript, including the appropriateness of statistical tests used?** Yes, and I have assessed the statistics in my report.

**Comments to author:**

This manuscript explores a possible role for sequences that are embedded in the introns of genes and are similar to the sequences of mitochondrial tRNAs.

Major Concerns:

- A significant portion of the early part of the manuscript is devoted to discovering mitochondrial tRNA like sequences in the nuclear genome. The Introduction and the presentation of the manuscript are structured in a way that suggests that the authors are the first to examine the question of mitochondrial tRNA insertions in the nuclear genome. This is tantamount to reinventing the wheel as this very study was originally described in Telonis et al Frontiers in Genetics 2014. While the authors mention this earlier paper they do not appropriately describe its scope and goal which was to discover full-length tRNA-like sequences in the nuclear genome (and not nuclear insertions of mitochondrial DNA as the authors claim in the Introduction). Telonis et al labeled the sequences they discovered "tRNA-lookalikes" and the term has since been used in multiple papers. Even though the authors reference the paper, they ignore the earlier work and relabel the same sequences as "nuclear encoded mitochondrial tRNAs (nmtRNAs)". Instead the authors should use the original term "mitochondrial tRNA lookalikes" in order to put their work and new contributions in the proper context.

- On a related note, in the section "Conservation of nmtRNAs and nimtRNAs" the authors discuss sequence conservation. They report that they found several MT tRNA lookalikes "that appear to have evolved significantly more slowly than the adjacent numtDNA sequences." The authors are again attempting to reinvent the wheel. In both Telonis et al Frontiers Gen 2014 and Telonis et al RNA Biology 2015, both of which are referenced by the authors, there is explicit discussion about the fact that specific mitochondrial tRNA lookalikes are conserved identically in the nuclear genome.

- On line 223 of this manuscript, the authors also claim that "the insertion of nimtRNA genes in the respective introns of nuclear genes might be a very recent evolutionary event, which might have occurred independently in different species in addition to potential retainment of pre-existing nimtRNAs." This is not true. The Telonis et al 2015 paper showed that the concept of mitochondrial tRNA lookalikes is pervasive across at least 200 My of evolution and that in organisms from primates to marsupials one can find several mitochondrial tRNAs that have exact copies in their nuclear genomes.

- Many of the experiments that the authors describe involve a cluster of five mitochondrial tRNA lookalikes from intron 30 of Myo3a. In actuality there are seven such tRNAs and not five (the coordinates of all seven are in the manuscript's supplemental material). First, all seven of these mitochondrial tRNA lookalikes were reported in Telonis et al 2015 (see https://cm.jefferson.edu/data-tools-downloads/trna-lookalikes-beyond-homo-sapiens/), and are not new discoveries. Second, there is a methodological problem here as the authors arbitrarily dismiss two of the seven lookalikes. This is problematic in two ways. If the authors' hypothesis is that these intron-embedded lookalikes are functional then all seven sequences should be tested in the experiments and not only five. These seven tRNA lookalikes in intron 30 of Myo3a are in two groups: two tRNA lookalikes followed by a pseudo-copy of MT-CO1 followed by the other five tRNA lookalikes. This is the same arrangement one finds in the mitochondrial genome. If one searches the mouse genome with this mitochondrial segment (2 tRNAs plus MT-CO1 plus 5 tRNAs) one finds matches in the nuclear genome for both the full segment and the individual tRNAs. Many of these copies hit regions that do not contain any known transcripts and are exact copies of the mitochondrial tRNA. If the hypothesis put forth is correct shouldn't all of these lookalikes be in introns, either by themselves or as groups? I do not see how the hypothesis is warranted if there are comparable numbers of copies in intronic and in non-intronic regions of the genome.

Also, if the nuclear copies of this mitochondrial segment of 2 tRNAs plus MT-CO1 plus 5 tRNAs have a role in splicing in mouse, it is reasonable to assume that this is the case in the human genome as well. Here, a search reveals that the human orthologue of the mouse segment has several nearly full-length copies at different locations of the human nuclear genome that are not in the introns of any genes. This finding further questions the hypothesis that the mitochondrial tRNA lookalikes play a role in splicing.

- Starting on line 231, the authors make the claim that "NimtRNAs from nuclear introns are not processed as bona fide tRNAs" but do not present any evidence from RNA-seq data from the public domain to support the claim. In fact, Telonis et al Frontiers Genet 2014 presented evidence that the authors' claim is not true: using ENCODE data Telonis et al showed for multiple tRNA lookalikes that they are expressed and have the expected lengths. Telonis et al also showed that the expression of tRNA lookalikes is cell-type specific. More recently, Dror et al PLoS Genetics 2016 showed that tRNA expression in general appears to be both cell-type and time-specific. Therefore one cannot exclude that the absence of expression of these tRNAs in the (single) cell line and (single) time point that was used by the authors is not a manifestation of this specificity.

- The section that begins on line 259 describes a series of experiments involving microRNA miR-124. The authors claim that this microRNA has brain-specific expression and therefore is a good choice for use with HEK293 cells. This is incorrect because in humans miR-124 appears in multiple healthy and disease tissues. See for example: http://www.mirbase.org/cgi-bin/get_read.pl?acc=MI0000445 where it is shown to be present in skin, cervix, lung, tonsil, kidney (sample SRR553575 mentioned on the mirbase page), etc. The presence of miR-124 in human kidneys puts into question the appropriateness of using HEK293 cells for these experiments.

- I find the experiments in the section that starts on line 298 confusing. Since the authors weakened the strength of the 5´ splice site shouldn't they be obtaining less spliced product compared to the premRNA? Figure 3C says that they obtain more spliced product compared to the premRNA which I do not understand. Did the authors check the length of the spliced product after weakening the splice site to confirm that it did not contain any new segment from the 2.2kb intron?

- The experiments of the section that begins on line 321 use the corresponding tRNA lookalike sequences out of context. To convincingly show the role of these insertions the authors should introduce a sequence that is as long as the mitochondrial genome segment that spans the seven-lookalike cluster and scramble six of the seven lookalikes each time leaving the seventh one untouched. However, even if successful, the experiments would not address the role of the exact mitochondrial tRNA lookalikes that are found outside introns.

- The description of the LC-MS experiments suggests that there was only a single replicate. Given that these are exploratory experiments they should be done in triplicate including controls to increase confidence in the obtained results. Especially, since the proteins that differ in the two MS experiments were used to build networks (Fig. 7). The need for more LC-MS experiments is highlighted by the fact that the authors list PTBP1, U2AF2, KHDRBS1, SF3A3 and UTP18 as enriched compared to control in that replicate. Of these five RBPs, four (PTBP1, U2AF2, KHDRBS1, SF3A3) have eCLIP data available but only one of them supports the LC-MS picture (KHDRBS1 / Suppl File S5). This suggests that the LC-MS has a high rate of false positives.

- Another complication with the presented evidence of RBP binding the tRNA lookalikes is that there is no mention of the difference in signal between the RBP eCLIP and the corresponding IgG eCLIP from the same cell line (the eCLIP collection includes matching control IgG experiments for each RBP experiment and for the same cell line). Did the authors check that there is no IgG binding at the sites that emerge from the analysis? Which of the enrichments shown in Supp File S5 would survive if IgG data was taken into account?

- In the same section that describes the LC-MS experiment, there is mention of mitochondrial tRNA lookalikes. These are presumably human lookalikes. But which ones? Where are they located? What is their sequence conservation compared to the true mitochondrial tRNAs? (See above about the presence of the mitochondrial segment that includes the seven tRNAs in non-intronic regions of the human genome.)

- Finally, the eCLIP data analysis puts further into question the authors' very hypothesis. From the analyzed RBPs only three show enrichment at the mitochondrial tRNA lookalike sites: DHX30, G3BP1, and KHDRBS1. DHX30 is a component of the mitochondrial nucleoid and associates with mitochondrial DNA. If the mitochondrial tRNA lookalikes are exact copies of the mitochondrial tRNAs (as is the case of the lookalikes in the analyzed mouse cluster) it appears more likely that DHX30 is binding to the mitochondrial tRNA itself and not the intronic instance in the nuclear genome as the authors claim. The second RBP, G3BP1 is not known to be involved in splicing. Only the third RBP, KHDRBS1, is known to be involved in splicing but that is not its only role. Moreover the splicing connection hinges upon the assumption that the mitochondrial tRNA lookalike that is bound to KHDRBS1 is not expressed as a bona fide tRNA but only as part of an intron: this was not demonstrated for the human mitochondrial tRNA lookalikes that were analyzed by eCLIP, and, of course, still does not address the matter of the lookalikes that exist outside introns.

**Reviewer 2**

**Are you able to assess all statistics in the manuscript, including the appropriateness of statistical tests used?** Yes, and I have assessed the statistics in my report.

**Comments to author:**

Hoser et al identify mitochondrial tRNAs integrated in the human and mouse nuclear genomes focusing their subsequent analysis on mtRNAs present in introns of nuclear genes. They show that these nimtRNAs are not processed as bona fide tRNAs but, using a splicing reporter construct, demonstrate a role in the regulation of splicing, both constitutive and alternative. The effect of the number and position of nimtRNAs in introns is shown to be important for this functionality. Finally, specific splicing RBPs identified as binding nimtRNAs are characterized. Overall, the study is original and the findings are important as they provide a description of a new mechanism for the regulation of splicing. The data are generally convincing, but the text contains several confusing sections (missing figure legend parts, numbers that do not add up, missing labels in figures, missing explanations) that hinder clarity. In addition, some additional experiments could increase the impact of the work by confirming that nimtRNAs truly play a role in the mouse and/or human transcriptome, as describe below:

Major comments, by decreasing order of importance:

1. The figures presented lead to the conclusion that a few randomly chosen mouse nimtRNAs/mtRNAs well positioned in an intron can act as intronic splicing enhancers, having increased effect on exons harboring weak splicing sites. However, we have no idea if this mechanism really plays a role in the human/mouse transcriptome. From the 30 human protein-coding genes hosting nimtRNA, is there an example of a cassette exon that is influenced by the presence of nimtRNAs? Are nimtRNAs more often present in an intron upstream of a cassette exon than expected? CRISPR or a mini-gene of one of these 30 genes could in parallel be used to test whether a nimtRNA affects splicing and possibly protein expression.

2. Figure 3C is very hard to interpret. This is not helped by the fact that it has no y axis title, and no legend to indicate what the white, grey and black bars represent. The paragraph in the Results text relating to this figure is also confusing (bottom of page 10), in particular the explanation of why the total transcript levels don't strongly increase while the mRNA levels do. Having read it several times, I concluded that the reason for this is that the measured amounts in Figure 3C are ratios (of levels for constructs with the nimtRNAs and without), but this is not indicated in the figure legend, nor in the y axis title. Providing this essential information would help, but surely there is a better way to show the data (eg provide the abundance levels directly instead of ratios).

3. The authors could have done a siRNA on KHDRBS1 in the cells transfected with the mini-gene to see if there is any difference in the splicing enhancement (hence showing that KHDRBS1 is important for the splicing modulation). If this works, the point 1 could be addressed by doing a knock down of KHDRBS1 and looking at the splicing pattern and/or the protein expression of some of the 30 protein-coding genes.

4. The values provided in Table S1 and in Figure 1A are confusing for several reasons.

First, it is not indicated how TP and FP are defined using the genome-based strategy. It will be important to ensure that however the TP were determined did not involve using tRNAscan-SE and/or Infernal (for example, if RefSeq annotations were used to define TP, then it is important that RefSeq did not use tRNAScan-SE or Infernal to annotate tRNAs. If not, the identification of nmtRNAs would be circular).

Second, File hg38_nmt-tRNAs_all_core.ods has 1046 entries, which is confusing because in the first section of the Results, it is indicated that 726 human nmtRNAs were found. And the values in the Venn diagram (set 'own') of Figure 1A add up to 1041, not 1046. The values also don't add up for mouse.

5. Figure 5A, why use tyrosine (it is the second worst in terms of capacity to regulate splicing)? This should be justified. Why not alanine, which has a stronger effect? Similarly, at Line 417, why focus on nimtRNA-tyr in the EMSA? Your locus contains 5 nimtRNAs, why not try them all in your EMSA (or independently)?

6. It is still unclear how mtRNA compare to nimtRNA in terms of effect on splicing. Some examples of each were given, but they were never compared directly.

Minor comments:

Inconsistencies in the text that are important to correct

-Figure 1, there are two "(B)" sections in the legend and it is not clear what the second one is referring to.

-Figure S3D is not in the text, and not in the figure legend.

-Figure 7, the text says SNORD115 while it's written SNORD116 in the figure.

- Figure 5D: should it not be mtRNA-X and not nimtRNA-X, as it is written in the text and in the figure legend?

-Methods p22: Table S3 seems to be Table 3.

- Figure S7: add A and B in the figure and (B) in the legend

- Table S1 exists twice, once as a standalone file and once in as panel A of Figure S1. The standalone file should be removed as it has no legend.

Additional minor comments:

- In the Results section (p 5): 'In general, all methods tested partly yielded very different results'. What are methods tested partly?

- Figure 1D is hard to understand. The color legend is too small and some of the colors are too close (in particular the two darkest shades of red).

- Supplementary files 1 and 2: it would help to include a gene_name column.

- Figure 1B: it is very hard to see the difference between the grey point and the blue point in the legend, perhaps it would help to write "intronic" in blue in the legend box.

- Figure 2B, it would be interesting to add quantification for the northern to compare with the qPCR quantification in C.

- Suppl File S5, a header description in the file would be useful to explain what the columns are about.

- Figure S4 through S7 should be referred to in the Results.

- In the Methods: 'Since we cannot clearly identify hits outside if numtDNAs as nmtRNAs, we refer to them as mtRNA-lookalikes.' This sentence is confusing.

-Some spelling mistakes are found in the text and should be corrected (eg 'Splice acceptor and donor sites define exon/intron borders which is achieved…', '… either in HepG2 or K652…', 'Annotations where tested…',.

**Authors Response**

**Point-by-point responses to the reviewers’ comments:**

Rebuttal Letter

**Reviewer #1:**

Reviewer #1: *A significant portion of the early part of the manuscript is devoted to discovering mitochondrial tRNA like sequences in the nuclear genome. The Introduction and the presentation of the manuscript are structured in a way that suggests that the authors are the first to examine the question of mitochondrial tRNA insertions in the nuclear genome. This is tantamount to reinventing the wheel as this very study was originally described in Telonis et al Frontiers in Genetics 2014. While the authors mention this earlier paper they do not appropriately describe its scope and goal which was to discover full-length tRNA-like sequences in the nuclear genome (and not nuclear insertions of mitochondrial DNA as the authors claim in the Introduction).*

Reply: Reviewer #1 mentioned that we did not sufficiently recognize previous efforts in the field of genomic tRNA searches conducted by Telonis et al., including the scope of the study published in Frontiers in Genetics in 2014. We agree with this notion and therefore adapted our manuscript accordingly: “MtRNA genes are inserted into the nuclear genome as components of numtDNAs. In pioneering work the group of Telonis and co-workers previously scanned the nuclear genome for full-length mitochondrial tRNAs using a ‘Basic local alignment search tool’ (BLAST) approach and coined the term mitochondrial tRNA-lookalikes (MTLs) (28, 29).” Line 145 (but also mentioned at line 183 and 185)

Reviewer #1: *Telonis et al. labeled the sequences they discovered "tRNA-lookalikes" and the term has since been used in multiple papers. Even though the authors reference the paper, they ignore the earlier work and relabel the same sequences as "nuclear encoded mitochondrial tRNAs (nmtRNAs)". Instead the authors should use the original term "mitochondrial tRNA lookalikes" in order to put their work and new contributions in the proper context.*

Reply: We agree with the notion that Telonis et al. previously coined the term tRNA-lookalikes. We therefore changed the term nmtRNAs to mitochondrial tRNA-lookalikes (MTLs) in the entire manuscript. Intronic MTL sequences are however in the following referred to as nimtRNAs since we link them to a novel biological function.

Reviewer #1: *On a related note, in the section "Conservation of nmtRNAs and nimtRNAs" the authors discuss sequence conservation. They report that they found several MT tRNA lookalikes "that appear to have evolved significantly more slowly than the adjacent numtDNA sequences." The authors are again attempting to reinvent the wheel. In both Telonis et al Frontiers Gen 2014 and Telonis et al RNA Biology 2015, both of which are referenced by the authors, there is explicit discussion about the fact that specific mitochondrial tRNA lookalikes are conserved identically in the nuclear genome.*

Reply: The reviewer might mix up two distinct issues here: (1) Given the high rate of numtDNA insertion [see e. g. Bensasson et al. 2003, et al 2004, Hazkani-Covo 2017], there are of course MTLs that are identical to the contemporary mitochondrial tRNAs of the respective species. These are in particular MTLs originating from very recent insertion events. (2) We focus on MTLs that are present in comparably old numtDNAs. These have had enough time since their insertion to evolve in the nuclear genome. Therefore, the substitution rates of the MTLs and the surrounding numtDNA can be used to quantify selection pressures. We have included a detailed description of the process of numtDNA insertion and evolution within the background section (including an additional Figure 1), which we hope  helps to clarify this issue.

Reviewer #1: *On line 223 of this manuscript, the authors also claim that "the insertion of nimtRNA genes in the respective introns of nuclear genes might be a very recent evolutionary event, which might have occurred independently in different species in addition to potential retainment of pre-existing nimtRNAs." This is not true. The Telonis et al 2015 paper showed that the concept of mitochondrial tRNA lookalikes is pervasive across at least 200 My of evolution and that in organisms from primates to marsupials one can find several mitochondrial tRNAs that have exact copies in their nuclear genomes.*

Reply: This might be a misunderstanding. We do not claim that the process of MTL insertion is a recent evolutionary innovation. On the contrary, numtDNA insertion is a rapid process. Most individual numtDNAs and MTLs therefore are the result of recent insertion events. This is in particular true for MTLs that are identical to the contemporary mitochondrial tRNAs. We have not been able to trace a single MTL to an insertion event that pre-dates common ancestor of human and marsupials. On the other hand, there are examples of MTLs in both lineages. In fact, the cited paper (Telonis et al.) also argues that "tRNA lookalikes represent independent evolutionary events in some animals".

Due to the way in which sequence conservation is determined in several publications (among others also in Telonis et al.), conservation from genome browsers cannot be used for the conservation analysis of repetitive or multicopy sequences, including tRNAs (see Velandia et al. 2016). Conservation is assessed based on genome-wide alignments, which in turn is constructed using anchor-based methods, i.e., essentially local (blast) hits. As a consequence, newly inserted numtDNAs match the mitogenome and/or numtDNAs in other species. These sequence fragments are then placed into the local alignment blocks. This effect is fairly easy to detect in genome-wide alignments. While a deletion is shown for VERY closely related species, more distantly related species appear in the alignment. For example, in the browser image shown below, the recently inserted human numtDNA is clearly visible: it is identified as missing in the Rhesus Monkey, Dog and Elephant and then suddenly appears again in more distantly related species. Also note that the numtDNA shows "evidence" for expression from EST data: clearly an artefact of mapping data from mitochondrial RNAs.

Reviewer #1: *Many of the experiments that the authors describe involve a cluster of five mitochondrial tRNA lookalikes from intron 30 of Myo3a. In actuality there are seven such tRNAs and not five (the coordinates of all seven are in the manuscript's supplemental material). First, all seven of these mitochondrial tRNA lookalikes were reported in Telonis et al 2015 (see* [*https://cm.jefferson.edu/data-tools-downloads/trna-lookalikes-beyond-homo-sapiens/)*](https://cm.jefferson.edu/data-tools-downloads/trna-lookalikes-beyond-homo-sapiens/))*, and are not new discoveries.*

Reply: We agree with the comment that the nimtRNAs from the mouse Myo3a cluster were previously reported which we now included into the text as follows: “A cluster of five nimtRNA genes (out of the seven nimtRNAs of the *Myo3a* gene, Fig. 3A), previously reported to be present within intron 30 of the mouse *Myo3a* gene (35), was inserted into the intronic region of this splicing reporter.”  (line 281). Also, we show in Figure 3A that there are a total of seven nimtRNAs within the Myo3A intronic locus.

Reviewer #1: *Second, there is a methodological problem here as the authors arbitrarily dismiss two of the seven lookalikes. This is problematic in two ways. If the authors' hypothesis is that these intron-embedded lookalikes are functional then all seven sequences should be tested in the experiments and not only five. These seven tRNA lookalikes in intron 30 of Myo3a are in two groups: two tRNA lookalikes followed by a pseudo-copy of MT-CO1 followed by the other five tRNA lookalikes. This is the same arrangement one finds in the mitochondrial genome.*

Reply: Reviewer #1 notes that we only used five of the seven nimtRNAs present within this intron. We here list five reasons why we chose our approach in this manner and why we maintain that our data is valid:

1. A longer insert would hinder cloning approaches and the reporter splicing assay:

The five nimtRNAs used are clustered together closely and span a region of only 500 bp while the other two are 1.5 kb upstream which hindered reporter cloning. Furthermore, we needed to compare the nimtRNA-containing splicing reporter to the wt reporter and a bigger difference in intron length could have skewed the results.

2. “5 nimtRNAs were sufficient to produce a strong effect:

We observed that the insertion of the mentioned quintet of nimtRNAs increased mRNA abundance by almost 4-fold; thus a strong splicing effect was already observed when employing the respective five nimtRNAs, only.

3. Effect was nimtRNA-number-dependent:

We observed that the increase in mRNA abundance was nimtRNA number-dependent. Thus, we assumed a further increase of nimtRNA numbers to potentially increase the effects, but not to change the type of the splicing effect.

4. All nimtRNAs showed effects on their own:

We observed the same type of effect in different degrees of prominence for all the different nimtRNAs tested. Therefore, we do not expect a different type of effect for the two nimtRNAs upstream of the cluster of five nimtRNAs.

5. Intronic nimtRNAs come in different combinations:

NimtRNAs are found either as singles or in different combinations within the introns of their host genes. In terms of the “YCNAW” cluster used in our approach, the human CENPP gene contains exactly this combination. In the human AKAP6 gene the W is missing, in the JAK2 gene a total of 18 nimtRNAs can be found including the whole YCNAW cluster. We therefore suppose that the inclusion of two extra nimtRNAs would not qualitatively affect results.

We thus believe that the results obtained with the “YCNAW” cluster recapitulate in an exemplary manner the effect of intronic nimtRNAs and combinations thereof in splicing regulation.

Reviewer #1: *If one searches the mouse genome with this mitochondrial segment (2 tRNAs plus MT-CO1 plus 5 tRNAs) one finds matches in the nuclear genome for both the full segment and the individual tRNAs. Many of these copies hit regions that do not contain any known transcripts and are exact copies of the mitochondrial tRNA. If the hypothesis put forth is correct shouldn't all of these lookalikes be in introns, either by themselves or as groups? I do not see how the hypothesis is warranted if there are comparable numbers of copies in intronic and in non-intronic regions of the genome.*

Reply: Reviewer #1 questioned how the presence of intergenic, non-intronic nimtRNAs is in accordance with our hypothesis of nimtRNA-mediated splicing regulation. In the experimental part of this study, we focused on investigating the specific effects of nimtRNAs within the introns of genes and did not investigate the biological relevance of intergenic MTLs. The potential biological relevance of intergenic MTLs is clearly outside the scope of this study.

Reviewer #1: *Also, if the nuclear copies of this mitochondrial segment of 2 tRNAs plus MT-CO1 plus 5 tRNAs have a role in splicing in mouse, it is reasonable to assume that this is the case in the human genome as well. Here, a search reveals that the human orthologue of the mouse segment has several nearly full-length copies at different locations of the human nuclear genome that are not in the introns of any genes. This finding further questions the hypothesis that the mitochondrial tRNA lookalikes play a role in splicing.*

Reply: We thank the reviewer for this comment and agree with the notion of it being likely that the quintet of nimtRNAs does also have a role in the human genome. He/she mentions that “several nearly full-length copies at different locations of the human nuclear genome that are not in the introns of any genes”. He goes on to say that this “questions the hypothesis that the mitochondrial tRNA lookalikes play a role in splicing”. We propose that nimtRNAs are not exclusively found within intronic regions but also within intergenic regions (designated as MTLs), because MTLs, and numtDNA in general, are integrated into the nuclear genome by chance and not in a targeted manner. In most cases it is likely that intronic integration of numtDNA is detrimental to gene expression/function and cellular metabolism and will therefore be actively selected against.  Integration events occurring in intergenic regions on the other hand, would be less likely to be actively selected against as one would predict they are less likely to have a negative impact on cell function and survival. We do not exclude that nimtRNAs may have additional functions outside of introns, which might affect their conservation.

Reviewer #1: *Starting on line 231, the authors make the claim that "NimtRNAs from nuclear introns are not processed as bona fide tRNAs" but do not present any evidence from RNA-seq data from the public domain to support the claim. In fact, Telonis et al Frontiers Genet 2014 presented evidence that the authors' claim is not true: using ENCODE data Telonis et al showed for multiple tRNA lookalikes that they are expressed and have the expected lengths. Telonis et al also showed that the expression of tRNA lookalikes is cell-type specific. More recently, Dror et al PLoS Genetics 2016 showed that tRNA expression in general appears to be both cell-type and time-specific. Therefore one cannot exclude that the absence of expression of these tRNAs in the (single) cell line and (single) time point that was used by the authors is not a manifestation of this specificity.*

Reply: Reviewer 1 mentioned that we “do not present any evidence from RNA-seq data from the public domain” to claim that “nimtRNAs from nuclear introns are not processed as bona fide tRNAs”. We agree with this notion and thank the reviewer for making us aware of the points he mentioned. It is true, that we did not examine other cell types for the presence of processed nimtRNAs from either endogenous or exogeneous sources. We therefore adapted the text to reflect this by changing the heading of the section to “NimtRNAs located in introns of nuclear encoded pre-mRNAs are not processed as bona fide tRNAs in 293T cells” (line 274).

Reviewer #1: *The section that begins on line 259 describes a series of experiments involving microRNA miR-124. The authors claim that this microRNA has brain-specific expression and therefore is a good choice for use with HEK293 cells. This is incorrect because in humans miR-124 appears in multiple healthy and disease tissues. See for example:* [*http://www.mirbase.org/cgi-bin/get_read.pl?acc=MI0000445*](http://www.mirbase.org/cgi-bin/get_read.pl?acc=MI0000445) *where it is shown to be present in skin, cervix, lung, tonsil, kidney (sample SRR553575 mentioned on the mirbase page), etc. The presence of miR-124 in human kidneys puts into question the appropriateness of using HEK293 cells for these experiments.*

Reply: Reviewer #1 noted that microRNA miR-124 is also expressed in other tissues besides brain tissue, including the kidney and is therefore not suitable for the experimental setup. In our approach, we used the microRNA miR-124a from mouse as part of the splicing reporter for transient transfection into human embryonic kidney (HEK293T) cells. The respective probe was additionally designed to be specific for mouse miR124a-1 (specifically targeting differences of the mouse miRNA compared to the human miRNA) to avoid cross-hybridization. We thereby only wanted to determine, if the splicing reporter employed hinders canonical ncRNA processing, which it did not.

Reviewer #1: *I find the experiments in the section that starts on line 298 confusing.  Since the authors weakened the strength of the 5´ splice site shouldn't they be obtaining less spliced product compared to the premRNA? Figure 3C says that they obtain more spliced product compared to the premRNA which I do not understand. Did the authors check the length of the spliced product after weakening the splice site to confirm that it did not contain any new segment from the 2.2kb intron?*

Reply: The reviewer mentioned that he found “the experiments in the section that starts on line 298 confusing” including the less efficient splicing reporter and the quantification of mRNA, pre-mRNA and total reporter transcript levels. We agree with this notion and want to clarify our point. As the reviewer assumed correctly, the less efficient splicing reporter indeed results in less spliced transcript (about 450-fold less when comparing wt reporters, but “only” 110-fold when comparing the two different nimtRNA-containing reporters). In the first version of Fig. 3 (now Fig. 3F) we showed the relative fold-change of each type of transcript (mRNA, pre-mRNA total) in the respective reporter (Low0 and Low2 i.e. efficient and inefficient) upon nimtRNA presence i.e. Low0-YCNAW/Low0-wt and Low2-YCNAW/Low2-wt. To make our point clearer we adapted the figure in terms of comparing transcript levels and not ratios. The manuscript was adapted as follows: “As expected, the Low2 wt reporter resulted in an extensive, i.e. 450-fold, decrease in reporter mRNA abundance, compared to the more efficient Low0 wt reporter, while only an about 2-fold reduction in pre-mRNA and total transcript levels was observed. Upon nimtRNA insertion into the Low2 reporter mRNA, spliced mRNA abundance was about 38-fold lower compared to the Low0 wt construct. Thus, insertion of the YCNAW nimtRNA cluster resulted in an about 13-fold increase in spliced mRNA abundance in the inefficient Low2 splicing reporter, thus exhibiting a more pronounced effect on splicing than the efficient Low0 splicing reporter (showing an about 3-fold increase in mRNA abundance). Notably, pre-mRNA and total reporter transcript levels remained unchanged (Fig. 3F). This can be explained by reporter pre-mRNA being significantly more abundant than spliced mRNA.” (starting line 358)

The reviewer also asked for confirmation that the inefficient splicing reporter uses the same splice sites/does “not contain any new segment from the 2.2kb intron”. Unfortunately, it was not possible to detect reporter mRNA abundance by northern blot analysis due to the 110-450-fold decrease in mRNA abundance compared to the default Low0 wt reporter. Nevertheless, the Low2-eGFP splicing reporter was been validated and previously published {Erkelenz, 2018 #1056}.

Reviewer #1: *The experiments of the section that begins on line 321 use the corresponding tRNA lookalike sequences out of context. To convincingly show the role of these insertions the authors should introduce a sequence that is as long as the mitochondrial genome segment that spans the seven-lookalike cluster and scramble six of the seven lookalikes each time leaving the seventh one untouched. However, even if successful, the experiments would not address the role of the exact mitochondrial tRNA lookalikes that are found outside introns.*

Reply: The reviewer notes that we “use the corresponding tRNA lookalike sequences out of context”. We agree with his notion, but believe that this imitates what happens during the integration of numtDNA into the introns of nuclear genes, where we find different numbers of introns to be present. For a more detailed explanation as to why we believe our data recapitulates the effects of endogenous nimtRNAs, we refer to the 5 points mentioned above. We want to underline that the role of MTLs outside of introns is beyond the scope of this study.

Reviewer #1: *The description of the LC-MS experiments suggests that there was only a single replicate. Given that these are exploratory experiments they should be done in triplicate including controls to increase confidence in the obtained results. Especially, since the proteins that differ in the two MS experiments were used to build networks (Fig. 7). The need for more LC-MS experiments is highlighted by the fact that the authors list PTBP1, U2AF2, KHDRBS1, SF3A3 and UTP18 as enriched compared to control in that replicate. Of these five RBPs, four (PTBP1, U2AF2, KHDRBS1, SF3A3) have eCLIP data available but only one of them supports the LC-MS picture (KHDRBS1 / Suppl File S5). This suggests that the LC-MS has a high rate of false positives.*

Reply: Reviewer #1 made us aware of the replicate issue regarding the MS analysis. We agree with the notion that replicates are needed to validate the fold-change of proteins when comparing the two conditions. We want to emphasize that the MS data and inherent analyses were our first indications that the proteins might be of interest for nimtRNA-mediated splicing increase. Our subsequent analyses looked for an overlap (eCLIP) and also determined a single protein binding to a single nimtRNA in a structure-dependent manner (EMSA).

Since we were not able to replicate the MS experiments within the time of revision, we adapted our manuscript by only taking into consideration the proteins we found binding to the nimtRNA-containing transcript, where we cut out the bands and did MS analysis. We used this pool of proteins for a subsequent GO analysis which recapitulated our results from before, showing a network of splicing-related proteins binding to the nimtRNA-containing transcript. We go on by looking for an overlap with the publicly available and bioinformatically analyzed eCLIP data and determine KHDRBS1 to bind to a single nimtRNA in a structure-dependent manner. The adaption can be found in the manuscript starting at line 492 and is depicted in Fig. 6A, B and C.

Reviewer #1: *Another complication with the presented evidence of RBP binding the tRNA lookalikes is that there is no mention of the difference in signal between the RBP eCLIP and the corresponding IgG eCLIP from the same cell line (the eCLIP collection includes matching control IgG experiments for each RBP experiment and for the same cell line). Did the authors check that there is no IgG binding at the sites that emerge from the analysis?  Which of the enrichments shown in Supp File S5 would survive if IgG data was taken into account?*

Reply: We thank the reviewer for raising this question, as it clearly highlights one of the major advantages of eCLIP data in comparison to standard CLIP procedures without an IgG control. However, as we are aware of this fact we already included processed peaks derived from the eCLIP pipeline which only cover regions with a significant signal over control ratio after stringent quality control and thus report only RBP overlaps with our data where binding is likely, which is true for all enrichments listed in Supp File S5. To make this clearer we adapted the methods section accordingly. (These binding sites are already quality controlled and show enrichment after normalization against IgG background, for more information on data generation and processing please refer to <https://www.encodeproject.org/eclip/>)

Reviewer #1: *In the same section that describes the LC-MS experiment, there is mention of mitochondrial tRNA lookalikes. These are presumably human lookalikes. But which ones? Where are they located? What is their sequence conservation compared to the true mitochondrial tRNAs? (See above about the presence of the mitochondrial segment that includes the seven tRNAs in non-intronic regions of the human genome.)*

Reply: We want to reply to the comment that the nimtRNAs mentioned in the LC-MS were not properly introduced. In fact, the nimtRNAs employed were identical to those used for the experiments mentioned before, i.e. the YCNAW cluster of five nimtRNAs from mouse Myo3a intronic locus, which were already described in the manuscript. This is mentioned in the manuscript as follows: “

This approach utilized a biotinylated T7 transcript containing five nimtRNAs identical to those employed previously (i.e. YCNAW, see above) which was incubated with a nuclear extract generated from HEK 293T cells.” (line 498)

Reviewer #1: *Finally, the eCLIP data analysis puts further into question the authors' very hypothesis. From the analyzed RBPs only three show enrichment at the mitochondrial tRNA lookalike sites: DHX30, G3BP1, and KHDRBS1. DHX30 is a component of the mitochondrial nucleoid and associates with mitochondrial DNA. If the mitochondrial tRNA lookalikes are exact copies of the mitochondrial tRNAs (as is the case of the lookalikes in the analyzed mouse cluster) it appears more likely that DHX30 is binding to the mitochondrial tRNA itself and not the intronic instance in the nuclear genome as the authors claim. The second RBP, G3BP1 is not known to be involved in splicing. Only the third RBP, KHDRBS1, is known to be involved in splicing but that is not its only role. Moreover the splicing connection hinges upon the assumption that the mitochondrial tRNA lookalike that is bound to KHDRBS1 is not expressed as a bona fide tRNA but only as part of an intron: this was not demonstrated for the human mitochondrial tRNA lookalikes that were analyzed by eCLIP, and, of course, still does not address the matter of the lookalikes that exist outside introns.*

Reply: Please take into account that the enrichment we in show in Supp File S5 is adding an additional layer of information. One comes directly from eCLIP peaks, which, as stated above, are only called? if a significant enrichment of RBP binding over IgG control can be found for the region of interest. Additionally, we calculate the relative enrichment of RBP overlap with our data over background from binding sites in intronic regions and list those values in Supp File S5. The three mentioned RBPs are therefore not the only ones with significant peaks that overlap, but in addition they bind our sites more often than expected in comparison to other intronic regions.

We agree that the biological function of these interactions is so far not well characterized and as the reviewer points out we can only draw conclusions from already published functions of these RBPs, as investigations into this matter beyond the experiments already conducted require dedicated studies which are outside the scope of this manuscript. We agree with the reviewer that there are no indications that DHX30 is present in the nucleus and thus we excluded it from our results. Not much is known about the role of G3BP1 is in general, other than its ability to interact with RNA and DNA and its unwinding capabilities for duplexes of RNA/DNA and RNA/RNA, but it has been found in the nuclear compartment, which is why we did not exclude it.

*Reviewer #1 has stated that “the eCLIP data analysis puts further into question the authors' very hypothesis” referring to the sparsity of overlap between eCLIP and MS analyses.*

Reply: We agree that it is impossible to exclusively map binding sites to either the mitochondrial tRNA or the intronic counterpart if they are exact sequence copies and no flanking sequence is taken into account. This is of course true for all regions in the genome which harbor identical or near identical sequences. As many RBPs are, however, known for their sequence specific binding preferences, binding to both sequences is equally likely, given that both RBP and target are available for binding which is the case here. Furthermore, the downloaded eCLIP peaks undergo stringent quality control, including removing of mappings to repeat regions and minimum coverage of peak regions by uniquely mappable reads as stated in https://www.encodeproject.org/documents/739ca190-8d43-4a68-90ce-1a0ddfffc6fd/@@download/attachment/eCLIP_analysisSOP_v2.2.pdf.

*Moreover the splicing connection hinges upon the assumption that the mitochondrial tRNA lookalike that is bound to KHDRBS1 is not expressed as a bona fide tRNA but only as part of an intron: this was not demonstrated for the human*

*mitochondrial tRNA lookalikes that were analyzed by eCLIP, and, of course, still does not address the matter of the lookalikes that exist outside introns.*

We thank the reviewer for bringing up this issue. We took this into account which is why we included an additional 10 nucleotides up- and downstream of the nimtRNA^Tyr^ from the endogenous Myo3a locus in mouse in the transcript used for the electrophoretic mobility shift assay. This is now included in the text as follows: “To this end, a transcript of nimtRNA^Tyr^ including an extra 10 nucleotides at the 5’ and 3’ termini, in order to not resemble processed tRNA ends, was radioactively labeled and incubated with increasing concentrations of PTBP1 protein.” (Line 518)

**Reviewer #2:**

Reviewer #2: *1. The figures presented lead to the conclusion that a few randomly chosen mouse nimtRNAs/mtRNAs well positioned in an intron can act as intronic splicing enhancers, having increased effect on exons harboring weak splicing sites. However, we have no idea if this mechanism really plays a role in the human/mouse transcriptome. From the 30 human protein-coding genes hosting nimtRNA, is there an example of a cassette exon that is influenced by the presence of nimtRNAs? Are nimtRNAs more often present in an intron upstream of a cassette exon than expected? CRISPR or a mini-gene of one of these 30 genes could in parallel be used to test whether a nimtRNA affects splicing and possibly protein expression.*

Reply: Reviewer #2 states that “The figures presented lead to the conclusion that a few randomly chosen mouse nimtRNAs/mtRNAs well positioned in an intron can act as intronic splicing enhancers, having increased effect on exons harboring weak splicing sites. However, we have no idea if this mechanism really plays a role in the human/mouse transcriptome.” We agree with this notion and therefore did a CRISPR/Cas9 knock-out of an endogenous nimtRNA, i.e. nimtRNA^Lys^ which can be found within an intron of the PPFIBP1 gene. We were able to show that different nimtRNA deletions considered in isolation and also when combined in the bulk of all CRISPR-targeted cells result in a decrease of downstream exon inclusion. Concomitantly, the abundance of the transcript lacking the downstream exon increased. Thus, we show that an endogenous nimtRNA increases downstream exon inclusion (section: CRISPR/Cas9 mediated partial deletion of endogenous nimtRNA^Lys^ within intron 28 of the *PPFIBP1* gene results in decreased downstream exon 29 inclusion starting on line 449)

Reviewer #2: *2. Figure 3C is very hard to interpret. This is not helped by the fact that it has no y axis title, and no legend to indicate what the white, grey and black bars represent. The paragraph in the Results text relating to this figure is also confusing (bottom of page 10), in particular the explanation of why the total transcript levels don't strongly increase while the mRNA levels do. Having read it several times, I concluded that the reason for this is that the measured amounts in Figure 3C are ratios (of levels for constructs with the nimtRNAs and without), but this is not indicated in the figure legend, nor in the y axis title. Providing this essential information would help, but surely there is a better way to show the data (eg provide the abundance levels directly instead of ratios).*

Reply: We thank Reviewer #2 for making us aware of the lack of clarity for Figure 3C (now Fig. 3F) and the respective paragraph. The reviewer correctly assumed that the values indicated are the respective ratios. To improve the comprehensibility of this data set/experiment, we adapted the figure to showing the relative-fold change compared to the efficient Low0 wt reporter for all transcripts rather than the ratios. Furthermore, we adapted the manuscript as follows:” As expected, the Low2 wt reporter resulted in an extensive, i.e. 450-fold, decrease in reporter mRNA abundance, compared to the more efficient Low0 wt reporter, while only an about 2-fold reduction in pre-mRNA and total transcript levels was observed. Upon nimtRNA insertion into the Low2 reporter mRNA, spliced mRNA abundance was about 38-fold lower compared to the Low0 wt construct. Thus, insertion of the YCNAW nimtRNA cluster resulted in an about 13-fold increase in spliced mRNA abundance in the inefficient Low2 splicing reporter, thus exhibiting a more pronounced effect on splicing than the efficient Low0 splicing reporter (showing an about 3-fold increase in mRNA abundance). Notably, pre-mRNA and total reporter transcript levels remained unchanged (Fig. 3F). This can be explained by reporter pre-mRNA being significantly more abundant than spliced mRNA. (line 358)

Reviewer #2: *3. The authors could have done a siRNA on KHDRBS1 in the cells transfected with the mini-gene to see if there is any difference in the splicing enhancement (hence showing that KHDRBS1 is important for the splicing modulation). If this works, the point 1 could be addressed by doing a knock down of KHDRBS1 and looking at the splicing pattern and/or the protein expression of some of the 30 protein-coding genes.*

Reply: We agree with this notion. We indeed did perform an RNAi-mediated knock-down which we co-transfected either at the same time as or 60 hours prior to splicing reporter transfection, either the constitutive splicing reporter Low0 or the alternative splicing reporter Designer Exon. By this approach we obtained inconclusive results in respective pre-mRNA splicing.

Potential reasons for the incapability of a KHDRBS1 knock-down to affect nimtRNA reporter host gene splicing might be that

- KHDRBS1 is not a limiting factor, i.e. a 70% knock-down would not affect the nimtRNA-mediated splicing increase
- KHDRBS1 can be replaced by a protein with a redundant function
- More importantly, KHDRBS1 is a protein with reported multiple general functions in splicing and other biological processes; a knock-down is thus expected to result in pleiotropic effects that may hinder the respective splicing analysis

Reviewer #2: *4. The values provided in Table S1 and in Figure 1A are confusing for several reasons.*

*First, it is not indicated how TP and FP are defined using the genome-based strategy. It will be important to ensure that however the TP were determined did not involve using tRNAscan-SE and/or Infernal (for example, if RefSeq annotations were used to define TP, then it is important that RefSeq did not use tRNAScan-SE or Infernal to annotate tRNAs. If not, the identification of nmtRNAs would be circular).*

*Second, File hg38_nmt-tRNAs_all_core.ods has 1046 entries, which is confusing because in the first section of the Results, it is indicated that 726 human nmtRNAs were found. And the values in the Venn diagram (set 'own') of Figure 1A add up to 1041, not 1046. The values also don't add up for mouse.*

Reply:  We thank Reviewer #2 for making us aware of the lack of clarity in this issue. We added an explanation to the results part: “Since for each numtDNA the original mtDNA sequence is known, we used this synteny information to validate our results. For each method we classified the detected hits as true positives (TPs) if they were found in the corresponding numtDNA as described by their synteny of the originating mitochondrial DNA. The remaining hits were designated as false positives (FPs).” (Line 211). We also extended the explanation in the methods section. We corrected the numbers in the Venn diagram (Fig. 1A which is now Fig. 2A). The explanation is given in Line 224: “Finally, we identified 731 MTLs within recognizable numtDNA (42 MTLs (*NUMT*-based method) + 684 (*NUMT*- and *genome*-based method) + 5 (*genome*-based method)) and 92 MTLs within recognizable numtDNA (16 MTLs (*NUMT*-based method) + 73 (*NUMT*- and *genome*-based method) + 3 (*genome*-based method)) in human and mouse genomes, respectively (Fig. 2A).”.

Reviewer #2: *5. Figure 5A, why use tyrosine (it is the second worst in terms of capacity to regulate splicing)? This should be justified. Why not alanine, which has a stronger effect? Similarly, at Line 417, why focus on nimtRNA-tyr in the EMSA? Your locus contains 5 nimtRNAs, why not try them all in your EMSA (or independently)?*

Reply: We thank the reviewer for bringing up the question of why we specifically used nimtRNA^Tyr^ for our experiments. We agree that the effects observed are trumped by the effects of other nimtRNAs analyzed as well. Our preliminary bioinformatic analyses indicated that nimtRNAs of the isotype Tyrosine seemed to exhibit a well conserved secondary structure. Thus, we based the following experiments including the deletion of different nimtRNA regions and positioning experiments on nimtRNA^Tyr^. Furthermore, we propose that a 1.9-fold increase in host mRNA abundance might be a reasonable modulatory effect of a splicing regulatory element.

In our electrophoretic mobility shift assay we employed nimtRNA^Tyr^ because we knew that the T-arm deletion variant did not increase splicing, which could therefore be used as a biological control. A longer transcript covering all five different nimtRNAs from the same locus would have hindered the gel shift procedure (we would have to switch from PAA to agarose) and would have added another level of uncertainty regarding specificity (since there are some short sequences between nimtRNAs).

Reviewer #2: *6. It is still unclear how mtRNA compare to nimtRNA in terms of effect on splicing. Some examples of each were given, but they were never compared directly.*

Reply: We thank the reviewer for bringing this up and agree with his notion. We therefore conducted a series of experiments comparing the effects of human and mouse mitochondrial tRNA^Ser2^ with different variants of the nimtRNA^Ser2^ found within different intronic loci in humans. We included these experiments in the section “nimtRNAs increase alternative exon inclusion in a three-exon splicing reporter”. We thereby observed different prominence of effects on alternative exon inclusion dependent on the nimtRNA variant.

Reviewer #2: *Minor comments:*

*Inconsistencies in the text that are important to correct*

*-Figure 1, there are two "(B)" sections in the legend and it is not clear what the second one is referring to.*

Reply: We agree with reviewer and adapted Figure 1 (now Fig. 2) accordingly.

Reviewer #2: *-Figure S3D is not in the text, and not in the figure legend.*

Reply: We agree with the reviewer and adapted the figure accordingly.

Reviewer #2: -*Figure 7* (now Figure 6F)*, the text says SNORD115 while it's written SNORD116 in the figure.*

Reply: We agree with the reviewer and adjusted the manuscript accordingly; SNORD115 was used in the experiments mentioned here.

Reviewer #2: - *Figure 5D* (now Fig. 4F)*: should it not be mtRNA-X and not nimtRNA-X, as it is written in the text and in the figure legend?*

Reply: We thank the reviewer for making us aware of this inconsistency and agree with this notion. We therefore adapted the figure accordingly.

Reviewer #2: *-Methods p22: Table S3 seems to be Table 3.*

Reply: We agree with the reviewer’s observation and adjusted the manuscript accordingly.

Reviewer #2: *- Figure S7: add A and B in the figure and (B) in the legend*

Reply: We thank the reviewer for this suggestion. Figure S7B has been removed from the Supplemental information.

Reviewer #2: *- Table S1 exists twice, once as a standalone file and once in as panel A of Figure S1. The standalone file should be removed as it has no legend.*

Reply: We thank the reviewer for this suggestion and removed the standalone file.

Reviewer #2: *Additional minor comments:*

*- In the Results section (p 5): 'In general, all methods tested partly yielded very different results'. What are methods tested partly?*

Reply: We thank the reviewer for this suggestion. We added the explanation of the tested methods to the beginning of the section.

Reviewer #2: - *Figure 1D is hard to understand. The color legend is too small and some of the colors are too close (in particular the two darkest shades of red).*

Reply:  We agree with the reviewer’s observation. We enlarged the legend of Fig. 1D (now Fig. 2D). We are not able to change the color code, since it is predefined by the LocARNA package.

Reviewer #2: *- Supplementary files 1 and 2: it would help to include a gene_name column.*

Reply: We thank the reviewer for this suggestion. We separated the column “Transcript” into “Transcript type” and “Transcript ID”, so that it becomes clearer.

Reviewer #2: *- Figure 1B: it is very hard to see the difference between the grey point and the blue point in the legend, perhaps it would help to write "intronic" in blue in the legend box.*

Reply: We agree with the reviewer’s observation and we therefore adapted the Fig. 1B (now Fig. 2B) accordingly.

Reviewer #2: *- Figure 2B, it would be interesting to add quantification for the northern to compare with the qPCR quantification in C.*

Reply: Reviewer #2 suggested to include a quantification of the northern blot analysis in Fig. 2B (now Fig. 3B). There are two reasons why the quantification of the northern blot does not enhance the clarity of the results.

- Transfection efficiency: the transfection of cells is a procedure that is very sensitive to the initial conditions and differences in transfection efficiency between different constructs can occur. Therefore, we included a transfection control for which we performed normalization in our RT-qPCR assay rather than to an endogenous mRNA. In the northern blot analysis shown here the transfection efficiencies also vary slightly and as such it is neither possible to compare the samples directly nor as ratios of target (eGFP) and control (dsred2express) mRNA.
- The detection of signal is limited by the maximum exposure after which no further increase in signal can be detected and the minimum exposure which is needed to observe a signal for all constructs. Thus, the northern blot analysis only serves to validate the trend observed in the RT-qPCR.

Reviewer #2: *- Suppl File S5, a header description in the file would be useful to explain what the columns are about.*

Reply:  We agree with the reviewer’s observation and have added a description in the file.

Reviewer #2: *- Figure S4 through S7 should be referred to in the Results.*

Reply: We thank the reviewer for making us aware of this inconsistency. The manuscript has been adapted accordingly.

Reviewer #2: - *In the Methods: 'Since we cannot clearly identify hits outside if numtDNAs as nmtRNAs, we refer to them as mtRNA-lookalikes.' This sentence is confusing.*

Reply: We thank the reviewer for this suggestion. By changing the names of nmtRNAs to mitochondrial tRNA-lookalikes (MTLs; proposed by Reviewer 1), we hopefully removed the confusion. Now, we subdivide it into MTLs within (TPs) or outside (FPs) recognizable numtDNA. We added an external explanation to the section “Numerous, so far unidentified nimtRNAs are Present in Nuclear Genomes”.

Reviewer #2: *-Some spelling mistakes are found in the text and should be corrected (eg 'Splice acceptor and donor sites define exon/intron borders which is achieved…', '… either in HepG2 or K652…', 'Annotations where tested…',.*

Reply: We thank the reviewer for this notion and adapted the manuscript accordingly.

**Second round of review**

**Reviewer 1**

I thank the authors for their detailed answers. Below I list the items that remain outstanding.

Reviewer #1: Telonis et al. labeled the sequences they discovered "tRNA-lookalikes" and the term has since been used in multiple papers.

Even though the authors reference the paper, they ignore the earlier work and relabel the same sequences as "nuclear encoded mitochondrial tRNAs (nmtRNAs)". Instead the authors should use the original term "mitochondrial tRNA lookalikes" in order to put their work and new contributions in the proper context.

Reply: We agree with the notion that Telonis et al. previously coined the term tRNA- lookalikes. We therefore changed the term nmtRNAs to mitochondrial tRNA-lookalikes (MTLs) in the entire manuscript. Intronic MTL sequences are however in the following referred to as nimtRNAs since we link them to a novel biological function.

As a group of sequences/molecules the mitochondrial lookalikes were reported and labeled in 2014. I find the offered justification for renaming them puzzling. Why do the authors insist on renaming a category of sequences, the mitochondrial tRNA lookalikes, that have had a name for six years already?

Reviewer #1: On a related note, in the section "Conservation of nmtRNAs and nimtRNAs" the authors discuss sequence conservation. They report that they found several MT tRNA lookalikes "that appear to have evolved significantly more slowly than the adjacent numtDNA sequences." The authors are again attempting to reinvent the wheel. In both Telonis et al Frontiers Gen 2014 and Telonis et al RNA Biology 2015, both of which are referenced by the authors, there is explicit discussion about the fact that specific mitochondrial tRNA lookalikes are conserved identically in the nuclear genome.

Reply: The reviewer might mix up two distinct issues here: (1) Given the high rate of numtDNA insertion [see e. g. Bensasson et al. 2003, et al 2004, Hazkani-Covo 2017], there are of course MTLs that are identical to the contemporary mitochondrial tRNAs of the respective species. These are in particular MTLs originating from very recent insertion events. (2) We focus on MTLs that are present in comparably old numtDNAs. These have had enough time since their insertion to evolve in the nuclear genome. Therefore, the substitution rates of the MTLs and the surrounding numtDNA can be used to quantify selection pressures. We have included a detailed description of the process of numtDNA insertion and evolution within the background section (including an additional Figure 1), which we hope helps to clarify this issue.

The Introduction as amended contains a number of statements between lines 118 and 174 about mtDNA insertions in the nuclear genome. Given that these statements try to build a case for the presented work it is important that they be supported by relevant references. The authors should include these references.

In fact, some of the claims do not agree with known findings. For example on line 124 it is stated that "Insertions appear uniformly across the genome". This is not the case for the tRNA lookalikes. In Telonis et al Frontiers Gen 2014 it was shown that the distribution of the tRNA lookalikes is not uniform across the nuclear genome and that the lookalikes co-localize with the known tRNAs, which themselves have a biased distribution. Also, these insertions are biased in favor of specific isoacceptors (see Fig. 3 of Telonis et al). How do the authors reconcile their claims with the current literature?

Reviewer #1: On line 223 of this manuscript, the authors also claim that "the insertion of nimtRNA genes in the respective introns of nuclear genes might be a very recent evolutionary event, which might have occurred independently in different species in addition to potential retainment of pre-existing nimtRNAs." This is not true. The Telonis et al 2015 paper showed that the concept of mitochondrial tRNA lookalikes is pervasive across at least 200 My of evolution and that in organisms from primates to marsupials one can find several mitochondrial tRNAs that have exact copies in their nuclear genomes.

Reply: This might be a misunderstanding. We do not claim that the process of MTL insertion is a recent evolutionary innovation. On the contrary, numtDNA insertion is a rapid process. Most individual numtDNAs and MTLs therefore are the result of recent insertion events. This

is in particular true for MTLs that are identical to the contemporary mitochondrial tRNAs. We have not been able to trace a single MTL to an insertion event that pre-dates common ancestor of human and marsupials. On the other hand, there are examples of MTLs in both lineages.

In fact, the cited paper (Telonis et al.) also argues that "tRNA lookalikes represent independent evolutionary events in some animals". Due to the way in which sequence conservation is determined in several publications (among others also in Telonis et al.), conservation from genome browsers cannot be used for the conservation analysis of repetitive or multicopy sequences, including tRNAs (see Velandia et al. 2016). Conservation is assessed based on genome-wide alignments, which in turn is constructed using anchor-based methods, i.e., essentially local (blast) hits. As a consequence, newly inserted numtDNAs match the mitogenome and/or numtDNAs in other species. These sequence fragments are then placed into the local alignment blocks. This effect is fairly easy to detect in genome-wide alignments. While a deletion is shown for VERY closely related species, more distantly related species appear in the alignment. For example, in the browser image shown below, the recently inserted human numtDNA is clearly visible: it is identified as missing in the Rhesus Monkey, Dog and Elephant and then suddenly appears again in more distantly related species.

I agree with the methodology that the authors describe here. However it is based on the assumption that the further back in time a NUMT sequence entered the nuclear genome the more divergent it is. Does the assumption hold for the segments of the NUMTs where the tRNA lookalikes are found? Telonis et al 2014 show that the lookalikes are depleted in polymorphisms which suggests evolutionary pressure to maintain their sequences.

Also note that the numtDNA shows "evidence" for expression from EST data: clearly an artefact of mapping data from mitochondrial RNAs.

Here the authors essentially state that the EST mapping to which they refer, presumably drawn from the UC Santa Cruz genome browser, is incorrect: what evidence do the authors have to support this claim? And isn't this claim contradicting the authors' own argument? If the mapping of EST data to numtDNA is an "artefact" shouldn't the mapping of the eCLIP data to numtDNA also be an "artefact"? Yet, the authors use the latter mapping to support their own analyses.

Other comments here:

a) I note that the authors mention an image in their reply but an image was not included with the document that I received.

b) Out of curiosity I checked A. thaliana's genome and it contains mitochondrial tRNA lookalikes as well. So it appears that plants do make use of lookalikes even though, for example, the concept is not present in invertebrates.

Reviewer #1: Many of the experiments that the authors describe involve a cluster of five mitochondrial tRNA lookalikes from intron 30 of Myo3a. In actuality there are seven such tRNAs and not five (the coordinates of all seven are in the manuscript's supplemental material). First, all seven of these mitochondrial tRNA lookalikes were reported in Telonis et al 2015 (see https://cm.jefferson.edu/data-tools-downloads/trna- lookalikes-beyond-homo-sapiens/), and are not new discoveries.

Reply: We agree with the comment that the nimtRNAs from the mouse Myo3a cluster were previously reported which we now included into the text as follows: "A cluster of five nimtRNA genes (out of the seven nimtRNAs of the Myo3a gene, Fig. 3A), previously reported to be present within intron 30 of the mouse Myo3a gene (35), was inserted into the intronic region of this splicing reporter." (line 281). Also, we show in Figure 3A that there are a total of seven nimtRNAs within the Myo3A intronic locus.

On a related note, lines 189-192 state "Applying the computational annotation workflow presented here, we were able to identify numerous novel MTLs and nuclear encoded intronic mitochondrial-derived tRNA genes (designated as nimtRNAs) in humans and mice. Notably, nimtRNAs were always flanked by sequences of mitochondrial origin." Looking at the Excel of File S1 I see more than one thousand tRNA lookalike entries and many claimed NUMT regions. I could not find a caption for the file and it is not clear what it describes. A detailed description is needed.

In addition, the authors should mark as separate columns which of the NUMT regions listed here are already known in the literature. Specifically for the mitochondrial lookalikes contained in these NUMT regions, they should mark in separate columns those that were previously reported in Telonis et al 2014.

Doesn't Infernal/tRNAscan provide a quality score and probability values for the matches? If so the authors should add these as separate columns. This is an important point because I am not convinced that all of the reported mitochondrial lookalikes that appear in intronic regions (File S1) are correct. For example, I selected randomly ten among the shown intronic lookalike sequences and searched the genome with BLAT: half of them are problematic which raises an important concern. For example,

- ATAGTTTAACCAAAACATTAGATTGTGAATCTAACAATAGAAGCCTGCAACTTCTTGT in the intron of ENSG00000198216 is 58 nt long but nucleotides 38 through 58 are not present in any MT tRNA as they should

- GTTAAGAAGAGGAGTTGAAATTCTGATTATAAAGTTTTAGGTTTTATGCAATTGCTGGGCTCTGCCATCTTAAC in the intron of ENSG00000204740 is 74 nt long but nucleotides 1 through 29 are not present in the best matching MT tRNA as they should

- AGATATTAGTAAAATTATTTCCTAACTTTGCCAAAGTTAAGTTATAAGTTAAACCCTGTATATCTTA in the intron of gene ENSG00000185352 is 67 nt long but there is no evident alignment to any of the 22 MT tRNAs

- GTAAATAAGAAGTTGCGGACTTCTATTATTAGACCACAATCTAACATTTTGATTAAACTATATTTAC in the intron of gene ENSG00000003400 is 67 nt long but there is no evident alignment to any of the 22 MT tRNAs

- AAAGTGTCGCTTAACCCAAAGCATCCAGCTTACACCCGGAAGATTTCATCACAACCTGGTCACTTTGA in the intron of gene ENSG00000096968 is 68 nt long but there is no evident alignment to any of the 22 MT tRNAs

Perhaps the other, non-intronic, novel sequences shown in this file include problematic sequences too. If sequences were to be removed following manual inspection, then the reported findings that are based on the results of File S1 would be affected, as would the claims based on those findings.

Reviewer #1: Second, there is a methodological problem here as the authors arbitrarily dismiss two of the seven lookalikes. This is problematic in two ways. If the authors' hypothesis is that these intron-embedded lookalikes are functional then all seven sequences should be tested in the experiments and not only five. These seven tRNA lookalikes in intron 30 of Myo3a are in two groups: two tRNA lookalikes followed by a pseudo-copy of MT-CO1 followed by the other five tRNA lookalikes. This is the same arrangement one finds in the mitochondrial genome.

Reply: Reviewer #1 notes that we only used five of the seven nimtRNAs present within this intron. We here list five reasons why we chose our approach in this manner and why we maintain that our data is valid:

1.A longer insert would hinder cloning approaches and the reporter splicing assay:

The five nimtRNAs used are clustered together closely and span a region of only 500 bp while the other two are 1.5 kb upstream which hindered reporter cloning. Furthermore, we needed to compare the nimtRNA-containing splicing reporter to the wt reporter and a bigger difference in intron length could have skewed the results.

2."5 nimtRNAs were sufficient to produce a strong effect:

We observed that the insertion of the mentioned quintet of nimtRNAs increased mRNA abundance by almost 4-fold; thus a strong splicing effect was already observed when employing the respective five nimtRNAs, only.

3.Effect was nimtRNA-number-dependent:

We observed that the increase in mRNA abundance was nimtRNA number-dependent. Thus, we assumed a further increase of nimtRNA numbers to potentially increase the effects, but not to change the type of the splicing effect.

4.All nimtRNAs showed effects on their own:

We observed the same type of effect in different degrees of prominence for all the different nimtRNAs tested. Therefore, we do not expect a different type of effect for the two nimtRNAs upstream of the cluster of five nimtRNAs.

5.Intronic nimtRNAs come in different combinations:

NimtRNAs are found either as singles or in different combinations within the introns of their host genes. In terms of the "YCNAW" cluster used in our approach, the human CENPP gene contains exactly this combination. In the human AKAP6 gene the W is missing, in the JAK2 gene a total of 18 nimtRNAs can be found including the whole YCNAW cluster. We therefore suppose that the inclusion of two extra nimtRNAs would not qualitatively affect results. We thus believe that the results obtained with the "YCNAW" cluster recapitulate in an exemplary manner the effect of intronic nimtRNAs and combinations thereof in splicing regulation.

I understand the technical problem with the reporter cloning (Reason #1). However, Reason #3 is an assumption and no data has been shown to support it. Reason #5 is simply speculation and is not supported by any presented data either. Reason #4 suggests that the two lookalikes that have not been tested could be functional. To state that the authors "do not expect a different type of effect" is not adequate and supporting data should be provided. The authors should test at least one and ideally both of the remaining lookalikes for their ability to affect splicing. Finally, Reason #2 is not sufficient by itself. If the remaining two lookalikes do not affect splicing then wouldn't that contradict the authors' conjecture about the role of the intronic tRNA lookalikes?

Based on the data so far, the authors showed for a single instance of an intronic embedding that some of the tRNA lookalikes affect splicing. The sequences are present in the introns of a mouse gene but tested in a human cell line, a choice that introduces new and unknown variables that I discuss below. Then the authors use the fact that similar sequence embeddings exist in the introns of other genes to extrapolate and conclude that all such embeddings affect splicing. I think that this is quite a leap. It is fine to conjecture it in the Discussion but in my opinion the authors have not presented adequate data to allow the generalization mentioned in the title of the manuscript and in the text.

Reviewer #1: If one searches the mouse genome with this mitochondrial segment (2 tRNAs plus MT-CO1 plus 5 tRNAs) one finds matches in the nuclear genome for both the full segment and the individual tRNAs. Many of these copies hit regions that do not contain any known transcripts and are exact copies of the mitochondrial tRNA. If the hypothesis put forth is correct shouldn't all of these lookalikes be in introns, either by themselves or as groups? I do not see how the hypothesis is warranted if there are comparable numbers of copies in

intronic and in non-intronic regions of the genome.

Reply: Reviewer #1 questioned how the presence of intergenic, non-intronic nimtRNAs is in accordance with our hypothesis of nimtRNA-mediated splicing regulation. In the experimental part of this study, we focused on investigating the specific effects of nimtRNAs

within the introns of genes and did not investigate the biological relevance of intergenic MTLs. The potential biological relevance of intergenic MTLs is clearly outside the scope of this study.

I maintain that this point is relevant and goes to the heart of the authors' claim. The authors report 731 mitochondrial tRNA lookalikes in the human genome of which half are novel. Of these, 109 are present in the introns of 29 protein coding genes as per File S1. For one of the genes the authors showed that these embeddings are relevant for splicing. Even if this holds true for the other 28 genes, which has not been shown, there is still the matter of 622 lookalikes that are not inside introns. If they are non-functional then one would be inclined to conclude that the intronic embeddings were a random event in the first place and were subsequently co-opted to a new function (modulation of splicing). But as we already discussed the embeddings of the lookalikes in the human genome are not random. If they are not random, one would expect that the same sequence would have the same function independent of where in the genome it was embedded. This would in turn suggest that the role of the embeddings should be the same for both the intronic and intergenic embeddings. However, the proposed function (splicing) is claimed to be relevant only for the intronic subset. How do the authors reconcile these observations? To simply state that they choose to not investigate the role of the intergenic copies is not adequate. I will discuss this point again below.

Reviewer #1: Also, if the nuclear copies of this mitochondrial segment of 2 tRNAs plus MT-CO1 plus 5 tRNAs have a role in splicing in mouse, it is reasonable to assume that this is the case in the human genome as well. Here, a search reveals that the human orthologue of the mouse segment has several nearly full-length copies at different locations of the human nuclear genome that are not in the introns of any genes. This finding further questions the hypothesis that the mitochondrial tRNA lookalikes play a role in splicing.

Reply: We thank the reviewer for this comment and agree with the notion of it being likely that the quintet of nimtRNAs does also have a role in the human genome. He/she mentions that "several nearly full-length copies at different locations of the human nuclear genome that

are not in the introns of any genes". He goes on to say that this "questions the hypothesis that the mitochondrial tRNA lookalikes play a role in splicing". We propose that nimtRNAs are not exclusively found within intronic regions but also within intergenic regions (designated as MTLs), because MTLs, and numtDNA in general, are integrated into the nuclear genome by chance and not in a targeted manner. In most cases it is likely that intronic integration of numtDNA is detrimental to gene expression/function and cellular metabolism and will therefore be actively selected against. Integration events occurring in intergenic regions on the other hand, would be less likely to be actively selected against as one would predict they are less likely to have a negative impact on cell function and survival. We do not exclude that nimtRNAs may have additional functions outside of introns, which might affect their conservation.

Here the authors base their argument on the assumption that the tRNA lookalike embeddings occur randomly in the genome. As we saw above this is not the case. The point remains: if these embeddings occur both in intergenic and intronic regions one cannot interpret the intronic embeddings in isolation. Especially when the exact intronic embedding with which the authors experimented in mouse, is present outside introns in humans (and I would imagine in mouse too).

Reviewer #1: Starting on line 231, the authors make the claim that "NimtRNAs from nuclear introns are not processed as bona fide tRNAs" but do not present any evidence from RNA-seq data from the public domain to support the claim. In fact, Telonis et al Frontiers Genet 2014 presented evidence that the authors' claim is not true: using ENCODE data Telonis et al showed for multiple tRNA lookalikes that they are expressed and have the expected lengths. Telonis et al also showed that the expression of tRNA lookalikes is cell-type specific. More recently, Dror et al PLoS Genetics 2016 showed that tRNA expression in general appears to be both cell-type and time-specific. Therefore one cannot exclude that the absence of expression of these tRNAs in the (single) cell line and (single) time point that was used by the authors is not a manifestation of this specificity.

Reply: Reviewer 1 mentioned that we "do not present any evidence from RNA-seq data from the public domain" to claim that "nimtRNAs from nuclear introns are not processed as bona fide tRNAs". We agree with this notion and thank the reviewer for making us aware of the points he mentioned. It is true, that we did not examine other cell types for the presence of processed nimtRNAs from either endogenous or exogeneous sources. We therefore adapted the text to reflect this by changing the heading of the section to "NimtRNAs located in introns of nuclear encoded pre-mRNAs are not processed as bona fide tRNAs in 293T cells" (line 274).

I thank the authors for the clarification.

Reviewer #1: The section that begins on line 259 describes a series of experiments involving microRNA miR-124. The authors claim that this microRNA has brain-specific expression and therefore is a good choice for use with HEK293 cells. This is incorrect because in humans miR-124 appears in multiple healthy and disease tissues. See for example: http://www.mirbase.org/cgi-bin/get_read.pl?acc=MI0000445 where it is shown to be present in skin, cervix, lung, tonsil, kidney (sample SRR553575 mentioned on the mirbase page), etc. The presence of miR-124 in human kidneys puts into question the appropriateness of using HEK293 cells for these experiments.

Reply: Reviewer #1 noted that microRNA miR-124 is also expressed in other tissues besides brain tissue, including the kidney and is therefore not suitable for the experimental setup. In our approach, we used the microRNA miR-124a from mouse as part of the splicing reporter for transient transfection into human embryonic kidney (HEK293T) cells. The respective probe was additionally designed to be specific for mouse miR124a-1 (specifically targeting differences of the mouse miRNA compared to the human miRNA) to avoid cross-hybridization. We thereby only wanted to determine, if the splicing reporter employed hinders canonical ncRNA processing, which it did not.

As can be verified by looking in miRBase, the sequences of the human and mouse miR-124 are identical:

aggccucucucuccguguucacagcggaccuugauuuaaauguccauacaauuaaggcacgcggugaaugccaagaauggggcug hsa-mir-124-1

aggccucucucuccguguucacagcggaccuugauuuaaauguccauacaauuaaggcacgcggugaaugccaagaauggggcug mmu-mir-124-1

How could the authors have designed a probe that targets "differences" between the mouse and human miR-124 if the sequences are identical?

Reviewer #1: I find the experiments in the section that starts on line 298 confusing. Since the authors weakened the strength of the 5 ́ splice site shouldn't they be obtaining less spliced product compared to the premRNA? Figure 3C says that they obtain more spliced product compared to the premRNA which I do not understand. Did the authors check the length of the spliced product after weakening the splice site to confirm that it did not contain any new segment from the 2.2kb intron?

Reply: The reviewer mentioned that he found "the experiments in the section that starts on line 298 confusing" including the less efficient splicing reporter and the quantification of mRNA, pre-mRNA and total reporter transcript levels. We agree with this notion and want to

clarify our point. As the reviewer assumed correctly, the less efficient splicing reporter indeed results in less spliced transcript (about 450-fold less when comparing wt reporters, but "only" 110-fold when comparing the two different nimtRNA-containing reporters). In the first version of Fig. 3 (now Fig. 3F) we showed the relative fold-change of each type of transcript (mRNA, pre-mRNA total) in the respective reporter (Low0 and Low2 i.e. efficient and inefficient) upon nimtRNA presence i.e. Low0-YCNAW/Low0-wt and Low2 YCNAW/Low2-wt. To make our point clearer we adapted the figure in terms of comparing transcript levels and not ratios. The manuscript was adapted as follows: "As expected, the Low2 wt reporter resulted in an extensive, i.e. 450-fold, decrease in reporter mRNA abundance, compared to the more efficient Low0 wt reporter, while only an about 2-fold reduction in pre-mRNA and total transcript levels was observed. Upon nimtRNA insertion into the Low2 reporter mRNA, spliced mRNA abundance was about 38-fold lower compared to the Low0 wt construct. Thus, insertion of the YCNAW nimtRNA cluster resulted in an about 13-fold increase in spliced mRNA abundance in the inefficient Low2 splicing reporter, thus exhibiting a more pronounced effect on splicing than the efficient Low0 splicing reporter (showing an about 3-fold increase in mRNA abundance).

Notably, pre-mRNA and total reporter transcript levels remained unchanged (Fig. 3F). This can be explained by reporter pre-mRNA being significantly more abundant than spliced mRNA." (starting line 358) The reviewer also asked for confirmation that the inefficient splicing reporter uses the same splice sites/does "not contain any new segment from the 2.2kb intron". Unfortunately, it was not possible to detect reporter mRNA abundance by northern blot analysis due to the 110-450-fold decrease in mRNA abundance compared to the default Low0 wt reporter. Nevertheless, the Low2-eGFP splicing reporter was been validated and previously published {Erkelenz, 2018 #1056}.

I thank the authors for the clarification.

Reviewer #1: The experiments of the section that begins on line 321 use the corresponding tRNA lookalike sequences out of context. To convincingly show the role of these insertions the authors should introduce a sequence that is as long as the mitochondrial genome segment that spans the seven-lookalike cluster and scramble six of the seven lookalikes each time leaving the seventh one untouched. However, even if successful, the experiments would not address the role of the exact mitochondrial tRNA lookalikes that are found outside introns.

Reply: The reviewer notes that we "use the corresponding tRNA lookalike sequences out of context". We agree with his notion, but believe that this imitates what happens during the integration of numtDNA into the introns of nuclear genes, where we find different numbers of introns to be present. For a more detailed explanation as to why we believe our data recapitulates the effects of endogenous nimtRNAs, we refer to the 5 points mentioned above. We want to underline that the role of MTLs outside of introns is beyond the scope of this study.

As I mentioned already, I do not find the five points above to be supporting the hypothesis and wish to reiterate my request for the above experiment.

Reviewer #1: The description of the LC-MS experiments suggests that there was only a single replicate. Given that these are exploratory experiments they should be done in triplicate including controls to increase confidence in the obtained results. Especially, since the proteins that differ in the two MS experiments were used to build networks (Fig. 7). The need for more LC-MS experiments is highlighted by the fact that the authors list PTBP1, U2AF2, KHDRBS1, SF3A3 and UTP18 as enriched compared to control in that replicate. Of these five RBPs, four (PTBP1, U2AF2, KHDRBS1, SF3A3) have eCLIP data available but only one of them supports the LC-MS picture (KHDRBS1 / Suppl File S5). This suggests that the LC-MS has a high rate of false positives.

Reply: Reviewer #1 made us aware of the replicate issue regarding the MS analysis. We agree with the notion that replicates are needed to validate the fold-change of proteins when comparing the two conditions. We want to emphasize that the MS data and inherent analyses were our first indications that the proteins might be of interest for nimtRNA-mediated splicing increase. Our subsequent analyses looked for an overlap (eCLIP) and also determined a single protein binding to a single nimtRNA in a structure-dependent manner (EMSA). Since we were not able to replicate the MS experiments within the time of revision, we adapted our manuscript by only taking into consideration the proteins we found binding to the nimtRNA-containing transcript, where we cut out the bands and did MS analysis. We used this pool of proteins for a subsequent GO analysis which recapitulated our results from before, showing a network of splicing-related proteins binding to the nimtRNA-containing transcript. We go on by looking for an overlap with the publicly available and bioinformatically analyzed eCLIP data and determine KHDRBS1 to bind to a single nimtRNA in a structure-dependent manner. The adaption can be found in the manuscript starting at line 492 and is depicted in Fig. 6A, B and C.

I thank the authors for the clarification.

Reviewer #1: Another complication with the presented evidence of RBP binding the tRNA lookalikes is that there is no mention of the difference in signal between the RBP eCLIP and the corresponding IgG eCLIP from the same cell line (the eCLIP collection includes matching control IgG experiments for each RBP experiment and for the same cell line). Did the authors check that there is no IgG binding at the sites that emerge from the analysis? Which of the enrichments shown in Supp File S5 would survive if IgG data was taken into account?

Reply: We thank the reviewer for raising this question, as it clearly highlights one of the major advantages of eCLIP data in comparison to standard CLIP procedures without an IgG control. However, as we are aware of this fact we already included processed peaks derived from the eCLIP pipeline which only cover regions with a significant signal over control ratio after stringent quality control and thus report only RBP overlaps with our data where binding is likely, which is true for all enrichments listed in Supp File S5. To make this clearer we adapted the methods section accordingly. (These binding sites are already quality controlled and show enrichment after normalization against IgG background, for more information on data generation and processing please refer to https://www.encodeproject.org/eclip/)

I understand the point about the eCLIP pipeline reporting processed results. Looking at Supp File S5 I see that the observed enrichment is very low including for KHDRBS1. So how can the authors claim that eCLIP provides corroborating evidence? Are the numbers reported in File S5 processed results? If so, the authors should report the original eCLIP values for the mitochondrial lookalikes of interest.

I also note something else that is an issue. As can be seen from File S5 the RBPs that appear to bind the lookalikes (albeit with very low enrichment) are different in HepG2 and K562 cells. This means that there is a dependence on cell line. Is this dependence because the mRNAs whose introns contain the lookalikes are not expressed in one of the two cell lines or the mRNAs are expressed but the RBPs are absent or do not bind the corresponding location? Either way, given this cell type dependence which is highlighted by File S5 for binding the lookalikes I do not think that the authors can use the binding of a location by an RBP in one cell line to support binding of the same RBP in another cell line.

Reviewer #1: In the same section that describes the LC-MS experiment, there is mention of mitochondrial tRNA lookalikes. These are presumably human lookalikes. But which ones? Where are they located? What is their sequence conservation compared to the true mitochondrial tRNAs? (See above about the presence of the mitochondrial segment that includes the seven tRNAs in non-intronic regions of the human genome.)

Reply: We want to reply to the comment that the nimtRNAs mentioned in the LC-MS were not properly introduced. In fact, the nimtRNAs employed were identical to those used for the experiments mentioned before, i.e. the YCNAW cluster of five nimtRNAs from mouse Myo3a intronic locus, which were already described in the manuscript. This is mentioned in the manuscript as follows: " This approach utilized a biotinylated T7 transcript containing five nimtRNAs identical to those employed previously (i.e. YCNAW, see above) which was incubated with a nuclear extract generated from HEK 293T cells." (line 498)

I thank the authors for the clarification. This is a very important point that I missed in the first review. By introducing the five-tRNA-lookalike region that is located in mouse Myo3a in human cell lines the authors make the assumption that the same machinery is operational in human and mouse and with the same binding sites. Specifically, that the RBPs that bind this region to induce splicing in Myo3a in mouse exist and operate in exactly in the same manner in human cells. First, it is known that alternative splicing is a tissue specific process; see Yeo et al Genome Biology 2004. Second RBP expression is also tissue specific (see Baralle et al Nat Rev Mol Cell Biol. 2017 and also File S5 of this submission for evidence of same). By using human HEK293 cells the authors assume that the alternative splicing of Myo3a occurs in mouse kidney but they have not provided any evidence for that. Third, even if the alternative transcript of Myo3a was expressed in mouse kidney how do we know that it would be bound by the mouse counterparts of the human RBPs that emerged from the analysis of the human eCLIP data? Do the authors have any evidence to support this? Such evidence is necessary because we know from the work of Odom et al Nature Genetics 2007 that even for conserved transcription factors with tissue specific expression their binding sites are largely organism-specific and differ between human and mouse.

Given these dependencies, in my opinion, the nuclear extract should have been from a mouse tissue where the alternatively spliced products are present (and not from HEK293 cells) and incubation with nuclear extract from a mouse tissue in which the alternative isoform is absent should serve as control. Running the LC-MS in triplicate would have then shown whether there are any endogenous mouse RBPs that bind the region in question in one preparation but not in the other. Intersecting the LC-MS triplicates would have revealed the identity of the relevant RBPs and would have helped determine whether the authors' hypothesis is supported.

Reviewer #1: Finally, the eCLIP data analysis puts further into question the authors' very hypothesis. From the analyzed RBPs only three show enrichment at the mitochondrial tRNA lookalike sites: DHX30, G3BP1, and KHDRBS1. DHX30 is a component of the mitochondrial nucleoid and associates with mitochondrial DNA. If the mitochondrial tRNA lookalikes are exact copies of the mitochondrial tRNAs (as is the case of the lookalikes in the analyzed mouse cluster) it appears more likely that DHX30 is binding to the mitochondrial tRNA itself and not the intronic instance in the nuclear genome as the authors claim. The second RBP, G3BP1 is not known to be involved in splicing. Only the third RBP, KHDRBS1, is known to be involved in splicing but that is not its only role. Moreover the splicing connection hinges upon the assumption that the mitochondrial tRNA lookalike that is bound to KHDRBS1 is not expressed as a bona fide tRNA but only as part of an intron: this was not demonstrated for the human mitochondrial tRNA lookalikes that were analyzed by eCLIP, and, of course, still does not address the matter of the lookalikes that exist outside introns.

Reply: Please take into account that the enrichment we in show in Supp File S5 is adding an additional layer of information. One comes directly from eCLIP peaks, which, as stated above, are only called? if a significant enrichment of RBP binding over IgG control can be

found for the region of interest. Additionally, we calculate the relative enrichment of RBP overlap with our data over background from binding sites in intronic regions and list those values in Supp File S5. The three mentioned RBPs are therefore not the only ones with significant peaks that overlap, but in addition they bind our sites more often than expected in comparison to other intronic regions.

We agree that the biological function of these interactions is so far not well characterized and as the reviewer points out we can only draw conclusions from already published functions of these RBPs, as investigations into this matter beyond the experiments already conducted require dedicated studies which are outside the scope of this manuscript. We agree with the reviewer that there are no indications that DHX30 is present in the nucleus and thus we excluded it from our results. Not much is known about the role of G3BP1 is in general, other than its ability to interact with RNA and DNA and its unwinding capabilities for duplexes of RNA/DNA and RNA/RNA, but it has been found in the nuclear compartment, which is why we did not exclude it.

As I mentioned above, File S5 does not show any enrichment that supports binding (the reported enrichment values are very low and no associated P-values are shown). More to the point, since the authors analyzed eCLIP, and despite the human/mouse incongruence they should be able to show the original raw human data: where exactly along the various tRNA lookalikes do the shown RBPs bind? where along the length of a tRNA is the binding motif? if all five of the tRNA lookalikes can lead to splicing it must mean that the binding motif should exist in all five tRNAs: so, is it a sequence motif or a structural motif? if the motif exists in the introns of other mRNAs and there is no eCLIP evidence of binding what would the implication be for the claimed role of the tRNA lookalikes in splicing?

Reviewer #1 has stated that "the eCLIP data analysis puts further into question the authors' very hypothesis" referring to the sparsity of overlap between eCLIP and MS analyses.

Reply: We agree that it is impossible to exclusively map binding sites to either the mitochondrial tRNA or the intronic counterpart if they are exact sequence copies and no flanking sequence is taken into account. This is of course true for all regions in the genome which harbour identical or near identical sequences. As many RBPs are, however, known for their sequence specific binding preferences, binding to both sequences is equally likely, given that both RBP and target are available for binding which is the case here.

Do the authors have evidence to support the implicit claim that the sequence-specific binding applies to the RBPs that they singled out in their analyses?

Furthermore, the downloaded eCLIP peaks undergo stringent quality control, including removing of mappings to repeat regions and minimum coverage of peak regions by uniquely mappable reads as stated in https://www.encodeproject.org/documents/739ca190-8d43-4a68-90ce-1a0ddfffc6fd/@@download/attachment/eCLIP_analysisSOP_v2.2.pdf.

See my point above about the claimed enrichments.

Moreover the splicing connection hinges upon the assumption that the mitochondrial tRNA lookalike that is bound to KHDRBS1 is not expressed as a bona fide tRNA but only as part of an intron: this was not demonstrated for the human mitochondrial tRNA lookalikes that were analyzed by eCLIP, and, of course, still does not address the matter of the lookalikes that exist outside introns.

Reply: We thank the reviewer for bringing up this issue. We took this into account which is why we included an additional 10 nucleotides up- and downstream of the nimtRNATyr from the endogenous Myo3a locus in mouse in the transcript used for the electrophoretic mobility shift assay. This is now included in the text as follows: "To this end, a transcript of nimtRNATyr including an extra 10 nucleotides at the 5' and 3' termini, in order to not resemble processed tRNA ends, was radioactively labeled and incubated with increasing concentrations of PTBP1 protein." (Line 518)

As I mentioned above the presence of the lookalikes outside introns needs an explanation. If inserting the five lookalike region into a reporter assay is sufficient to cause alternative splicing it is not much of a stretch to expect that insertion of the a tRNA lookalike in regions that are in intergenic space and are transcribed could control the products from those regions. The authors find some of the lookalikes in lncRNAs: is there evidence of alternative splicing for these lncRNAs? is there evidence of binding for the RBPs of interest to those regions? These are relevant questions that have not been addressed.

**Reviewer 2**

The authors have answered my comments in a well-justified and satisfactory manner. The manuscript is clearer with the modifications and constitutes an important contribution to the literature.

**Authors Response**

**Point-by-point responses to the reviewers’ comments:**

Reviewer #1: 
I thank the authors for their detailed answers. Below I list the items that remain outstanding.

Reviewer #1: Telonis et al. labeled the sequences they discovered "tRNA-lookalikes" and the term has since been used in multiple papers. 
Even though the authors reference the paper, they ignore the earlier work and relabel the same sequences as "nuclear encoded mitochondrial 
tRNAs (nmtRNAs)". Instead the authors should use the original term "mitochondrial tRNA lookalikes" in order to put their work and new 
contributions in the proper context.

Reply: We agree with the notion that Telonis et al. previously coined the term tRNA- lookalikes. We therefore changed the term nmtRNAs to 
mitochondrial tRNA-lookalikes (MTLs) in the entire manuscript. Intronic MTL sequences are however in the following referred to as nimtRNAs 
since we link them to a novel biological function.

As a group of sequences/molecules the mitochondrial lookalikes were reported and labeled in 2014. I find the offered justification for renaming them puzzling. Why do the authors insist on renaming a category of sequences, the mitochondrial tRNA lookalikes, that have had a name for six years already?

We feel that the discussion on the mere nomenclature of tRNA lookalikes is redundant. Among all tRNA lookalikes, nimtRNAs are currently the only class with an experimentally validated function; in analogy, there are numerous ncRNAs with identical sizes, such as miRNAs or piRNAs or siRNAs; however, based on their biological function, they are termed differently; hence, we classify tRNA lookalikes within introns as nimtRNAs while still using the term lookalikes for all mtRNA species.

Reviewer #1: On a related note, in the section "Conservation of nmtRNAs and nimtRNAs" the authors discuss sequence conservation. They 
report that they found several MT tRNA lookalikes "that appear to have evolved significantly more slowly than the adjacent numtDNA 
sequences." The authors are again attempting to reinvent the wheel. In both Telonis et al Frontiers Gen 2014 and Telonis et al RNA Biology 
2015, both of which are referenced by the authors, there is explicit discussion about the fact that specific mitochondrial tRNA lookalikes 
are conserved identically in the nuclear genome.

Reply: The reviewer might mix up two distinct issues here: (1) Given the high rate of numtDNA insertion [see e. g. Bensasson et al. 2003, 
et al 2004, Hazkani-Covo 2017], there are of course MTLs that are identical to the contemporary mitochondrial tRNAs of the respective 
species. These are in particular MTLs originating from very recent insertion events. (2) We focus on MTLs that are present in comparably 
old numtDNAs. These have had enough time since their insertion to evolve in the nuclear genome. Therefore, the substitution rates of the 
MTLs and the surrounding numtDNA can be used to quantify selection pressures. We have included a detailed description of the process of 
numtDNA insertion and evolution within the background section (including an additional Figure 1), which we hope helps to clarify this issue.

The Introduction as amended contains a number of statements between lines 118 and 174 about mtDNA insertions in the nuclear genome. Given that these statements try to build a case for the presented work it is important that they be supported by relevant references. The authors should include these references.

We added the references as proposed by the reviewer.

In fact, some of the claims do not agree with known findings. For example on line 124 it is stated that "Insertions appear uniformly across the genome". This is not the case for the tRNA lookalikes. In Telonis et al Frontiers Gen 2014 it was shown that the distribution of the tRNA lookalikes is not uniform across the nuclear genome and that the lookalikes co-localize with the known tRNAs, which themselves have a biased distribution. Also, these insertions are biased in favor of specific isoacceptors (see Fig. 3 of Telonis et al). How do the authors reconcile their claims with the current literature?

The insertion of numtDNA is not uniform random across the genome. We agree that the wording in our manuscript was not sufficiently accurate, and we have changed the text accordingly (starting line 136). However, the reported deviation from uniform random insertion does not affect our argumentation, which only requires the fact that numtDNAs are almost always located far from each other so that their insertions can be regarded as independent events.

In brief, Tsuji et al. 2012 indeed show convincingly that there is a bias of numtDNA insertions to occur close to retrotransposable elements. However, the genomic distribution of retrotransposable elements ``can be explained by a stochastic process of random placement due to retrotranscription followed by genome expansion and sequence duplications.'' [Riba 2019]. Older work [Mestrat et at. 2002] also showed that retro-elements are more abundant in AU-rich regions, in line with the observation that the same is true for numtDNAs [Tsuji et al. 2012]. Mestrat et al. 2002 also show a depletion of retroelements in the close proximity of genes that is explained by the dependence of insertion frequency on GC content, arguing against a specific association of numtDNA insertions with genes. The association with retroelements, which are very abundant compared to numtDNAs, in no way contradicts the fact that numtDNA insertions are *not* clustered in particular genomic areas or chromosomes [Tsuji et al. 2012], or the fact that numtDNA insertions can be regarded as independent random events (albeit with a probability distribution that is not uniform but varies with AU content and proximity to retroelements). Regarding the inserted part of the mito-genome, several previous studies conclude that the fragments are random, except maybe for the small region around the D-loop, i.e., the replication origin of the mitochondrion [Mourier et al. 2001, Tourmen et al. 2002, Hazkani-Covo et al. 2007]. Any bias for isoacceptor classes is thus a direct consequence of the clustering of mt-tRNAs in the mito-genome (in particular the „WANCY“) and irrelevant to our work.

Reviewer #1: On line 223 of this manuscript, the authors also claim that "the insertion of nimtRNA genes in the respective introns of 
nuclear genes might be a very recent evolutionary event, which might have occurred independently in different species in addition to 
potential retainment of pre-existing nimtRNAs." This is not true. The Telonis et al 2015 paper showed that the concept of mitochondrial 
tRNA lookalikes is pervasive across at least 200 My of evolution and that in organisms from primates to marsupials one can find several 
mitochondrial tRNAs that have exact copies in their nuclear genomes.

Reply: This might be a misunderstanding. We do not claim that the process of MTL insertion is a recent evolutionary innovation. On the 
contrary, numtDNA insertion is a rapid process. Most individual numtDNAs and MTLs therefore are the result of recent insertion events. This 
is in particular true for MTLs that are identical to the contemporary mitochondrial tRNAs. We have not been able to trace a single MTL to 
an insertion event that pre-dates common ancestor of human and marsupials. On the other hand, there are examples of MTLs in both lineages. 
In fact, the cited paper (Telonis et al.) also argues that "tRNA lookalikes represent independent evolutionary events in some animals".
Due to the way in which sequence conservation is determined in several publications (among others also in Telonis et al.), conservation 
from genome browsers cannot be used for the conservation analysis of repetitive or multicopy sequences, including tRNAs (see Velandia et 
al. 2016). Conservation is assessed based on genome-wide alignments, which in turn is constructed using anchor-based methods, i.e., 
essentially local (blast) hits. As a consequence, newly inserted numtDNAs match the mitogenome and/or numtDNAs in other species. These 
sequence fragments are then placed into the local alignment blocks. This effect is fairly easy to detect in genome-wide alignments. While a 
deletion is shown for VERY closely related species, more distantly related species appear in the alignment. For example, in the browser 
image shown below, the recently inserted human numtDNA is clearly visible: it is identified as missing in the Rhesus Monkey, Dog and 
Elephant and then suddenly appears again in more distantly related species.

I agree with the methodology that the authors describe here. However it is based on the assumption that the further back in time a NUMT sequence entered the nuclear genome the more divergent it is. Does the assumption hold for the segments of the NUMTs where the tRNA lookalikes are found? Telonis et al 2014 show that the lookalikes are depleted in polymorphisms which suggests evolutionary pressure to maintain their sequences.


Also note that the numtDNA shows "evidence" for expression from EST data: clearly an artefact of mapping data from mitochondrial RNAs.

Here the authors essentially state that the EST mapping to which they refer, presumably drawn from the UC Santa Cruz genome browser, is incorrect: what evidence do the authors have to support this claim? And isn't this claim contradicting the authors' own argument? If the mapping of EST data to numtDNA is an "artefact" shouldn't the mapping of the eCLIP data to numtDNA also be an "artefact"? Yet, the authors use the latter mapping to support their own analyses.

Other comments here: 
a) I note that the authors mention an image in their reply but an image was not included with the document that I received. 
b) Out of curiosity I checked A. thaliana's genome and it contains mitochondrial tRNA lookalikes as well. So it appears that plants do make use of lookalikes even though, for example, the concept is not present in invertebrates.

In our opinion most of the reviewer's comments appear to be misunderstandings on the side of the reviewer. While we attempted in this revision to further clarify the language where possible, we maintain that our analysis is technically correct and fully supports the conclusions drawn in our manuscript. There are two issues that appear repeatedly throughout the comment, which we answer by topic below:

Reviewer #1 repeatedly objects to the fact that we single out a subclass of tRNA-like elements or MTLs for functional analysis. It appears from the comments that the reviewer BELIEVES that all tRNA-lookalikes or all MTLs (1) must have a biological function and (2) this function must be explained by the same molecular mechanism. However, there is absolutely no evidence for this claim. In fact, this hypothesis contradicts all common knowledge on the evolutionary processes shaping genomes. The reviewer's assertion is akin to arguing that ALUs inserted into the vicinity of a gene start cannot act as promoters because there are also ALUs far away from genes that do not act as promoters. Clearly this line of reasoning is flawed. To clarify the issue:

- We have to expect that a newly inserted numtDNA, like any other inserted DNA such as transposable elements (TEs) or retrogenes, has no biological function after insertion, even though it will interact with the machinery in the nucleus - which is very different from the environment in the mitochondrion. For retrogenes, for example, in general there are no promoters, i.e., in general they are not expressed, and other – exapted – function e.g. as binding site on the DNA also will arise only if the inserted element is fortuitously placed into the right context. For TEs and pseudogenes the absence of selective pressures (and thus of function) is evidenced by the fact that such elements are routinely used to estimate neutral rates of evolution.

- In particular, it is clear that a newly inserted numtDNA, including the MTLs and the sequences coding for mitochondrial genes, will have lost their original biological function upon insertion in the nuclear genome.

- MTLs and other inserted elements thus have to gain new functions by exaptation. Otherwise they will be erased by accumulating mutations on a time-scale set by neutral evolution, which in mammals implies that the elements remain recognizable for time-scales up to about 50 Mio years (corresponding to roughly 50% residual sequence similarity, and roughly the detection limit for sequence alignments.) Thus, many partially degraded non-functional insertions are still detectable. Exaptation is a process that requires (a) a molecular mechanism through which the exapted element acts AND (b) a selective advantage for the organism associated with this mechanism. In our work we have shown that some MTLs act to affect splicing. Even if all intronic MTLs can bind to a certain portfolio of splicing-associated proteins there is no reason to assume the effect on splicing has a biological function in all cases. Nor is it plausible to assume that all intron MTLs are amenable to the same molecular mechanisms in vivo, because competing molecular mechanisms (such as overlapping binding sites for other proteins or strong RNA secondary structures) may interfere with the requisite molecular interactions.

In order to conclude that intronic MTLs *can* be exapted to regulate splicing and that their molecular interactions can enhance splicing efficiency it is therefore not necessary to demonstrate these effects for all intronic MTLs. The computational evidence in the manuscript indicates that a few of them have come under negative selection, and thus are likely functional, and the ones with the strongest signal for selection are intronic. It is plausible to hypothesize, therefore, that in addition to the MTLs for which we have experimentally verified an effect on splicing, this is also the case for the intronic MTLs for which we have evidence of selection.

- Conversely, the fact that there are also MTLs outside introns in regions that likely are not expressed at all is not reason to conclude that intronic MTLs do not act at RNA level or that they are not involved in splicing. The existence on non-intronic MTLs has no bearing whatsoever on the function (or lack thereof) of an MTL that is located in an expressed RNA. Whether or not MTLs in promoter or enhancer regions may be exapted to act as transcriptional regulators is besides the point. Our work and our manuscript make no statement or implication either way. In summary, we maintain that our analysis and our interpretation of the data is sound.

Reviewer #1 questions the evidence from different types of publicly available sequence data. As far as we can disentangle the comments, the issues are the following:

- Evidence for expression of tRNA loci in the Telonis paper is taken from ENCODE data tracks that represent short RNA-seq data that were mapped to the genome with the "all best matches" strategy, i.e. a read is placed at ALL positions in the genomic references that gives an alignment with maximal score. (In fact, the Telonis paper does not discuss this issue at all, we took from track name shown in the figures which data were used there.) That is, in particular, a read perfectly matching a mitochondrial tRNA will also be mapped to *all* identical numtDNA sequences in the nuclear genome. (Due to mismatches e.g. incurred from reverse transcription across chemical modifications (which are abundant in tRNAs including mt-tRNAs) many mt-tRNAs reads will not identically match the mt-DNA and thus even more readily map multiple sites in the nuclear genome.) We therefore argued in our reply that these data cannot be used as unambiguous evidence for the expression of tRNA-like RNAs from MTL loci. We stand by this statement in our reply to the reviewer's previous comments.

We do not discuss this issue in the manuscript in any detail, and we do not claim that NO MTL is expressed and processed into a tRNA-like ncRNA. We only state that his not the case for those intronic MTLs for which we have tested this possibility explicitly and obtained a negative answer. Whether or not some MTLs are expressed and processed has no bearing on our data or their interpretation.

- We do not quite understand the reviewer's comment regarding EST data since we did not argue about ESTs anywhere in our work. We *suspect* that the comment refers to an argument for expression of tRNA-lookalikes in the literature that the reviewers sees as contradicting our results. The issues of mapping uniqueness discussed in the previous paragraphs also pertain to EST data. In the *current version of the UCSC browser*, all alignments of EST to the reference genome are shown that have "a base identity level within 0.5% of the best and at least 96% base identity with the genomic sequence" [Description of the UCSC track "Human ESTs Including Unspliced" for GRCh38/hg38]. As a consequence, mitochondrial sequences will also match very recently inserted numtDNAs that are still nearly identical to the mitogenomic sequence. Similarly, the browser tracks do not distinguish expression between recent tandem duplications or retrogenes and the original (spliced) gene. Therefore, ESTs shown at loci with highly similar duplicates elsewhere in the genome (including the mito-genome, and ESTs that exclusively cover numtDNA sequence) cannot be interpreted as conclusive evidence for expression from a given locus without further, careful analysis. Again, we make no statement as to whether or not all numtDNAs are expressed. We are only concerned with the ones that are included in introns and therefore in transcripts that also contain unique sequence. For these, expression of the intronic MTLs (in fact the entire numtDNA) is proved by the expression of the exonic part of the transcript. In our opinion, the issue of ESTs shown in genome browser tracks is at most tangentially related to our work, if at all, and therefore does not warrant a discussion in our manuscript.

- The eClip data used in our study are also taken from ENCODE resources. These data were produced by the ENCODE Consortium with the "unique-best match" strategy, i.e., only reads that show a unique best match in the genome are mapped at all. All reads with multiple equivalent best matches were disregarded completely. The authors of the ENCODE study then processed foreground signal and background control to determine significant eClip peaks from these data. Since (1) eClip reads are much longer than the short-read data mentioned in the previous point and (2) care was taken by ENCODE authors to use only uniquely mapping reads, we deem their data safe to use for our purposes.

Reviewer #1: Many of the experiments that the authors describe involve a cluster of five mitochondrial tRNA lookalikes from intron 30 of 
Myo3a. In actuality there are seven such tRNAs and not five (the coordinates of all seven are in the manuscript's supplemental material). 
First, all seven of these mitochondrial tRNA lookalikes were reported in Telonis et al 2015 (see 
https://cm.jefferson.edu/data-tools-downloads/trna- lookalikes-beyond-homo-sapiens/), and are not new discoveries.

Reply: We agree with the comment that the nimtRNAs from the mouse Myo3a cluster were previously reported which we now included into the 
text as follows: "A cluster of five nimtRNA genes (out of the seven nimtRNAs of the Myo3a gene, Fig. 3A), previously reported to be present 
within intron 30 of the mouse Myo3a gene (35), was inserted into the intronic region of this splicing reporter." (line 281). Also, we show 
in Figure 3A that there are a total of seven nimtRNAs within the Myo3A intronic locus.

On a related note, lines 189-192 state "Applying the computational annotation workflow presented here, we were able to identify numerous novel MTLs and nuclear encoded intronic mitochondrial-derived tRNA genes (designated as nimtRNAs) in humans and mice. Notably, nimtRNAs were always flanked by sequences of mitochondrial origin." Looking at the Excel of File S1 I see more than one thousand tRNA lookalike entries and many claimed NUMT regions. I could not find a caption for the file and it is not clear what it describes. A detailed description is needed.

To clarify this issue, we modified the column names of File S1 and S2.

In addition, the authors should mark as separate columns which of the NUMT regions listed here are already known in the literature. Specifically for the mitochondrial lookalikes contained in these NUMT regions, they should mark in separate columns those that were previously reported in Telonis et al 2014. 

Doesn't Infernal/tRNAscan provide a quality score and probability values for the matches? If so the authors should add these as separate columns.

We added the scores of Infernal and tRNAscan-SE to the tables S1 and S2.

This is an important point because I am not convinced that all of the reported mitochondrial lookalikes that appear in intronic regions (File S1) are correct. For example, I selected randomly ten among the shown intronic lookalike sequences and searched the genome with BLAT: half of them are problematic which raises an important concern. For example,

- ATAGTTTAACCAAAACATTAGATTGTGAATCTAACAATAGAAGCCTGCAACTTCTTGT in the intron of ENSG00000198216 is 58 nt long but nucleotides 38 through 58 are not present in any MT tRNA as they should
- GTTAAGAAGAGGAGTTGAAATTCTGATTATAAAGTTTTAGGTTTTATGCAATTGCTGGGCTCTGCCATCTTAAC in the intron of ENSG00000204740 is 74 nt long but nucleotides 1 through 29 are not present in the best matching MT tRNA as they should
- AGATATTAGTAAAATTATTTCCTAACTTTGCCAAAGTTAAGTTATAAGTTAAACCCTGTATATCTTA in the intron of gene ENSG00000185352 is 67 nt long but there is no evident alignment to any of the 22 MT tRNAs 
- GTAAATAAGAAGTTGCGGACTTCTATTATTAGACCACAATCTAACATTTTGATTAAACTATATTTAC in the intron of gene ENSG00000003400 is 67 nt long but there is no evident alignment to any of the 22 MT tRNAs
- AAAGTGTCGCTTAACCCAAAGCATCCAGCTTACACCCGGAAGATTTCATCACAACCTGGTCACTTTGA in the intron of gene ENSG00000096968 is 68 nt long but there is no evident alignment to any of the 22 MT tRNAs

Perhaps the other, non-intronic, novel sequences shown in this file include problematic sequences too. If sequences were to be removed following manual inspection, then the reported findings that are based on the results of File S1 would be affected, as would the claims based on those findings.

We do not consider incomplete sequences (such the first two sequences in the report or reviewer 1) problematic. They still constitute homologs of mt tRNAs and thus are included in the list. For the other example, we cannot reproduce the reviewers claim that they are not related to mitochondrial tRNAs. We checked the sequences using blast the Sprinzl-tRNA-Database. They yield very good alignments to mt-tRNAs.  As best alignments for the third sequences in Reviewer 1’s report we obtained:

Query: 1 agatattagtaaaattatttcctaactttgccaaagttaagttataagttaaaccctgtatatctta 67

Sbjct: 2 agatattagaaaaactatttcataactttgtcaaagttaagttacgggttaaaccccgtatatctta 68

where Sbjct is the *Gorilla gorilla* mt-tRNA-Asp-GTC. For the next case the best alignment is with the *Macaca arctoides* mt-tRNA-His-GTG as follows:

Query: 1  gtaaataagaagttgcggacttctattattagac-cacaatctaacattttgattaaactatatttac 67

Sbjct: 68 gtaaataagaagtggtgagcttctatagttagactcacaatctagtgttttgattaaactatatttac 1

Again, this is a near perfect alignment. Since the literature contains evidence that reverse complements of tRNAs have tRNA-like properties, we find it entirely reasonable not to exclude reverse complements out of hand, in particular since our work provides evidence that the effect on splicing is linked to the structure of MTL element.

Reviewer #1: Second, there is a methodological problem here as the authors arbitrarily dismiss two of the seven lookalikes. This is 
problematic in two ways. If the authors' hypothesis is that these intron-embedded lookalikes are functional then all seven sequences should 
be tested in the experiments and not only five. These seven tRNA lookalikes in intron 30 of Myo3a are in two groups: two tRNA lookalikes 
followed by a pseudo-copy of MT-CO1 followed by the other five tRNA lookalikes. This is the same arrangement one finds in the mitochondrial 
genome.

Reply: Reviewer #1 notes that we only used five of the seven nimtRNAs present within this intron. We here list five reasons why we chose 
our approach in this manner and why we maintain that our data is valid:
1.A longer insert would hinder cloning approaches and the reporter splicing assay:
The five nimtRNAs used are clustered together closely and span a region of only 500 bp while the other two are 1.5 kb upstream which 
hindered reporter cloning. Furthermore, we needed to compare the nimtRNA-containing splicing reporter to the wt reporter and a bigger 
difference in intron length could have skewed the results.
2."5 nimtRNAs were sufficient to produce a strong effect:
We observed that the insertion of the mentioned quintet of nimtRNAs increased mRNA abundance by almost 4-fold; thus a strong splicing 
effect was already observed when employing the respective five nimtRNAs, only.
3.Effect was nimtRNA-number-dependent:
We observed that the increase in mRNA abundance was nimtRNA number-dependent. Thus, we assumed a further increase of nimtRNA numbers to 
potentially increase the effects, but not to change the type of the splicing effect.
4.All nimtRNAs showed effects on their own:
We observed the same type of effect in different degrees of prominence for all the different nimtRNAs tested. Therefore, we do not expect a 
different type of effect for the two nimtRNAs upstream of the cluster of five nimtRNAs.
5.Intronic nimtRNAs come in different combinations:
NimtRNAs are found either as singles or in different combinations within the introns of their host genes. In terms of the "YCNAW" cluster 
used in our approach, the human CENPP gene contains exactly this combination. In the human AKAP6 gene the W is missing, in the JAK2 gene a 
total of 18 nimtRNAs can be found including the whole YCNAW cluster. We therefore suppose that the inclusion of two extra nimtRNAs would 
not qualitatively affect results.
We thus believe that the results obtained with the "YCNAW" cluster recapitulate in an exemplary manner the effect of intronic nimtRNAs and 
combinations thereof in splicing regulation.

I understand the technical problem with the reporter cloning (Reason #1). However, Reason #3 is an assumption and no data has been shown to support it. Reason #5 is simply speculation and is not supported by any presented data either. Reason #4 suggests that the two lookalikes that have not been tested could be functional. To state that the authors "do not expect a different type of effect" is not adequate and supporting data should be provided. The authors should test at least one and ideally both of the remaining lookalikes for their ability to affect splicing. Finally, Reason #2 is not sufficient by itself. If the remaining two lookalikes do not affect splicing then wouldn't that contradict the authors' conjecture about the role of the intronic tRNA lookalikes?  

Based on the data so far, the authors showed for a single instance of an intronic embedding that some of the tRNA lookalikes affect splicing. The sequences are present in the introns of a mouse gene but tested in a human cell line, a choice that introduces new and unknown variables that I discuss below. Then the authors use the fact that similar sequence embeddings exist in the introns of other genes to extrapolate and conclude that all such embeddings affect splicing. I think that this is quite a leap. It is fine to conjecture it in the Discussion but in my opinion the authors have not presented adequate data to allow the generalization mentioned in the title of the manuscript and in the text.

We do not agree with the reviewer on that matter. In particular, Reason # 3 is not only an assumption, since by experimental evidence we show a nimtRNA number-dependent effect on splicing in the constitutive splicing reporter. Also, Reason #5 is not pure speculation but a reasonable hypothesis supported by experimental evidence, since we observed the same effect for all different nimtRNAs and *bona fide* mtRNAs investigated. Notably, we show the effect of nimtRNAs on splicing in two constitutive splicing reporters and one alternative splicing reporter. Importantly, we also were able to demonstrate the same effect of a nimtRNA on splicing within an endogenous intronic locus by a CRISPR knock-out; an additional nimtRNA for which we had no previous evidence in our splicing reporter assays. We thus reemphasize our hypothesis of nimtRNAs increasing splicing efficiency.

Reviewer #1: If one searches the mouse genome with this mitochondrial segment (2 tRNAs plus MT-CO1 plus 5 tRNAs) one finds matches in the 
nuclear genome for both the full segment and the individual tRNAs. Many of these copies hit regions that do not contain any known 
transcripts and are exact copies of the mitochondrial tRNA. If the hypothesis put forth is correct shouldn't all of these lookalikes be in 
introns, either by themselves or as groups? I do not see how the hypothesis is warranted if there are comparable numbers of copies in 
intronic and in non-intronic regions of the genome.

Reply: Reviewer #1 questioned how the presence of intergenic, non-intronic nimtRNAs is in accordance with our hypothesis of 
nimtRNA-mediated splicing regulation. In the experimental part of this study, we focused on investigating the specific effects of nimtRNAs 
within the introns of genes and did not investigate the biological relevance of intergenic MTLs. The potential biological relevance of 
intergenic MTLs is clearly outside the scope of this study.

I maintain that this point is relevant and goes to the heart of the authors' claim. The authors report 731 mitochondrial tRNA lookalikes in the human genome of which half are novel. Of these, 109 are present in the introns of 29 protein coding genes as per File S1. For one of the genes the authors showed that these embeddings are relevant for splicing. Even if this holds true for the other 28 genes, which has not been shown, there is still the matter of 622 lookalikes that are not inside introns. If they are non-functional then one would be inclined to conclude that the intronic embeddings were a random event in the first place and were subsequently co-opted to a new function (modulation of splicing). But as we already discussed the embeddings of the lookalikes in the human genome are not random. If they are not random, one would expect that the same sequence would have the same function independent of where in the genome it was embedded. This would in turn suggest that the role of the embeddings should be the same for both the intronic and intergenic embeddings. However, the proposed function (splicing) is claimed to be relevant only for the intronic subset. How do the authors reconcile these observations? To simply state that they choose to not investigate the role of the intergenic copies is not adequate. I will discuss this point again below.

As in our previous reply, we do agree with reviewer 1 that it would be interesting to determine the biological relevance of intergenic mitochondrial tRNA-lookalikes. However, we reiterate that this topic is outside of the scope of the current study.

Reviewer #1: Also, if the nuclear copies of this mitochondrial segment of 2 tRNAs plus MT-CO1 plus 5 tRNAs have a role in splicing in 
mouse, it is reasonable to assume that this is the case in the human genome as well. Here, a search reveals that the human orthologue of 
the mouse segment has several nearly full-length copies at different locations of the human nuclear genome that are not in the introns of 
any genes. This finding further questions the hypothesis that the mitochondrial tRNA lookalikes play a role in splicing.

Reply: We thank the reviewer for this comment and agree with the notion of it being likely that the quintet of nimtRNAs does also have a 
role in the human genome. He/she mentions that "several nearly full-length copies at different locations of the human nuclear genome that 
are not in the introns of any genes". He goes on to say that this "questions the hypothesis that the mitochondrial tRNA lookalikes play a 
role in splicing". We propose that nimtRNAs are not exclusively found within intronic regions but also within intergenic regions 
(designated as MTLs), because MTLs, and numtDNA in general, are integrated into the nuclear genome by chance and not in a targeted manner. 
In most cases it is likely that intronic integration of numtDNA is detrimental to gene expression/function and cellular metabolism and will 
therefore be actively selected against. Integration events occurring in intergenic regions on the other hand, would be less likely to be 
actively selected against as one would predict they are less likely to have a negative impact on cell function and survival. We do not 
exclude that nimtRNAs may have additional functions outside of introns, which might affect their conservation.

Here the authors base their argument on the assumption that the tRNA lookalike embeddings occur randomly in the genome. As we saw above this is not the case. The point remains: if these embeddings occur both in intergenic and intronic regions one cannot interpret the intronic embeddings in isolation. Especially when the exact intronic embedding with which the authors experimented in mouse, is present outside introns in humans (and I would imagine in mouse too). 

See our detailed answer above. The literature agrees that the insertion of numtDNAs is a random process that shows a bias towards insertions in A/T rich regions and for insertion sites close to retrotransposable elements. The usual mechanism of numtDNA insertion appears to be non-homologous end joining repair (Ricchetti et al 1999, Woischnik et al 2002, Hazkani-Covo et 2008, an others). There is no evidence whatsoever that numtDNA insertion has any relationship to genomic location beyond the moderate sequence bias for the insertion sites.

It appears that the reviewer believes that numtDNA insertion itself serves a particular purpose, and even more, serves a single purpose. This contradicts the well-established paradigm that evolution proceeds by the purpose-free generation of genetic variation that is then evaluated for its fitness through natural selection.

Reviewer #1: The section that begins on line 259 describes a series of experiments involving microRNA miR-124. The authors claim that this 
microRNA has brain-specific expression and therefore is a good choice for use with HEK293 cells. This is incorrect because in humans 
miR-124 appears in multiple healthy and disease tissues. See for example: http://www.mirbase.org/cgi-bin/get_read.pl?acc=MI0000445 where it 
is shown to be present in skin, cervix, lung, tonsil, kidney (sample SRR553575 mentioned on the mirbase page), etc. The presence of miR-124 
in human kidneys puts into question the appropriateness of using HEK293 cells for these experiments.

Reply: Reviewer #1 noted that microRNA miR-124 is also expressed in other tissues besides brain tissue, including the kidney and is 
therefore not suitable for the experimental setup. In our approach, we used the microRNA miR-124a from mouse as part of the splicing 
reporter for transient transfection into human embryonic kidney (HEK293T) cells. The respective probe was additionally designed to be 
specific for mouse miR124a-1 (specifically targeting differences of the mouse miRNA compared to the human miRNA) to avoid 
cross-hybridization. We thereby only wanted to determine, if the splicing reporter employed hinders canonical ncRNA processing, which it 
did not.

As can be verified by looking in miRBase, the sequences of the human and mouse miR-124 are identical:

aggccucucucuccguguucacagcggaccuugauuuaaauguccauacaauuaaggcacgcggugaaugccaagaauggggcug hsa-mir-124-1
aggccucucucuccguguucacagcggaccuugauuuaaauguccauacaauuaaggcacgcggugaaugccaagaauggggcug mmu-mir-124-1

How could the authors have designed a probe that targets "differences" between the mouse and human miR-124 if the sequences are identical?

Since there are different gene copies of miR-124 reported in human and mouse, upon closer inspection, there are in fact two identical miR-124 sequences reported for human and mouse genomes. We thus thank the reviewer for making us aware of this. However, this does not disprove our point. In a time course experiment, we observed no miR-124 expression signal one hour after transfection. Thus, no endogenous microRNA signal impedes the interpretation of our results. Furthermore, by northern blot analysis Quintana-Lagos et al previously showed miR-124 to be strictly brain-specific (Quintana-Lagos, Current Biology, 2002) in mouse; we do not exclude that by more sensitive methods (qPCR , RNAseq) miRNA-124 can be detected also in other tissues than brain, however by the method employed in our study (i.e northern blot) no signal for miRNA-124 has so far been reported. We adapted the manuscript accordingly: “To that end, we cloned the genes for two brain-specific ncRNAs not expressed in HEK 293T cells, i.e. mouse miR-124a and C/D box snoRNA SNORD115, respectively, including flanking regions into the identical intronic location. “ (ine 322)

Reviewer #1: The experiments of the section that begins on line 321 use the corresponding tRNA lookalike sequences out of context. To 
convincingly show the role of these insertions the authors should introduce a sequence that is as long as the mitochondrial genome segment 
that spans the seven-lookalike cluster and scramble six of the seven lookalikes each time leaving the seventh one untouched. However, even 
if successful, the experiments would not address the role of the exact mitochondrial tRNA lookalikes that are found outside introns.

Reply: The reviewer notes that we "use the corresponding tRNA lookalike sequences out of context". We agree with his notion, but believe 
that this imitates what happens during the integration of numtDNA into the introns of nuclear genes, where we find different numbers of 
introns to be present. For a more detailed explanation as to why we believe our data recapitulates the effects of endogenous nimtRNAs, we 
refer to the 5 points mentioned above. We want to underline that the role of MTLs outside of introns is beyond the scope of this study.

As I mentioned already, I do not find the five points above to be supporting the hypothesis and wish to reiterate my request for the above experiment.

We disagree with the reviewer and refer to our reply above; in addition, this experiment has not been requested in the editorial review process.

Reviewer #1: Another complication with the presented evidence of RBP binding the tRNA lookalikes is that there is no mention of the 
difference in signal between the RBP eCLIP and the corresponding IgG eCLIP from the same cell line (the eCLIP collection includes matching 
control IgG experiments for each RBP experiment and for the same cell line). Did the authors check that there is no IgG binding at the 
sites that emerge from the analysis? Which of the enrichments shown in Supp File S5 would survive if IgG data was taken into account?

Reply: We thank the reviewer for raising this question, as it clearly highlights one of the major advantages of eCLIP data in comparison to 
standard CLIP procedures without an IgG control. However, as we are aware of this fact we already included processed peaks derived from the 
eCLIP pipeline which only cover regions with a significant signal over control ratio after stringent quality control and thus report only 
RBP overlaps with our data where binding is likely, which is true for all enrichments listed in Supp File S5. To make this clearer we 
adapted the methods section accordingly. (These binding sites are already quality controlled and show enrichment after normalization 
against IgG background, for more information on data generation and processing please refer to https://www.encodeproject.org/eclip/)

I understand the point about the eCLIP pipeline reporting processed results. Looking at Supp File S5 I see that the observed enrichment is very low including for KHDRBS1. So how can the authors claim that eCLIP provides corroborating evidence? Are the numbers reported in File S5 processed results? If so, the authors should report the original eCLIP values for the mitochondrial lookalikes of interest. 

I also note something else that is an issue. As can be seen from File S5 the RBPs that appear to bind the lookalikes (albeit with very low enrichment) are different in HepG2 and K562 cells. This means that there is a dependence on cell line. Is this dependence because the mRNAs whose introns contain the lookalikes are not expressed in one of the two cell lines or the mRNAs are expressed but the RBPs are absent or do not bind the corresponding location? Either way, given this cell type dependence which is highlighted by File S5 for binding the lookalikes I do not think that the authors can use the binding of a location by an RBP in one cell line to support binding of the same RBP in another cell line.

In our analysis we used this processed data and determined that peaks are *enriched* in overlap with MTLs, i.e. that there are more binding positions overlapping MTLs than in the surrounding DNA. This has nothing to do with the "enrichment" of reads in the foreground signal relative to the background control, which was already validated by the ENCODE authors as mentioned above. We believe that Reviewer#1 confounded these issues in his comments. We have modified the text to be more explicit about this distinction. We agree with Reviewer#1 that there might be a dependence on the cell line. However, this does not contradict our hypothesis. We proposed nimtRNAs to act as a type of splicing regulatory element, which are interpreted cell type dependently, mainly by the presence or absence of associated trans-factors.

Reviewer #1: In the same section that describes the LC-MS experiment, there is mention of mitochondrial tRNA lookalikes. These are 
presumably human lookalikes. But which ones? Where are they located? What is their sequence conservation compared to the true mitochondrial 
tRNAs? (See above about the presence of the mitochondrial segment that includes the seven tRNAs in non-intronic regions of the human 
genome.)

Reply: We want to reply to the comment that the nimtRNAs mentioned in the LC-MS were not properly introduced. In fact, the nimtRNAs 
employed were identical to those used for the experiments mentioned before, i.e. the YCNAW cluster of five nimtRNAs from mouse Myo3a 
intronic locus, which were already described in the manuscript. This is mentioned in the manuscript as follows: "
This approach utilized a biotinylated T7 transcript containing five nimtRNAs identical to those employed previously (i.e. YCNAW, see above) 
which was incubated with a nuclear extract generated from HEK 293T cells." (line 498)

I thank the authors for the clarification. This is a very important point that I missed in the first review. By introducing the five-tRNA-lookalike region that is located in mouse Myo3a in human cell lines the authors make the assumption that the same machinery is operational in human and mouse and with the same binding sites. Specifically, that the RBPs that bind this region to induce splicing in Myo3a in mouse exist and operate in exactly in the same manner in human cells. First, it is known that alternative splicing is a tissue specific process; see Yeo et al Genome Biology 2004. Second RBP expression is also tissue specific (see Baralle et al Nat Rev Mol Cell Biol. 2017 and also File S5 of this submission for evidence of same). By using human HEK293 cells the authors assume that the alternative splicing of Myo3a occurs in mouse kidney but they have not provided any evidence for that. Third, even if the alternative transcript of Myo3a was expressed in mouse kidney how do we know that it would be bound by the mouse counterparts of the human RBPs that emerged from the analysis of the human eCLIP data? Do the authors have any evidence to support this? Such evidence is necessary because we know from the work of Odom et al Nature Genetics 2007 that even for conserved transcription factors with tissue specific expression their binding sites are largely organism-specific and differ between human and mouse.

Given these dependencies, in my opinion, the nuclear extract should have been from a mouse tissue where the alternatively spliced products are present (and not from HEK293 cells) and incubation with nuclear extract from a mouse tissue in which the alternative isoform is absent should serve as control. Running the LC-MS in triplicate would have then shown whether there are any endogenous mouse RBPs that bind the region in question in one preparation but not in the other. Intersecting the LC-MS triplicates would have revealed the identity of the relevant RBPs and would have helped determine whether the authors' hypothesis is supported.

We agree with the reviewer that it would be interesting to compare mouse and human nimtRNA binding partners. As described in the manuscript, nimtRNAs carry different mutations compared to their mtRNA counterpart of the same organism. Thus, we do not suggest that nimtRNAs from mouse and humans bind different RBPs and thereby result in different effects on splicing in 293T cells, which - in fact - we showed by experimental evidence: all five nimtRNAs from the Myo3a locus in mouse (including nimtRNA^Trp^ in a reverse-complementary orientation), as well as all *bona fide* mouse mtRNAs, resulted in a splicing increase. Also, all different variants of the human nimtRNA^Ser2^ investigated showed the same effect on splicing as the human and the mouse bona fide mtRNA.

Furthermore, our mutational experiments with the mouse nimtRNA^Tyr^ and the human mtRNA^Gln r.-c.^ demonstrate that nimtRNAs exert their effect on splicing in a tRNA structure-dependent rather than a sequence-specific fashion. As the reviewer mentions, alternative splicing is cell type-specific. It is common knowledge that this is caused, to a large extent, by the different abundances of splicing-associated trans-acting factors recognizing intronic and exonic splicing regulatory elements (SREs), respectively. In our experimental set-up we determined that nimtRNAs increase alternative exon inclusion and constitutive splicing in HEK293T cells. Hence, we argue that nimtRNAs might exert different degrees of effects on splicing in other cell lines, such as for example mouse or human. This however does not at all contradict our data, since cell type- and context-dependent control of alternative and constitutive splicing have been reported in numerous studies to account for the presence and evolutionary conservation of SREs. Thus, using an extract from a different cell line, mouse or human, would potentially not recapitulate in vivo nimtRNA binding in HEK293T cells, not because there are potential differences in human and mouse spliceosomal machineries, but because even within the same organism different cell lines would be differently affected by nimtRNAs.

We also do not argue that the spliceosomal machinery associated with nimtRNA recognition identical in mouse and human. We do not infer that nimtRNAs result in the same effects on splicing in mouse kidney cells, simply because this would depend on the presence of the associated trans-factor. In respect to this we agree that additional experiments should be performed in the future which however exceed the scope of this study. In general, spliceosomal machineries are similar in mouse and humans and splicing-associated diseases have been investigated and treated in different mouse models. Among those diseases is spinal muscular atrophy which is treated by employing an antisense oligonucleotide targeting an intronic splicing silencer, similar to our nimtRNA splicing regulatory elements (Montes, 2019).

To thus include the points addressed by the reviewer we adapted the manuscript accordingly in the results and the discussion part: “We propose that mouse and human nimtRNAs are likely to be recognized by identical trans-factors in HEK 293T cells, since we showed that nimtRNA structure, rather than sequence, is responsible for observed effects.” (starting line 529) and “Whether or not this network is identical in mouse and humans remains to be investigated in the future. In general, splicing machineries are similar and splicing-associated diseases have been successfully explored and treated in different mouse models, including the Spinraza® (Nusinersen) antisense oligonucleotide targeting the intronic splicing silencer ISS-N1 (Montes 2019). Furthermore, nimtRNAs can be found in mouse and several other species (Telonis, 2015) suggesting a potential common pathway.” (starting line 677).

Reviewer #1: Finally, the eCLIP data analysis puts further into question the authors' very hypothesis. From the analyzed RBPs only three 
show enrichment at the mitochondrial tRNA lookalike sites: DHX30, G3BP1, and KHDRBS1. DHX30 is a component of the mitochondrial nucleoid 
and associates with mitochondrial DNA. If the mitochondrial tRNA lookalikes are exact copies of the mitochondrial tRNAs (as is the case of 
the lookalikes in the analyzed mouse cluster) it appears more likely that DHX30 is binding to the mitochondrial tRNA itself and not the 
intronic instance in the nuclear genome as the authors claim. The second RBP, G3BP1 is not known to be involved in splicing. Only the third 
RBP, KHDRBS1, is known to be involved in splicing but that is not its only role. Moreover the splicing connection hinges upon the 
assumption that the mitochondrial tRNA lookalike that is bound to KHDRBS1 is not expressed as a bona fide tRNA but only as part of an 
intron: this was not demonstrated for the human mitochondrial tRNA lookalikes that were analyzed by eCLIP, and, of course, still does not 
address the matter of the lookalikes that exist outside introns.

Reply: Please take into account that the enrichment we in show in Supp File S5 is adding an additional layer of information. One comes 
directly from eCLIP peaks, which, as stated above, are only called? if a significant enrichment of RBP binding over IgG control can be 
found for the region of interest. Additionally, we calculate the relative enrichment of RBP overlap with our data over background from 
binding sites in intronic regions and list those values in Supp File S5. The three mentioned RBPs are therefore not the only ones with 
significant peaks that overlap, but in addition they bind our sites more often than expected in comparison to other intronic regions.
We agree that the biological function of these interactions is so far not well characterized and as the reviewer points out we can only 
draw conclusions from already published functions of these RBPs, as investigations into this matter beyond the experiments already 
conducted require dedicated studies which are outside the scope of this manuscript. We agree with the reviewer that there are no 
indications that DHX30 is present in the nucleus and thus we excluded it from our results. Not much is known about the role of G3BP1 is in 
general, other than its ability to interact with RNA and DNA and its unwinding capabilities for duplexes of RNA/DNA and RNA/RNA, but it has 
been found in the nuclear compartment, which is why we did not exclude it.

As I mentioned above, File S5 does not show any enrichment that supports binding (the reported enrichment values are very low and no associated P-values are shown). More to the point, since the authors analyzed eCLIP, and despite the human/mouse incongruence they should be able to show the original raw human data: where exactly along the various tRNA lookalikes do the shown RBPs bind? where along the length of a tRNA is the binding motif? if all five of the tRNA lookalikes can lead to splicing it must mean that the binding motif should exist in all five tRNAs: so, is it a sequence motif or a structural motif? if the motif exists in the introns of other mRNAs and there is no eCLIP evidence of binding what would the implication be for the claimed role of the tRNA lookalikes in splicing?

We refer to our reply above concerning the enrichment of eCLIP peaks. Due to the size of eCLIP peaks (see reply above) and the low number of MTLs which show overlaps, we cannot infer a specific binding motif (sequence or structure) from this data alone. For motif analysis, all peaks of a specific RBP are taken together and enriched for sequence motifs compared to predefined background. With the small subset of MTL overlapping peaks such an analysis is simply not feasible and beyond the scope of this work. We therefore refer to recent work by Nostrand et al. 2020 and Dominguez et al. 2018 respectively, which although exhaustive, still covers only a subset of RBPs with available eCLIP data.

Reviewer #1 has stated that "the eCLIP data analysis puts further into question the authors' very hypothesis" referring to the sparsity of 
overlap between eCLIP and MS analyses.

Reply: We agree that it is impossible to exclusively map binding sites to either the mitochondrial tRNA or the intronic counterpart if they 
are exact sequence copies and no flanking sequence is taken into account. This is of course true for all regions in the genome which harbor 
identical or near identical sequences. As many RBPs are, however, known for their sequence specific binding preferences, binding to both 
sequences is equally likely, given that both RBP and target are available for binding which is the case here.

Do the authors have evidence to support the implicit claim that the sequence-specific binding applies to the RBPs that they singled out in their analyses?

Furthermore, the downloaded 
eCLIP peaks undergo stringent quality control, including removing of mappings to repeat regions and minimum coverage of peak regions by 
uniquely mappable reads as stated in https://www.encodeproject.org/documents/739ca190-8d43-4a68-90ce- 
1a0ddfffc6fd/@@download/attachment/eCLIP_analysisSOP_v2.2.pdf.

See my point above about the claimed enrichments.

We refer to above reply which explains the difference in enrichment of signal over background versus peaks overlapping MTLs or other regions.

Moreover the splicing connection hinges upon the assumption that the mitochondrial tRNA lookalike that is bound to KHDRBS1 is not expressed 
as a bona fide tRNA but only as part of an intron: this was not demonstrated for the human
mitochondrial tRNA lookalikes that were analyzed by eCLIP, and, of course, still does not address the matter of the lookalikes that exist 
outside introns.

Reply: We thank the reviewer for bringing up this issue. We took this into account which is why we included an additional 10 nucleotides up- and 
downstream of the nimtRNATyr from the endogenous Myo3a locus in mouse in the transcript used for the electrophoretic mobility shift assay. 
This is now included in the text as follows: "To this end, a transcript of nimtRNATyr including an extra 10 nucleotides at the 5' and 3' 
termini, in order to not resemble processed tRNA ends, was radioactively labeled and incubated with increasing concentrations of PTBP1 
protein." (Line 518)

As I mentioned above the presence of the lookalikes outside introns needs an explanation. If inserting the five lookalike region into a reporter assay is sufficient to cause alternative splicing it is not much of a stretch to expect that insertion of the a tRNA lookalike in regions that are in intergenic space and are transcribed could control the products from those regions. The authors find some of the lookalikes in lncRNAs: is there evidence of alternative splicing for these lncRNAs? is there evidence of binding for the RBPs of interest to those regions? These are relevant questions that have not been addressed.

Again, regarding function of mtRNA lookalikes located outside introns we refer to our reply above.

Reviewer #2: The authors have answered my comments in a well-justified and satisfactory manner. The manuscript is clearer with the modifications and constitutes an important contribution to the literature.

We thank the reviewer for her/his time and efforts.

**Third round of review**

**Reviewer 2:**

Comments to Author:

In my opinion, the authors have addressed reviewer 1's comments in a reasonable manner. The only issue I have with the response is regarding the miR-124 experiment:

Since the sequence of the human and mouse miR-124 are identical, we have no evidence that the miRNA detected (presumed by the authors to be the mouse miRNA from the transfected construct) is not the endogenous human miRNA. The blot provided in the response letter, which serves to show that no endogenous miRNA is present since no band is present in the lane 1+ at the level of the D arrow, is not convincing. Indeed, the D portion of the blot is cut off for lanes 1 and 2 (in fact there seems to be a band in lane 2), and the quality of the blot is not great for lanes 1 and 2. So to me, the miR-124 experiment is not providing any useful information for the reader.

I would suggest that the authors simply remove the miR-124 experiment from the paper (they already have the SNORD115 control).
